# Fibromyalgia and Chronic Fatigue Syndromes: A systematic review and meta-analysis of cardiorespiratory fitness and neuromuscular function compared with healthy individuals

**DOI:** 10.1371/journal.pone.0276009

**Published:** 2022-10-20

**Authors:** Fabio Zambolin, Pablo Duro-Ocana, Azmy Faisal, Liam Bagley, William J. Gregory, Arwel W. Jones, Jamie S. McPhee

**Affiliations:** 1 Department of Sport and Exercise Sciences, Manchester Metropolitan University, Manchester, United Kingdom; 2 Manchester Metropolitan University Institute of Sport, Manchester, United Kingdom; 3 Department of Life Sciences, Manchester Metropolitan University, Manchester, United Kingdom; 4 Department of Anaesthesia, Manchester University NHS Foundation Trust, Manchester, United Kingdom; 5 Faculty of Physical Education for Men, Alexandria University, Alexandria, Egypt; 6 Rheumatology Department, Salford Care Organisation, Northern Care Alliance NHS Foundation Trust, Salford, United Kingdom; 7 Faculty of Health, Psychology and Social Care, Manchester Metropolitan University, Manchester, United Kingdom; 8 Respiratory Research@Alfred, Monash University, Melbourne, Australia; Syddansk Universitet, DENMARK

## Abstract

**Objective:**

To determine cardiorespiratory fitness and neuromuscular function of people with CFS and FMS compared to healthy individuals.

**Design:**

Systematic review and meta-analysis.

**Data sources:**

PubMed, Medline, CINAHL, AMED, Cochrane Central Register of Controlled Trials (CENTRAL), and PEDro from inception to June 2022.

**Eligible criteria for selecting studies:**

Studies were included if presenting baseline data on cardiorespiratory fitness and/or neuromuscular function from observational or interventional studies of patients diagnosed with FMS or CFS. Participants were aged 18 years or older, with results also provided for healthy controls. Risk of bias assessment was conducted using the Quality Assessment Tool for Quantitative Studies (EPHPP).

**Results:**

99 studies including 9853 participants (5808 patients; 4405 healthy controls) met our eligibility criteria. Random effects meta-analysis showed lower cardiorespiratory fitness (VO_2_max, anaerobic threshold, peak lactate) and neuromuscular function (MVC, fatigability, voluntary activation, muscle volume, muscle mass, rate of perceived exertion) in CFS and FMS compared to controls: all with moderate to high effect sizes.

**Discussion:**

Our results demonstrate lower cardiorespiratory fitness and muscle function in those living with FMS or CFS when compared to controls. There were indications of dysregulated neuro-muscular interactions including heightened perceptions of effort, reduced ability to activate the available musculature during exercise and reduced tolerance of exercise.

**Trail registration:**

**PROSPERO registration number:** (CRD42020184108).

## Introduction

The incidence of Fibromyalgia Syndrome and Chronic Fatigue Syndrome (FMS, CFS) is estimated to be 1.0–2.7% of the worldwide population, with 2–3 times higher prevalence for females than males [1–5]. Both syndromes are difficult to identify and diagnosis usually follows recommendations of 2010 FMS diagnostic criteria and the 2015 IOM diagnostic criteria for ME/CFS [6, 7]. However, these criteria are not entirely objective [8–11] and concerns remain about their implementation [12]. This leads to delayed diagnosis which impacts on quality of life for those affected [10] before clinical management plans can be implemented.

CFS and FMS conditions overlap substantially through the shared symptom of chronic fatigue which occurs in the absence of intense or prolonged physical activity and is not necessarily ameliorated by rest [13]. FMS also causes widespread musculoskeletal pain in the absence of any structural or morphological abnormalities of skeletal muscle tissue from histological and imaging analysis [9, 14, 15].

The origin and pathophysiology of these syndromes are not fully understood [2, 16]. They may arise due to “sensitisation” of central and/or peripheral nervous systems to sensory stimuli [14, 17–20]: afferent signals originating in the periphery may be amplified ahead of processing in the central nervous system [21] leading to a hypersensitisation of typical somatosensory stimuli during everyday activities. If this were the case, then physical activity should aggravate symptoms, which is consistent with patient reports of discomfort during exercise [22, 23], reduced exercise tolerance [24–26] and relatively low habitual physical activity levels [27, 28]. Aberrant somatosensory signalling may reduce cardiorespiratory fitness and neuro-muscular function and increase perceptions of effort to levels excessive for the respective physiological strain, but this remains unclear due to conflicting reports, heterogeneity of methodological approaches or few studies including relevant outcome data in the literature. Previous reviews investigated cardiorespiratory fitness and neuromuscular function in CFS and FMS, finding that peak oxygen consumption was reduced while muscle strength and rate of perceived exertion were increased [5, 25, 29, 30]. However, the possible underlying physiological processes of the lower physical function remain unclear.

The aim of this systematic review and meta-analysis was to determine physical performance of people living with CFS and FMS compared with healthy controls, and to identify possible underlying physiological processes associated with reduced physical performance of the patient groups. Physical performance was classified into two main components: cardiorespiratory fitness and neuromuscular function. Cardiorespiratory fitness was taken as the peak rate of oxygen uptake (VO_2_peak: measured during incremental exercise) and anaerobic threshold. Secondary indicators were collected where available to understand possible causes of reduced cardiorespiratory fitness, including peak lactate measurements, peak heart rates and ratings of perceived exertion (RPE). Neuromuscular function was characterised as maximal voluntary contraction (MVC), performance fatigability [31], voluntary activation (the ability to fully activate available motor units during MVC), alongside measures of skeletal muscle mass, and rate of perceived exertion.

## Methods

The present systematic review is reported in accordance with the Preferred Items for Systematic Reviews and Meta-Analyses (PRISMA) guidelines [32]. Methods of the analysis and inclusion criteria were pre-specified and registered on the International Prospective Register of Systematic Reviews (PROSPERO: protocol-CRD42020184108). Full ethical approval was given from the Science and Engineering Research Ethics and Governance Committee at Manchester Metropolitan University (reference number 23820).

### Eligibility criteria

Studies were included if they were observational or interventional designs providing data for patients with diagnosed CFS or FMS. Participants needed to be aged 18 years or older and studies needed to provide results for healthy controls. For interventional studies only the baseline data were included.

VO_2_peak, ventilatory threshold and/or lactate measurements were classified as cardiorespiratory fitness outcomes. Peak heart rate and RPE during incremental exercise were also recorded to indicate whether incremental exercise tests were terminated before the estimated peak heart rate was achieved. MVC, fatigability, voluntary activation, muscle mass or volume were recorded as neuromuscular outcomes. Fat mass and RPE during fatigue tests were also recorded.

Studies were excluded if presenting results from animal models, if they included participants aged younger than 18 years, if they were not peer reviewed or not written in English language.

### Search strategy

PubMed, Medline, CINAHL, AMED, Cochrane Central Register of Controlled Trials (CENTRAL), and PEDro databases were searched using keywords and medical subject headings structured in a PICO framework (S4 File). Initial literature searches were conducted between April—June 2020 and updated then updated to June 2022.

### Selection process

Search results were collated in referencing software (EndNote X9 –Clarivate Analytics) and shared between two independent researchers (FZ and PDO). Records were removed if titles and abstracts clearly showed they were not eligible. Full texts of the remaining records were screened by the two independent researchers (FZ and PDO). Disagreements about whether a study should be included were resolved by discussion with a third researcher (JM).

### Data items and collection process

Included studies were read in full and the relevant data were entered to a Microsoft© Excel spreadsheet. The following data were extracted from each included study: type of diagnosis (CFS and/or FMS) publication details, sample size, subjects’ characteristics (age-sex), diagnostic criteria, participants’ matching type, outcome assessments and details of test protocols.

If data from the studies were not available, or were incomplete, they were not included for further analysis. Where data were reported only in a figure format without any possibility to precisely extract their values, the corresponding author of that study was contacted for the numerical data. If the data could not be provided or no answer was received, the study was excluded from further analysis.

### Risk of bias assessments

Risk of Bias was assessed using the Quality Assessment Tool for Quantitative studies (EPHPP) [33] covering six domains: 1) selection bias (representation of the target population), 2) study design, 3) confounding factors, 4) blinding, 5) data collection method and, 6) withdrawal. Components 2 and 4 were excluded since they relate primarily to intervention studies [34]. Studies were evaluated for components 1, 3, 5 and 6 as strong, moderate, or weak depending on the number of parts evaluated as weaker (i.e., two or more weak evaluations was recorded as a low study quality; only one, or no weak points was recorded as high study quality).

A risk of bias tool from Nijs et al. [5] was applied to improve the quality ratings of the confounding domains of the EPHPP tool across eight domains: 1) presence of a priori power calculations; 2) controls comparable for age; 3) gender; 4) body height or weight; 5) physical activity level; 6) presence of sedentary subjects; 7) blinded assessments; and 8) medications wash-out prior to the tests. If reaching a score lower than 50% on the specific tool from Nijs [5], the confounding domain was rated as weak. This process helped to define specific cofounding factors as applied in previous reviews [5, 29] (S4 File).

Sensitivity analysis [35] was completed to examine possible differences in outcomes based on the risk of bias and a further subgroup analysis was conducted on the low risk of bias studies if heterogeneity was still present (I^2^>40%).

### Data synthesis

Continuous data were pooled using a random-effect meta-analysis using the inverse variance method, with the measure of effect between patient and control groups being standardized mean differences (SMD) and 95% confidence intervals. Effect size thresholds were considered at 0.2, 0.5 and 0.8 for small, moderate and large effect sizes, respectively [34]. Where results were stratified by sex, the male and female samples were combined to derive a single effect [36]. Combined means and standard deviations for Wåhlén et al. [37], Sargent et al. [38] and Vermeulen et al. [39, 40] were calculated using the method described in the Cochrane Handbook [36]. Studies reporting the median and interquartile range or minimum and maximum range were transformed into mean and SD from the Microsoft© Excel spreadsheet tool provided by Wan et al. [41]. Heterogeneity was classified in four domains: 1) might not be important (I^2^ = 0% - 40%), 2) may represent moderate heterogeneity (I^2^ = 30% - 60%), 3) may represent substantial heterogeneity (I^2^ = 50% - 70%), or 4) considerable heterogeneity (I^2^ = 70–100%) using the criteria proposed by Higgins et al. [42]. Initially all the samples for each outcome were included within a single model, but if heterogeneity was considered important (I^2^ >40%) further subgroup analysis was conducted on the potential source of heterogeneity. All meta-analyses and subgroup analyses were performed using RevMan 5.4 software. The a priori level of significant difference was set at p<0.05. Pooled data are presented as Cohen’s d standardized mean difference (SMD) with 95% confidence intervals. Our primary approach was to combine results of patients with CFS and FMS for comparison to healthy controls, given the overlap that is present between the two syndromes [43–45] and the small sample sizes. This approach was also implemented in a previous systematic review with meta-analysis [46]. However, we have also provided a subgroup analysis differentiating studies from FMS and CFS for each available outcome measurement (Figs 3 and 4 and S3 File).

## Results

### Selection of studies

**Fig 1:**
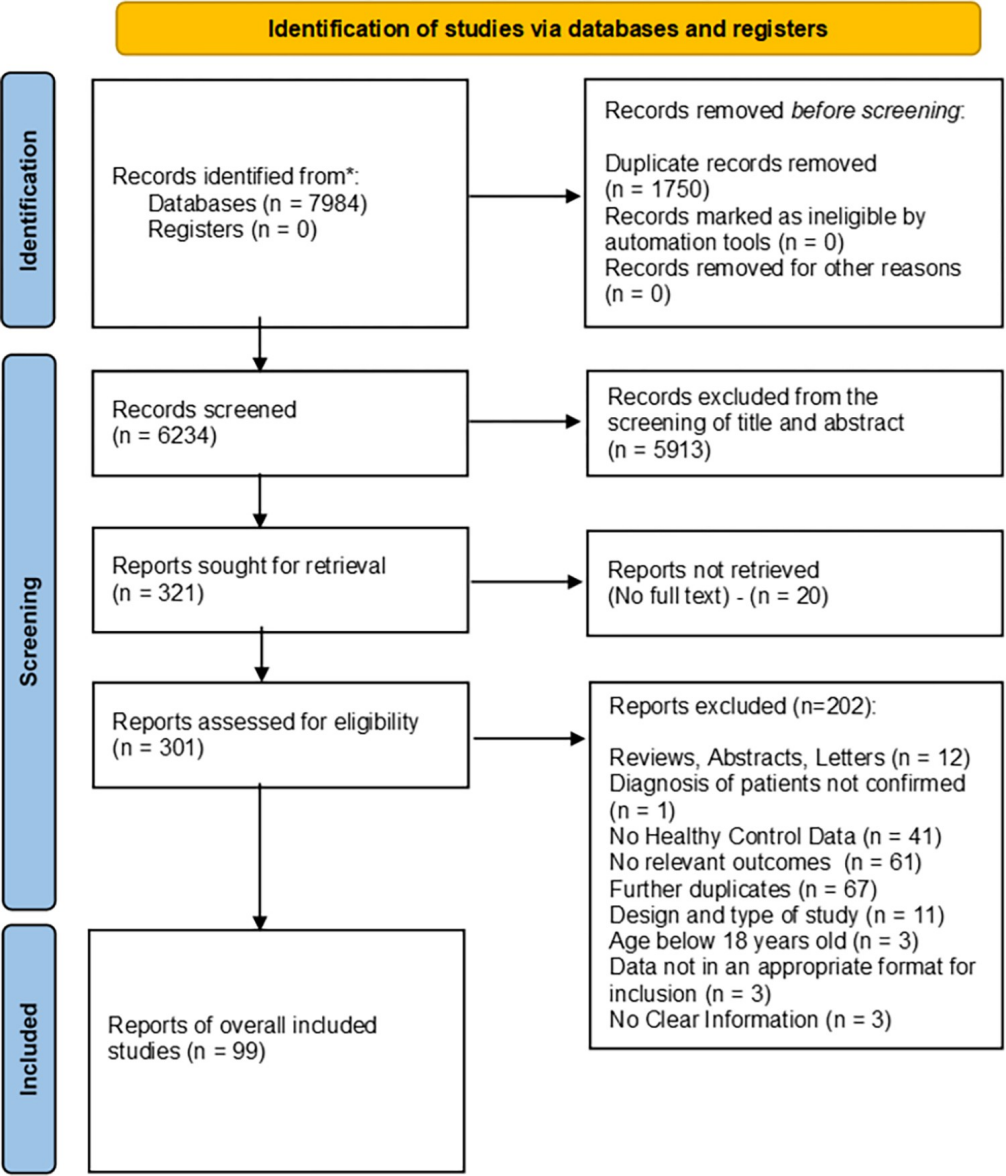
The 2020 prisma flow diagram. Flow diagram for selection and inclusion of studies.

**Fig 2:**
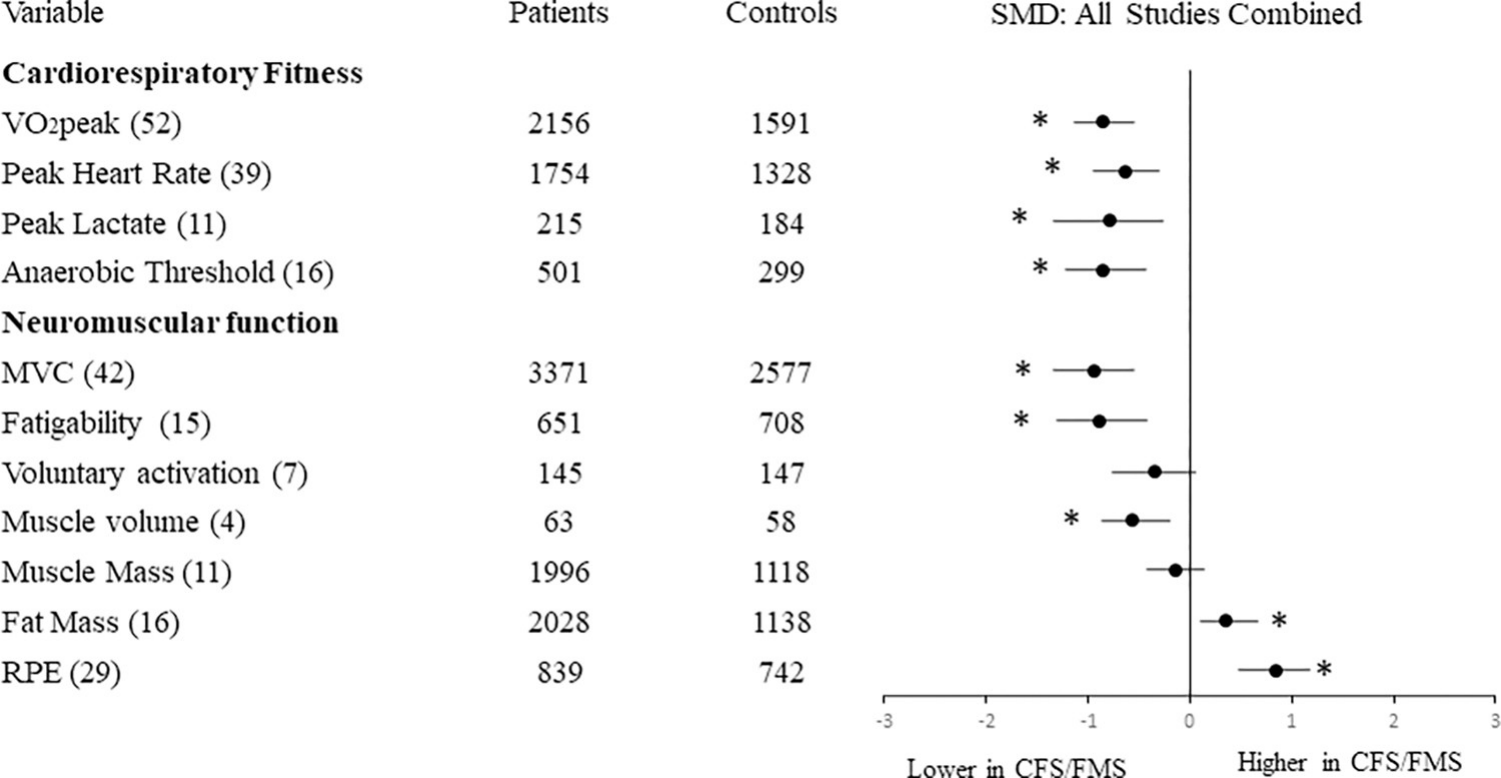
Effect size of differences in cardiorespiratory and neuromuscular outcomes in CFS and FMS compared with healthy controls. Numbers in brackets indicate the number of studies reporting each variable. Abbreviations: MVC: Maximal Voluntary Contraction; RPE: rating of perceived exertion. *p<0.05 for overall effect size.

A total of 7984 records were identified and 99 of those met the inclusion criteria (Fig 1). From the 99 studies, 40 of them reported cardiorespiratory fitness outcomes, 28 reported muscle function outcomes, 10 reported body composition outcomes and the remaining 21 studies included outcomes of cardiorespiratory fitness and muscle function and body composition. Summary of overall results are reported in Fig 2 and S2 File.

### Cardiorespiratory fitness

#### VO_2_peak

Data from 52 studies showed a significant difference between patient and control groups, with a large effect size (SMD = -0.86, 95%CI = -1.04 to -0.69) revealing lower VO_2_ peak values for patients. Heterogeneity was classified as substantial (I^2^ = 80%).

Studies with lower risk of bias included 875 patients and 643 healthy controls (I^2^ = 51%), again revealing lower VO_2_peak values for patients compared with healthy controls but with a moderate effect size (SMD = -0.61; 95%CI = -0.77, -0.46).

#### Anaerobic Threshold (AT)

The 16 included studies showed that patients had significantly lower AT values than controls with a large effect size (SMD-0.86, 95%CI = -1.12 to -0.60). Heterogeneity was classified as substantial (I^2^ = 57%).

Studies with low risk of bias included 412 patients and 214 healthy controls (I^2^ = 66%). They also showed lower AT for patients compared with healthy controls with large effect size (SMD = -0.80; 95%CI = -1.15, -0.46).

#### Peak lactate

Data from 11 studies showed that patients had significantly lower peak lactate values than controls (Z = 3.44, p = 0.00001), with a large effect size (SMD = -0.84, 95%CI = -1.33 to -0.36). Heterogeneity was classified as substantial (I^2^ = 80%).

Studies with low risk of bias included 82 patients and 81 healthy controls (I^2^ = 41%). They also showed lower peak lactate for patients, but with small effect size (Z = 1.50, p = 0.13; ES = -0.27; 95%CI = -0.62, 0.08).

#### Peak heart rate

Thirty-nine studies showed that patients had significantly lower peak HR than controls at the end of incremental exercise, even when normalized to age. There was a moderate-high effect size (SMD = -0.64, 95%CI = -0.77 to -0.50) and heterogeneity was classified as substantial (I^2^ = 58%).

Studies with low risk of bias included 1025 patients and 813 healthy controls (I^2^ = 26%). They also showed lower peak HR for patients, but with large effect size (SMD = -0.57; 95%CI = -0.70, -0.44).

### Neuromuscular function

#### Maximal Voluntary Contraction (MVC)

Data from 42 studies showed significant difference between groups, revealing lower MVC values for patients and a large effect size (SMD = -0.93, 95%CI = -1.12 to -0.75). Heterogeneity was classified as substantial (I^2^ = 89%).

Studies with low risk of bias included 864 patients and 667 healthy controls (I^2^ = 39%). They showed a moderate decrease of MVC for patients (SMD = -0.63; 95%CI = -0.78, -0.49).

#### Fatigability

Fifteen studies were included, showing that patients were more fatigable than controls with a large effect size (SMD = -0.88, 95%CI = -1.19 to -0.57). Heterogeneity was classified as substantial (I^2^ = 84%).

Studies with low risk of bias included 135 patients and 85 healthy controls (I^2^ = 70%). They also showed patients to be more fatigable than controls with moderate effect size; (SMD = -0.47; 95%CI = -0.77, -0.18).

#### Voluntary activation

Seven studies considered voluntary activation, providing data of 145 patients and 147 healthy controls. Patients had lower voluntary activation than controls with moderate effect size (SMD = -0.34, 95%CI = -0.70 to 0.03). Heterogeneity was classified as moderate (I^2^ = 54%). Further sub-group analysis was not completed due to the low number of available studies.

#### Muscle mass

Data from 11 studies showed similar muscle mass for patients and controls and small effect size (SMD = -0.14, 95%CI = -0.30 to 0.02). Heterogeneity was classified as substantial (I^2^ = 70%).

Studies with low risk of bias included 173 patients and 171 healthy controls (I^2^ = 0%). They showed no difference in muscle mass for patients compared to healthy controls (SMD = -0.00; 95%CI = -0.21, 0.21).

#### Muscle volume

Four studies considered thigh muscle volume (quadriceps, hamstrings), providing data of 63 patients and 58 healthy controls. Patients had lower muscle volume than controls (Z = 2.96, p = 0.003), with moderate effect size (SMD = -0.56, 95%CI = -0.92 to -0.19). There was no indication of heterogeneity (I^2^ = 0%). Further sub-group analysis was not completed due to the low number of available studies.

#### Fat mass

Data from 16 studies showed higher fat mass for patients compared to controls with moderate effect size (SMD 0.36, 95%CI = 0.22 to 0.50). Heterogeneity was classified as moderate (I^2^ = 55%).

Studies grouped as low risk of bias included 184 patients and 182 healthy controls (I^2^ = 0%). They showed similar fat mass for patients and controls with small effect size (SMD = 0.21; 95%CI = 0.00, 0.41).

### Perception indicators

#### Rate of Perceived Exertion (RPE)

Mean and Peak RPE outcomes were available for 29 studies covering cardiopulmonary fitness and muscle function. RPE was significantly higher for patients than controls for the given workload, with moderately large effect size (SMD 0.84, 95%CI = 0.60, 1.08). Heterogeneity was classified as substantial (I^2^ = 77.0%).

Studies with low risk of bias included 330 patients and 336 healthy controls (I^2^ = 53%). They showed higher RPE for patients than controls and a large effect size (SMD = 1.06; 95%CI = 0.81, 1.31).

#### Comparison of results for FMS and CFS

**Fig 3:**
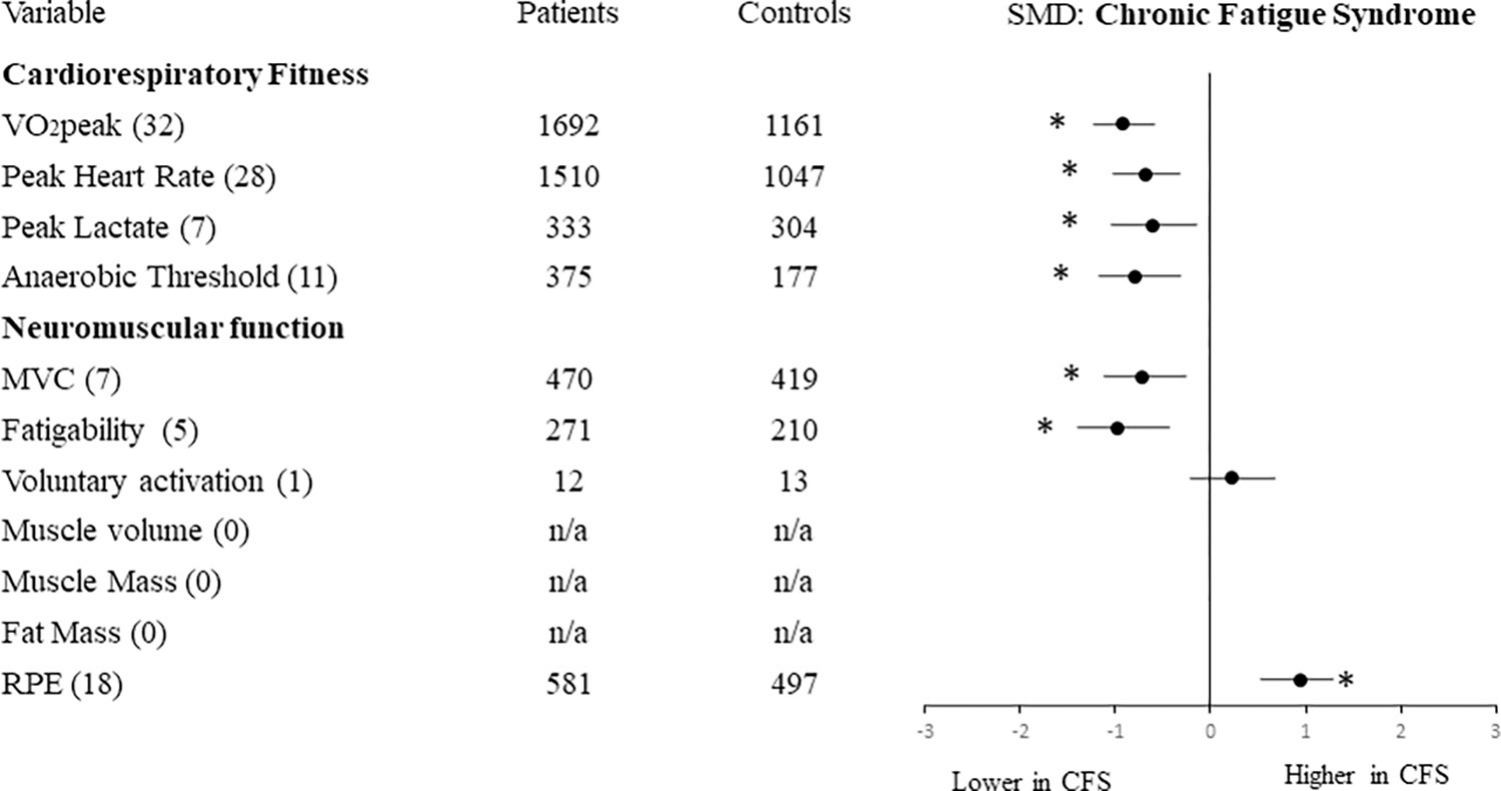
Effect size of differences in cardiorespiratory and neuromuscular outcomes in CFS compared with healthy controls. Numbers in brackets indicate the number of studies reporting each variable. Abbreviations: MVC: Maximal Voluntary Contraction; RPE: rating of perceived exertion. *p<0.05 for overall effect size.

**Fig 4:**
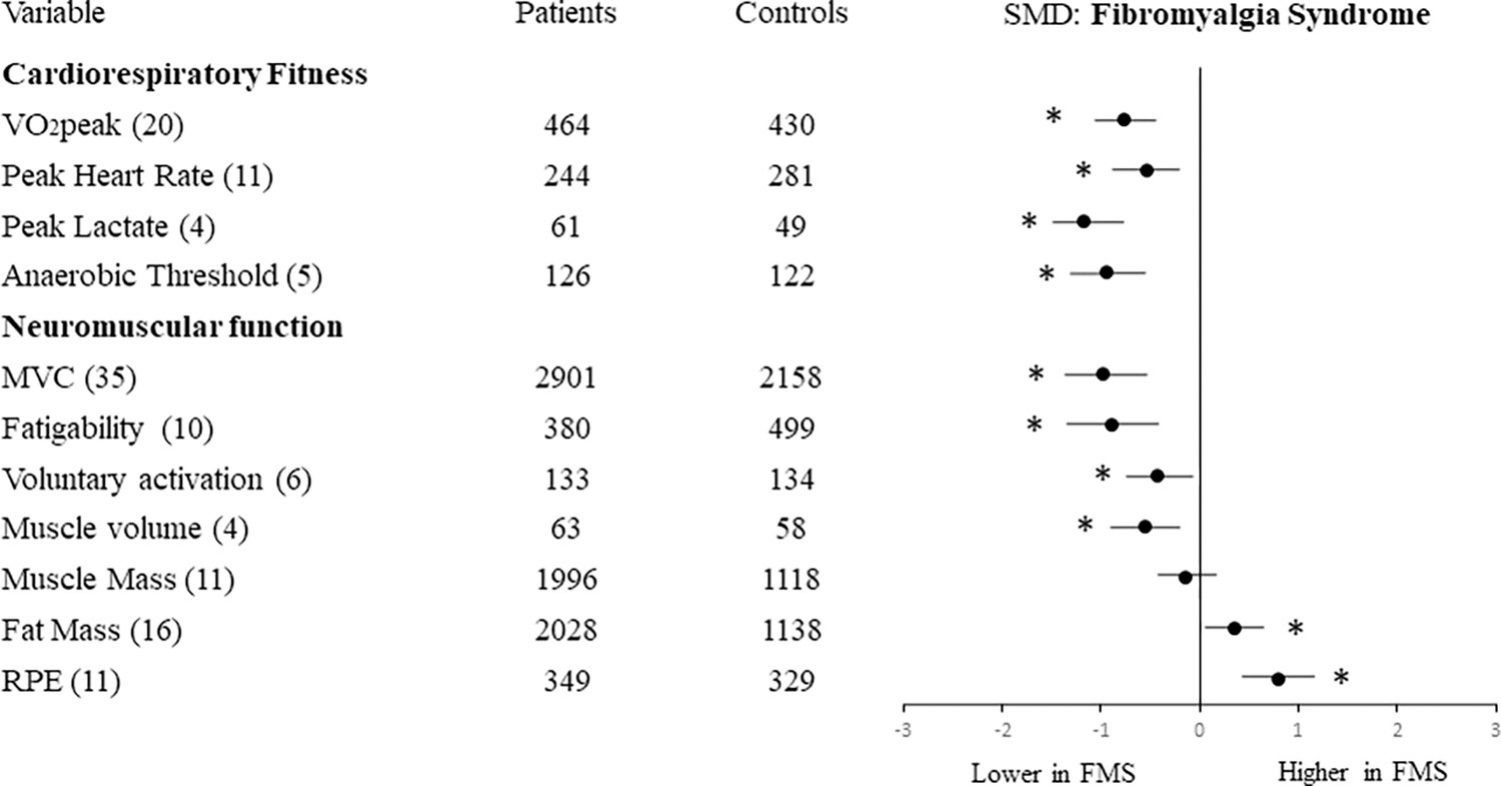
Effect size of differences in cardiorespiratory and neuromuscular outcomes in FMS compared with healthy controls. Numbers in brackets indicate the number of studies reporting each variable. Abbreviations: MVC: Maximal Voluntary Contraction; RPE: rating of perceived exertion. *p<0.05 for overall effect size.

Figs 3 and 4 present the results for FMS and CFS separately. Sub-group analysis was performed to determine whether, compared with their respective healthy controls, the functional reductions of FMS were different from those of CFS. The results showed no significant differences between the two (FMS and CFS) for cardiorespiratory fitness or neuromuscular function outcomes, except for MVC which was relatively lower for FMS than CFS (p = 0.04).

## Discussion

### Summary of the main findings

This systematic review and meta-analysis included 99 studies detailing results of CFS and/or FMS patients compared with their healthy counterparts. The results showed lower VO_2_peak, AT, and MVC for patients compared with controls. We further sought to understand the underlying causes of their lower physical function by exploring the possibility of patients having lower muscle mass, reduced ability (or willingness) to fully activate motor pathways, or increased perceptions of effort during exercise. The available evidence showed a discordance of perceived to physical exertion as exercising patients reported higher ratings of perceived exertion compared with controls for a given workload or heart rate. Patients also had lower voluntary activation and fatigued more quickly than controls.

Our sub-group analysis showed negligible differences between CFS and FMS for their relative reductions of cardiorespiratory or neuromuscular outcomes, except for a lower MVC in FMS compared with CFS (S3 File). However, few studies of CFS reported skeletal muscle mass, fat mass or voluntary activation so conclusions on these outcomes are limited.

This systematic review and meta-analysis provides new insights into the possible underlying determinants of reduced cardiorespiratory and muscle function of patients with CFS and FMS. Previous reviews [5, 25, 29, 30] reported physical function in CFS and/or FMS. Of these, Franklin et al. [29] performed a meta-analysis of 32 studies reporting VO_2_peak outcomes in CFS, and Gaudreault et al. [30] considered cardiorespiratory fitness of FMS with a narrative review: both reported lower cardiorespiratory fitness of patient groups. More recently, Barhorst et al. [25] reported higher RPE during exercise for patients with CFS and FMS, alongside lower heart rates at volitional exhaustion compared with healthy controls. We are aware of only one previous systematic review of muscle function impairments in CFS [5], but no others considering FMS or including wider aspects of muscle function such as fatigability and VA outcomes.

### Cardiorespiratory fitness

Some studies utilised indirect assessments of VO_2_peak by extrapolating data points obtained from submaximal exercise testing, but indirect assessments have greater errors of estimates and accordingly the reported results varied considerably from one study to the next. Direct tests of VO_2_peak are more accurate and require participants to continue to exercise through progressively increasing intensities until volitional exhaustion. From the reviewed literature it was clear that few patients satisfied the criteria (ACSM guidelines [47]) for achieving a true VO_2_max and they terminated exercise at lower peak workloads than controls.

The AT was usually estimated during tests of VO_2_peak, and it represents the threshold beyond ‘steady-state’ at which glycolytic rates rise. Indicators of AT include blood lactate above 4 mmol/L or the gas exchange or ventilatory thresholds identified from the non-linear relationship of VCO_2_ to VO_2_ [48, 49]. Regardless of study quality, risk of bias or methodological approaches, the results were consistent with those of VO_2_peak, where values for AT were lower for patients than controls. This suggests the impairments affecting cardiorespiratory fitness of patients are not only evident during very intense activity where high motivation is required, but they are also evident during moderate intensity activities.

Since VO_2_peak and AT are highly adaptable: decreasing with periods of inactivity and increasing with periods of exercise training, it may be speculated that values for both were lower for patients than controls due to habitual sedentary lifestyles [5, 50] or pharmacological effects on the patient groups [5]. However, this conclusion is not fully supported by the available data, given the other indicators that patients were not able to exercise at the high intensities required to achieve VO_2_peak. These included lower peak heart rates and peak blood lactate levels during exercise for FMS and CFS compared with healthy controls. To account for possible confounding effects, some studies matched groups for habitual physical activity levels or included a pharmacological wash-out period prior to participation. A more detailed look at these studies revealed that patients had lower VO_2_peak and MVC than controls even after controlling for physical activity levels or after pharmacological wash-out (S3 File).

### Muscle function

Different assessments of MVC were applied across the different studies, with some using isometric and others dynamic concentric contractions of upper body or lower body muscle groups. These methodological differences did not change the outcome of lower muscle strength expressed as MVC for patients compared with healthy controls. Force production, being a primary function of skeletal muscle, is determined by the total muscle mass (or, more precisely the physiological cross-sectional area) and the level of voluntary activation which represents the ability to activate all available motor units [51]. Muscle mass estimated by dual-energy x-ray absorptiometry (DXA) or bioimpedance analysis (BIA) was similar for patients and controls. However, the more accurate approach [52] to estimating muscle size by magnetic resonance imaging showed lower muscle volume for patients than controls. Voluntary activation was lower for patients than controls, suggesting that patients were unable or unwilling to produce a true maximal force contraction. Lower voluntary activation may indicate deficits of central motor pathways through the motor cortex, upper motor neurons and peripheral motor neurons [53]. Such neural deficits are possible, given the known changes of central and peripheral sensitization in FMS and CFS patients [9, 18, 54] that may affect motor output.

Our results showed that FMS and CFS patients fatigued more quickly during exercise than their healthy counterparts, and this remained the case after considering only those studies with lowest risk of bias. Notably, as was the case for other measurements of muscle function, fatigability (expressed as performance deterioration) [31] was measured in different ways across studies. The lack of consistency across studies makes it difficult to determine how skeletal muscle fatigue relates to the experience of generalized fatigue, which is a primary manifestation of CFS, and FMS that requires further investigation [55].

The accelerated onset of exercise-related fatigue alongside the perceptions of generalized fatigue highlights the different ways in which fatigue is experienced by FMS and CFS patients. There are some possible overlaps with interaction effects linked to sensitized afferent pathways that exaggerate mechano- and metabo- receptor activity, or brain regions receiving and interpreting information from distal body parts. This concept of heightened afferent feedback could contribute not only to generalized and exercise-related fatigue but also to the relative muscular weakness of patients [14]. It may also contribute to heightened rates of perceived exertion and pain during exercise reported by patients [25, 46].

### Implications for research and practice

Chronic fatigue is a primary feature of CFS and FMS. It can impact greatly on those affected by reducing their social and economic interactions. Alongside the generalized fatigue, our findings reveal the extent of reduced physical function of patients which occurs in excess of that expected from sedentary living. The reasons for the generalized fatigue and reduced physical function remain largely unknown. Future studies should aim to understand what causes the heightened perceptions of effort during exercise and how this relates to fatigue. Possible mechanisms may include sensitized peripheral afferents, including the III-IV muscle afferents [14, 56, 57], and/or regions of the central nervous system that receive those afferent signals.

If effective therapies are not applied, the individuals with CFS and FMS will continue to experience a poorer quality of life and will be at greater risk of inactivity-related poor-health conditions. To this end, a recent study showed increased incidence of skeletal muscle weakness and low muscle mass in relatively young populations of people with FMS placing them at greater risk of sarcopenia [58]. The most effective way of improving cardiorespiratory fitness and muscle mass/function is regular intense exercise and there is evidence that exercise may be effective for individuals with CFS and FMS [59–62]. Future studies should investigate whether regular intense exercise familiarizes patients to afferent signals from exercising limbs to improve exercise tolerance by reducing the heightened perceptions of effort and fatigue. However, the benefits of training will depend on a person’s commitment to training and the fact that CFS and FMS patients experience heighted perceived exertion may reduce their tolerance of the higher intensity workloads which are most effective for improving physical fitness [63].

## Conclusions

Overall, our results demonstrate lower cardiorespiratory fitness and muscle function of individuals with CFS and FMS compared with healthy controls. There were indications of dysregulated neuro-muscular interactions including heightened perceptions of effort, reduced ability to activate the available musculature during exercise and reduced tolerance of exercise. Future work should investigate whether impairments of the nervous system cause these changes.

## Supporting information

S1 File Risk of bias table. (PDF)

S2 File Characteristics of the studies. Descriptive tables of all the studies included in the systematic review and meta-analysis.
(PDF)

S3 File Statistical and sensitivity analysis. RevMan full graphs and sensitivity analysis for all the outcomes included in the systematic review and meta-analysis.
(PDF)

S4 File Full research strategy. (PDF)

S5 File Prisma checklist. (PDF)

S6 File List of abbreviations. (PDF)
