# Supplementary material for: Fibromyalgia and Chronic Fatigue Syndromes: A systematic review and meta-analysis of cardiorespiratory fitness and neuromuscular function compared with healthy individuals
Source: PLoS One. 2022 Oct 20;17(10):e0276009. doi: 10.1371/journal.pone.0276009 (PMC9584387; doi:10.1371/journal.pone.0276009)
Supplement: S1 File — (PDF) [file pone.0276009.s001.pdf]

Study Characteristics				EPHPP Quality Assessment				
Year	Name	Type	Selection of Bias	data collection	Drop Out	*Confounders*	*Total*	*High/Low Risk*
2015	Aerenhouts[1]	CFS	2	2	3	3	3	High
2017	Acosta -Manzano[2]	FMS	2	2	3	3	3	High
2020	Andretta[3]	FMS	2	1	1	2	2	Low
2010	Aparicio[4]	FMS	2	2	3	3	3	High
2011	Aparicio[5]	FMS	2	2	3	3	3	High
2013	Aparicio[6]	FMS	2	2	3	3	3	High
2015	Aparicio[7]	FMS	2	3	3	3	3	High
2013	Bachasson[8]	FMS	2	2	3	3	3	High
2014	Balbaloglu[9]	FMS	2	3	3	3	3	High
2013	Bardal[10]	FMS	1	1	3	3	3	High
2015	Bardal[11]	FMS	2	2	2	2	1	Low
2001	Bazelmans[12]	CFS	2	1	2	2	1	Low
1998	Blackwood[13]	CFS	2	2	3	3	3	High
1999	Borman[14]	FMS	1	1	3	2	2	Low
2021	Berardi[15]	FMS	2	2	2	2	2	Low
2019	Bouquet[16]	CFS	1	2	2	1	1	Low
2018	Ceron Lorente[17]	FMS	2	2	3	3	3	High

2003	Cook[18]	CFS	1	2	3	1	2	Low
2006	Cook[19]	CFS/FMS	2	2	1	2	1	Low
2012	Cook[20]	CFS/FMS	1	1	2	1	1	Low
2022	Cook[21]	CFS	2	1	2	1	1	Low
2011	Da Cunha[22]	FMS	2	2	3	2	2	Low
2000	De Becker[23]	CFS	3	3	1	2	3	High
2007	Dinler[24]	FMS	3	2	3	3	3	High
2009	Dinler[25]	FMS	2	2	3	3	3	High
2001	Elert[26]	FMS	2	2	3	3	3	High
2002	Farquhar[27]	CFS	2	3	2	3	3	High
2000	Fulcher[28]	CFS	2	2	1	2	1	Low
2005	Gallagher[29]	CFS	1	2	1	1	1	Low
2003	Georgiades[30]	CFS	2	2	3	2	2	Low
2016	Gerdle[31]	FMS	1	1	1	2	1	Low
2013	Gerdle[32]	FMS	2	2	3	3	3	High
2008	Giske[33]	FMS	2	2	3	2	2	Low
2012	Goes[34]	FMS	2	1	1	1	1	Low
2016	Goes[35]	FMS	1	1	1	2	1	Low
2015	Gomez -Cabello[36]	FMS	2	2	3	3	3	High
2020	Hodges[37]	CFS	2	1	2	3	3	High
2010	Hsieh[38]	FMS	1	2	3	3	3	High
2013	Ickmans[39]	CFS	2	3	3	1	3	High

2014	Icksman[40]	FMS	1	2	3	1	2	Low
2001	Inbar[41]	CFS	2	2	3	2	2	Low
2005	Jammes[42]	CFS	2	2	3	2	2	Low
2007	Javierre[43]	CFS	3	2	3	3	3	High
2021	Jäkel [44]	CFS	2	2	2	2	2	Low
2022	Kapuczinski [45]	FMS	3	3	2	3	3	High
2012	Klaver - Krol[46]	FMS	2	2	3	2	2	Low
2015	Koklu[47]	FMS	2	1	3	3	3	High
2015	Latorre Roman[48]	FMS	2	2	3	3	3	High
2018	Larsson[49]	FMS	2	2	3	3	3	High
2014	Lee[50]	FMS	2	2	3	3	3	High
1991	Lloyd[51]	CFS	2	2	1	2	1	Low
2006	Lowe[52]	FMS	2	1	3	1	2	Low
2003	Lund[53]	FMS	2	2	3	2	2	Low
2010	Maquet[54]	CFS/FMS	2	2	3	3	3	High
2002	Maquet[55]	FMS	2	2	3	3	3	High
2005	Maquet[56]	FMS	3	2	3	3	3	High
1990	Mengshoel[57]	FMS	3	2	3	3	3	High
1995	Mengshoel[58]	FMS	2	2	3	2	2	Low
2018	Nacul[59]	CFS	2	2	3	3	3	High
2019	Nelson[60]	CFS	1	1	3	1	1	Low
2010	Nijs[61]	CFS	1	2	3	2	2	Low

1993	Nordenskiöld[62]	FMS	3	2	3	3	3	High
1994	Nørregaard[63]	FMS	2	3	3	2	3	High
1995	Nørregaard [64]	FMS	3	1	3	2	3	High
2017	Van Oosterwijck[65]	CFS	1	2	2	1	1	Low
2021	Van Oosterwijck [66]	CFS	3	2	2	3	3	High
2017	Paiva[67]	FMS	2	2	3	3	3	High
2006	Panton[68]	FMS	2	2	3	2	2	Low
2008	Patrick -Neary[69]	CFS	1	2	3	2	2	Low
1999	Paul [70]	CFS	1	2	3	2	2	Low
2017	Pieroni-Andrade[71]	FMS	2	2	3	3	3	High
1990	Riley[72]	CFS	2	2	3	3	3	High
1998	Rowbottom[73]	CFS	2	2	3	2	2	Low
1999	Sacco[74]	CFS	2	1	3	2	2	Low
2004	Sahin[75]	FMS	2	2	3	2	2	Low
2020	Salaffi[76]	FMS	2	2	3	3	3	High
2002	Sargent[77]	CFS	1	1	1	1	1	Low
2015	Segura[78]	FMS	2	2	3	3	3	High
2019	Sempere-Rubio [79]	FMS	2	2	3	3	3	High
2016	Sener[80]	FMS	2	2	3	2	2	Low
2004	Siemionow[81]	CFS	2	1	3	2	2	Low
1994	Simms[82]	FMS	2	2	3	2	2	Low
1996	Sisto[83]	CFS	1	1	3	1	2	Low

2013	Srikuea[84]	FMS	2	2	3	2	2	Low
2005	Staud[85]	FMS	2	2	3	3	3	High
2013	Strahler[86]	CFS	2	3	3	2	3	High
2010	Suarez[87]	CFS	2	3	3	2	3	High
2020	Tavares[88]	FMS	1	2	3	2	2	Low
2015	Umeda[89]	FMS	2	1	2	2	2	Low
2003	Valim[90]	FMS	2	2	2	2	1	Low
2008	Valkeinen[91]	FMS	2	2	3	3	3	High
1992	Van Denderen[92]	FMS	2	3	3	3	3	High
2007	Van Ness[93]	CFS	2	3	3	3	3	High
2010	Vermeulen[94]	CFS	1	2	2	2	1	Low
2014	Vermeulen[95]	CFS	3	1	3	2	2	Low
2018	Villafaina[96]	FMS	2	2	3	3	3	High
2016	Vincent[97]	FMS	2	3	2	3	3	High
2022	Wåhlén [98]	FMS	2	2	1	2	2	Low
2004	Wallman[99]	CFS	2	2	3	1	2	Low

Table 1. Risk of Bias Table. Low = Low risk of Bias; High = High risk of Bias.

References:

1. Aerenhouts D, Ickmans K, Clarys P, et al. Sleep characteristics, exercise capacity and physical activity in patients with chronic fatigue syndrome. *Disability and rehabilitation.* 2015;37(22):2044-2050.
2. Acosta-Manzano P, Segura-Jimenez V, Estevez-Lopez F, et al. Do women with fibromyalgia present higher cardiovascular disease risk profile than healthy women? The al-Andalus project. *Clin Exp Rheumatol.* 2017;35 Suppl 105(3):61-67.
3. Andretta A, Schieferdecker MEM, Petterle RR, Dos Santos Paiva E, Boguszewski CL. Relations between serum magnesium and calcium levels and body composition and metabolic parameters in women with fibromyalgia. *Advances in rheumatology (London, England).* 2020;60(1):18.
4. Aparicio VA, Carbonell-Baeza A, Ortega FB, Ruiz JR, Heredia JM, Delgado-Fernandez M. Handgrip strength in men with fibromyalgia. *Clin Exp Rheumatol.* 2010;28(6 Suppl 63):S78-81.
5. Aparicio VA, Ortega FB, Carbonell-Baeza A, Camiletti D, Ruiz JR, Delgado-Fernandez M. Relationship of weight status with mental and physical health in female fibromyalgia patients. *Obes Facts.* 2011;4(6):443-448.
6. Aparicio VA, Carbonell-Baeza A, Ruiz JR, et al. Fitness testing as a discriminative tool for the diagnosis and monitoring of fibromyalgia. *Scandinavian journal of medicine & science in sports.* 2013;23(4):415-423.
7. Ariadna Aparicio V, Segura-JimÉNez V, ÁLvarez-Gallardo IC, et al. Fitness Testing in the Fibromyalgia Diagnosis: The al-Ándalus Project. *Medicine & Science in Sports & Exercise.* 2015;47(3):451-459.
8. Bachasson D, Guinot M, Wuyam B, et al. Neuromuscular fatigue and exercise capacity in fibromyalgia syndrome. *Arthritis Care Res (Hoboken).* 2013;65(3):432-440.
9. Balbaloglu O, Ede H, Yolcu S, Ak H, Tanik N, Tekin G. Exercise Profile and Diastolic Functions Measured via Tissue Doppler Imaging of Fibromyalgia Patients. *J Clin Med Res.* 2014;6(3):184-189.
10. Bardal EM, Olsen TV, Ettema G, Mork PJ. Metabolic rate, cardiac response, and aerobic capacity in fibromyalgia: a case-control study. *Scand J Rheumatol.* 2013;42(5):417-420.
11. Bardal EM, Roeleveld K, Mork PJ. Aerobic and cardiovascular autonomic adaptations to moderate intensity endurance exercise in patients with fibromyalgia. *J Rehabil Med.* 2015;47(7):639-646.
12. Bazelmans E, Bleijenberg G, Van Der Meer JW, Folgering H. Is physical deconditioning a perpetuating factor in chronic fatigue syndrome? A controlled study on maximal exercise performance and relations with fatigue, impairment and physical activity. *Psychol Med.* 2001;31(1):107-114.
13. Blackwood SK, MacHale SM, Power MJ, Goodwin GM, Lawrie SM. Effects of exercise on cognitive and motor function in chronic fatigue syndrome and depression. *J Neurol Neurosurg Psychiatry.* 1998;65(4):541-546.
14. Borman P, Celiker R, Hascelik Z. Muscle performance in fibromyalgia syndrome. *Rheumatol Int.* 1999;19(1-2):27-30.
15. Berardi G, Senefeld JW, Hunter SK, Bement MKH. Impact of isometric and concentric resistance exercise on pain and fatigue in fibromyalgia. *Eur J Appl Physiol.* 2021;121(5):1389-1404.
16. Bouquet J, Li T, Gardy JL, et al. Whole blood human transcriptome and virome analysis of ME/CFS patients experiencing post-exertional malaise following cardiopulmonary exercise testing. *PLoS One.* 2019;14(3):e0212193.
17. Cerón-Lorente L, Valenza MC, Pérez-Mármol JM, García-Ríos MdC, Castro-Sánchez AM, Aguilar-Ferrándiz ME. The influence of balance, physical disability, strength, mechanosensitivity and spinal mobility on physical activity at home, work and leisure time in women with fibromyalgia. *Clinical Biomechanics.* 2018;60:157-163.

18. Cook DB, Nagelkirk PR, Peckerman A, Poluri A, Lamanca JJ, Natelson BH. Perceived exertion in fatiguing illness: civilians with chronic fatigue syndrome. *Med Sci Sports Exerc.* 2003;35(4):563-568.
19. Cook DB, Nagelkirk PR, Poluri A, Mores J, Natelson BH. The influence of aerobic fitness and fibromyalgia on cardiorespiratory and perceptual responses to exercise in patients with chronic fatigue syndrome. *Arthritis and rheumatism.* 2006;54(10):3351-3362.
20. Cook DB, Stegner AJ, Nagelkirk PR, Meyer JD, Togo F, Natelson BH. Responses to exercise differ for chronic fatigue syndrome patients with fibromyalgia. *Medicine and science in sports and exercise.* 2012;44(6):1186-1193.
21. Cook DB, VanRiper S, Dougherty RJ, et al. Cardiopulmonary, metabolic, and perceptual responses during exercise in Myalgic Encephalomyelitis/Chronic Fatigue Syndrome (ME/CFS): A Multi-site Clinical Assessment of ME/CFS (MCAM) sub-study. *PloS one.* 2022;17(3):e0265315.
22. da Cunha Ribeiro RP, Roschel H, Artioli GG, et al. Cardiac autonomic impairment and chronotropic incompetence in fibromyalgia. *Arthritis Res Ther.* 2011;13(6):R190.
23. De Becker P, Roeykens J, Reynders M, McGregor N, De Meirleir K. Exercise capacity in chronic fatigue syndrome. *Arch Intern Med.* 2000;160(21):3270-3277.
24. Dinler M, Kasikcioglu E, Akin A, et al. Exercise capacity and oxygen recovery half times of skeletal muscle in patients with fibromyalgia. *Rheumatol Int.* 2007;27(3):311-313.
25. Dinler M, Diracoglu D, Kasikcioglu E, et al. Effect of aerobic exercise training on oxygen uptake and kinetics in patients with fibromyalgia. *Rheumatol Int.* 2009;30(2):281-284.
26. Elert J, Kendall SA, Larsson B, Mansson B, Gerdle B. Chronic pain and difficulty in relaxing postural muscles in patients with fibromyalgia and chronic whiplash associated disorders. *J Rheumatol.* 2001;28(6):1361-1368.
27. Farquhar WB, Hunt BE, Taylor JA, Darling SE, Freeman R. Blood volume and its relation to peak O(2) consumption and physical activity in patients with chronic fatigue. *Am J Physiol Heart Circ Physiol.* 2002;282(1):H66-71.
28. Fulcher KY, White PD. Strength and physiological response to exercise in patients with chronic fatigue syndrome. *J Neurol Neurosurg Psychiatry.* 2000;69(3):302-307.
29. Gallagher AM, Coldrick AR, Hedge B, Weir WRC, White PD. Is the chronic fatigue syndrome an exercise phobia? A case control study. *Journal of psychosomatic research.* 2005;58(4):367-373.
30. Georgiades E, Behan WM, Kilduff LP, et al. Chronic fatigue syndrome: new evidence for a central fatigue disorder. *Clin Sci (Lond).* 2003;105(2):213-218.
31. Gerdle B, Ernberg M, Mannerkorpi K, et al. Increased Interstitial Concentrations of Glutamate and Pyruvate in Vastus Lateralis of Women with Fibromyalgia Syndrome Are Normalized after an Exercise Intervention - A Case-Control Study. *PLoS One.* 2016;11(10):e0162010.
32. Gerdle B, Forsgren MF, Bengtsson A, et al. Decreased muscle concentrations of ATP and PCR in the quadriceps muscle of fibromyalgia patients--a 31P-MRS study. *European journal of pain (London, England).* 2013;17(8):1205-1215.
33. Giske L, Vøllestad NK, Mengshoel AM, Jensen J, Knardahl S, Røe C. Attenuated adrenergic responses to exercise in women with fibromyalgia--a controlled study. *European Journal of Pain.* 2008;12(3):351-360.
34. Góes SM, Leite N, Shay BL, Homann D, Stefanello JMF, Rodacki ALF. Functional capacity, muscle strength and falls in women with fibromyalgia. *Clinical biomechanics (Bristol, Avon).* 2012;27(6):578-583.
35. Góes SM, Stefanello JMF, Homann D, Lodovico A, Hubley-Kozey CL, Rodacki ALF. Torque and Muscle Activation Impairment Along With Insulin Resistance Are Associated With Falls in Women With Fibromyalgia. *Journal of strength and conditioning research.* 2016;30(11):3155-3164.
36. Gómez-Cabello A, Vicente-Rodríguez G, Navarro-Vera I, Martinez-Redondo D, Díez-Sánchez C, Casajús JA. Influences of physical fitness on bone mass in women with fibromyalgia. *Adapted physical activity quarterly : APAQ.* 2015;32(2):125-136.

37. Hodges L, Nielsen T, Cochrane D, Baken D. The physiological time line of post-exertional malaise in Myalgic Encephalomyelitis/Chronic Fatigue Syndrome (ME/CFS). *Translational sports medicine.* 2020.
38. Hsieh LF, Chien HL, Chuang CC, Bai CH. Aerobic capacity is reduced in Chinese women with primary fibromyalgia syndrome. *J Musculoskel Pain.* 2010;18(3):216-225.
39. Ickmans K, Clarys P, Nijs J, et al. Association between cognitive performance, physical fitness, and physical activity level in women with chronic fatigue syndrome. *Journal of rehabilitation research and development.* 2013;50(6):795-810.
40. Ickmans K, Meeus M, De Kooning M, Lambrecht L, Pattyn N, Nijs J. Can Recovery of Peripheral Muscle Function Predict Cognitive Task Performance in Chronic Fatigue Syndrome With and Without Fibromyalgia? *Physical Therapy.* 2014;94(4):511-522.
41. Inbar O, Dlin R, Rotstein A, Whipp BJ. Physiological responses to incremental exercise in patients with chronic fatigue syndrome. *Med Sci Sports Exerc.* 2001;33(9):1463-1470.
42. Jammes Y, Steinberg JG, Mambrini O, Bregeon F, Delliaux S. Chronic fatigue syndrome: assessment of increased oxidative stress and altered muscle excitability in response to incremental exercise. *J Intern Med.* 2005;257(3):299-310.
43. Javierre C, Alegre J, Ventura JL, et al. Physiological responses to arm and leg exercise in women patients with chronic fatigue syndrome. *Journal of Chronic Fatigue Syndrome.* 2007;14(1):43-53.
44. Jäkel B, Kedor C, Grabowski P, et al. Hand grip strength and fatigability: correlation with clinical parameters and diagnostic suitability in ME/CFS. *J Transl Med.* 2021;19(1):159.
45. Kapuczinski A, Soyfoo MS, De Breucker S, Margaux J. Assessment of sarcopenia in patients with fibromyalgia. *Rheumatology international.* 2022;42(2):279-284.
46. Klaver-Król EG, Rasker JJ, Henriquez NR, Verheijen WG, Zwarts MJ. Muscle fiber velocity and electromyographic signs of fatigue in fibromyalgia. *Muscle & Nerve.* 2012;46(5):738-745.
47. Köklü K, Sarigül M, Özişler Z, Şirzai H, Özel S. Handgrip Strength in Fibromyalgia. *Archives of rheumatology.* 2015;31(2):158-161.
48. Latorre Román P, Santos ECMA, García-Pinillos F. Effects of functional training on pain, leg strength, and balance in women with fibromyalgia. *Modern rheumatology.* 2015;25(6):943-947.
49. Larsson A, Palstam A, Bjersing J, et al. Controlled, cross-sectional, multi-center study of physical capacity and associated factors in women with fibromyalgia. *BMC Musculoskelet Disord.* 2018;19(1):121.
50. Lee SJ, Ahn DH, Jung JH, Kim YR, Lee YJ. Short-term change of handgrip strength after trigger point injection in women with muscular pain in the upper extremities. *Annals of rehabilitation medicine.* 2014;38(2):241-248.
51. Lloyd AR, Gandevia SC, Hales JP. Muscle performance, voluntary activation, twitch properties and perceived effort in normal subjects and patients with the chronic fatigue syndrome. *Brain.* 1991;114 (Pt 1A):85-98.
52. Lowe JC, Yellin J, Honeyman-Lowe G. Female fibromyalgia patients: lower resting metabolic rates than matched healthy controls. *Med Sci Monit.* 2006;12(7):Cr282-289.
53. Lund E, Kendall SA, Janerot-Sjoberg B, Bengtsson A. Muscle metabolism in fibromyalgia studied by P-31 magnetic resonance spectroscopy during aerobic and anaerobic exercise. *Scand J Rheumatol.* 2003;32(3):138-145.
54. Maquet D, Croisier JL, Dupont C, et al. Fibromyalgia and related conditions: electromyogram profile during isometric muscle contraction. *Joint Bone Spine.* 2010;77(3):264-267.
55. Maquet D, Croisier J-L, Renard C, Crielaard J-M. Muscle performance in patients with fibromyalgia. *Joint bone spine.* 2002;69(3):293-299.
56. Macquet D, Demoulin C, Forthomme B, Crielaard JM, Croisier JL. Activation EMG pattern during isokinetic knee flexion-extension assessment: comparison between healthy subjects and chronic pain patients. *Isokinetics & Exercise Science.* 2005;13(1):5-11.

57. Mengshoel AM, Forre O, Komnaes HB. Muscle strength and aerobic capacity in primary fibromyalgia. *Clin Exp Rheumatol.* 1990;8(5):475-479.
58. Mengshoel AM, Saugen E, Forre O, Vøllestad NK. Muscle fatigue in early fibromyalgia. *The Journal of rheumatology.* 1995;22(1):143-150.
59. Nacul LC, Mudie K, Kingdon CC, Clark TG, Lacerda EM. Hand Grip Strength as a Clinical Biomarker for ME/CFS and Disease Severity. *Frontiers in neurology.* 2018;9:992.
60. Nelson MJ, Bahl JS, Buckley JD, Thomson RL, Davison K. Evidence of altered cardiac autonomic regulation in myalgic encephalomyelitis/chronic fatigue syndrome: A systematic review and meta-analysis. *Medicine (Baltimore).* 2019;98(43):e17600.
61. Nijs J, Van Oosterwijck J, Meeus M, et al. Unravelling the nature of postexertional malaise in myalgic encephalomyelitis/chronic fatigue syndrome: the role of elastase, complement C4a and interleukin-1beta. *J Intern Med.* 2010;267(4):418-435.
62. Nordenskiold UM, Grimby G. Grip force in patients with rheumatoid arthritis and fibromyalgia and in healthy subjects. A study with the Grippit instrument. *Scand J Rheumatol.* 1993;22(1):14-19.
63. Nørregaard J, Bülow PM, Mehlsen J, Danneskiold-Samsøe B. Biochemical changes in relation to a maximal exercise test in patients with fibromyalgia. *Clinical physiology (Oxford, England).* 1994;14(2):159-167.
64. Nørregaard J, Volkmann H, Danneskiold-Samsøe B. A randomized controlled trial of citalopram in the treatment of fibromyalgia. *Pain.* 1995;61(3):445-449.
65. Oosterwijck JV, Marusic U, De Wandele I, et al. The Role of Autonomic Function in Exercise-induced Endogenous Analgesia: A Case-control Study in Myalgic Encephalomyelitis/Chronic Fatigue Syndrome and Healthy People. *Pain physician.* 2017;20(3):E389-E399.
66. Van Oosterwijck J, Marusic U, De Wandele I, et al. Reduced Parasympathetic Reactivation during Recovery from Exercise in Myalgic Encephalomyelitis/Chronic Fatigue Syndrome. *J Clin Med.* 2021;10(19).
67. Paiva ES, Andretta A, Batista ED, et al. Serum levels of leptin and adiponectin and clinical parameters in women with fibromyalgia and overweight/obesity. *Arch Endocrinol Metab.* 2017;61(3):249-256.
68. Panton LB, Kingsley JD, Toole T, et al. A comparison of physical functional performance and strength in women with fibromyalgia, age- and weight-matched controls, and older women who are healthy. *Phys Ther.* 2006;86(11):1479-1488.
69. Patrick Neary J, Roberts AD, Leavins N, Harrison MF, Croll JC, Sexsmith JR. Prefrontal cortex oxygenation during incremental exercise in chronic fatigue syndrome. *Clin Physiol Funct Imaging.* 2008;28(6):364-372.
70. Paul L, Wood L, Behan WM, Maclaren WM. Demonstration of delayed recovery from fatiguing exercise in chronic fatigue syndrome. *Eur J Neurol.* 1999;6(1):63-69.
71. Andrade CP, Zamunér AR, Forti M, et al. The Borg CR-10 scale is suitable to quantify aerobic exercise intensity in women with fibromyalgia syndrome. *Fisioterapia e Pesquisa.* 2017;24:267-272.
72. Riley MS, O'Brien CJ, McCluskey DR, Bell NP, Nicholls DP. Aerobic work capacity in patients with chronic fatigue syndrome. *Bmj.* 1990;301(6758):953-956.
73. Rowbottom DG, Keast D, Green S, Kakulas B, Morton AR. The case history of an elite ultra-endurance cyclist who developed chronic fatigue syndrome. *Med Sci Sports Exerc.* 1998;30(9):1345-1348.
74. Sacco P, Hope PA, Thickbroom GW, Byrnes ML, Mastaglia FL. Corticomotor excitability and perception of effort during sustained exercise in the chronic fatigue syndrome. *Clin Neurophysiol.* 1999;110(11):1883-1891.
75. Sahin G, Ulubas B, Calikoglu M, Bolgen Cime O, Erdogan C. Is chest expansion a determinant of pulmonary muscle strength in primary fibromyalgia? *Joint Bone Spine.* 2004;71(2):140-143.
76. Salaffi F, Farah S, Di Carlo M. Force-time curve features of handgrip strength in fibromyalgia syndrome. *Scientific reports.* 2020;10(1):3372.
77. Sargent C, Scroop GC, Nemeth PM, Burnet RB, Buckley JD. Maximal oxygen uptake and lactate metabolism are normal in chronic fatigue syndrome. *Med Sci Sports Exerc.* 2002;34(1):51-56.

78. Segura-Jimenez V, Aparicio VA, Alvarez-Gallardo IC, Carbonell-Baeza A, Tornero-Quinones I, Delgado-Fernandez M. Does body composition differ between fibromyalgia patients and controls? the al-Ándalus project. *Clinical and experimental rheumatology.* 2015;33(1 Suppl 88):S25-S32.
79. Sempere-Rubio N, Aguilar-Rodriguez M, Ingles M, Izquierdo-Alventosa R, Serra-Ano P. Physical Condition Factors that Predict a Better Quality of Life in Women with Fibromyalgia. *Int J Environ Res Public Health.* 2019;16(17).
80. Sener U, Ucok K, Ulasli AM, et al. Evaluation of health-related physical fitness parameters and association analysis with depression, anxiety, and quality of life in patients with fibromyalgia. *Int J Rheum Dis.* 2016;19(8):763-772.
81. Siemionow V, Fang Y, Calabrese L, Sahgal V, Yue GH. Altered central nervous system signal during motor performance in chronic fatigue syndrome. *Clinical neurophysiology : official journal of the International Federation of Clinical Neurophysiology.* 2004;115(10):2372-2381.
82. Simms RW, Roy SH, Hrovat M, et al. Lack of association between fibromyalgia syndrome and abnormalities in muscle energy metabolism. *Arthritis Rheum.* 1994;37(6):794-800.
83. Sisto SA, LaManca J, Cordero DL, et al. Metabolic and cardiovascular effects of a progressive exercise test in patients with chronic fatigue syndrome. *Am J Med.* 1996;100(6):634-640.
84. Srikuea R, Symons TB, Long DE, et al. Association of fibromyalgia with altered skeletal muscle characteristics which may contribute to postexertional fatigue in postmenopausal women. *Arthritis Rheum.* 2013;65(2):519-528.
85. Staud R, Vierck CJ, Robinson ME, Price DD. Effects of the N-methyl-D-aspartate receptor antagonist dextromethorphan on temporal summation of pain are similar in fibromyalgia patients and normal control subjects. *J Pain.* 2005;6(5):323-332.
86. Strahler J, Fischer S, Nater UM, Ehlert U, Gaab J. Norepinephrine and epinephrine responses to physiological and pharmacological stimulation in chronic fatigue syndrome. *Biol Psychol.* 2013;94(1):160-166.
87. Suarez A, Guillamo E, Roig T, et al. Nitric oxide metabolite production during exercise in chronic fatigue syndrome: a case-control study. *J Womens Health (Larchmt).* 2010;19(6):1073-1077.
88. Tavares LF, Germano Maciel D, Pereira Barros da Silva TY, Brito Vieira WHd. Comparison of functional and isokinetic performance between healthy women and women with fibromyalgia. *Journal of bodywork and movement therapies.* 2020;24(1):248-252.
89. Umeda M, Corbin LW, Maluf KS. Examination of contraction-induced muscle pain as a behavioral correlate of physical activity in women with and without fibromyalgia. *Disabil Rehabil.* 2015;37(20):1864-1869.
90. Valim V, Oliveira L, Suda A, et al. Aerobic fitness effects in fibromyalgia. *J Rheumatol.* 2003;30(5):1060-1069.
91. Valkeinen H, Alen M, Hakkinen A, Hannonen P, Kukkonen-Harjula K, Hakkinen K. Effects of concurrent strength and endurance training on physical fitness and symptoms in postmenopausal women with fibromyalgia: a randomized controlled trial. *Arch Phys Med Rehabil.* 2008;89(9):1660-1666.
92. van Denderen JC, Boersma JW, Zeinstra P, Hollander AP, van Neerbos BR. Physiological effects of exhaustive physical exercise in primary fibromyalgia syndrome (PFS): is PFS a disorder of neuroendocrine reactivity? *Scand J Rheumatol.* 1992;21(1):35-37.
93. Van Ness JM, Snell CR, Stevens SR. Diminished Cardiopulmonary Capacity During Post-Exertional Malaise. *Journal of Chronic Fatigue Syndrome.* 2007;14(2):77-85.
94. Vermeulen RC, Kurk RM, Visser FC, Sluiter W, Scholte HR. Patients with chronic fatigue syndrome performed worse than controls in a controlled repeated exercise study despite a normal oxidative phosphorylation capacity. *J Transl Med.* 2010;8:93.
95. Vermeulen RC, Vermeulen van Eck IW. Decreased oxygen extraction during cardiopulmonary exercise test in patients with chronic fatigue syndrome. *J Transl Med.* 2014;12:20.
96. Villafaina S, Collado-Mateo D, Domínguez-Muñoz FJ, Fuentes-García JP, Gusi N. Impact of adding a cognitive task while performing physical fitness tests in women with fibromyalgia: A cross-sectional descriptive study. *Medicine.* 2018;97(51):e13791.

97. Vincent A, Whipple MO, Low PA, Joyner M, Hoskin TL. Patients With Fibromyalgia Have Significant Autonomic Symptoms But Modest Autonomic Dysfunction. *PM & R : the journal of injury, function, and rehabilitation.* 2016;8(5):425-435.
98. WÅHlÉN K, Yan H, Welinder C, et al. Proteomic Investigation in Plasma from Women with Fibromyalgia in Response to a 15-wk Resistance Exercise Intervention. *Medicine & Science in Sports & Exercise.* 2022;54(2):1-15.
99. Wallman KE, Morton AR, Goodman C, Grove R. Physiological responses during a submaximal cycle test in chronic fatigue syndrome. *Medicine and science in sports and exercise.* 2004;36(10):1682-1688.
