# Supplementary material for: Fibromyalgia and Chronic Fatigue Syndromes: A systematic review and meta-analysis of cardiorespiratory fitness and neuromuscular function compared with healthy individuals
Source: PLoS One. 2022 Oct 20;17(10):e0276009. doi: 10.1371/journal.pone.0276009 (PMC9584387; doi:10.1371/journal.pone.0276009)
Supplement: S2 File — Descriptive tables of all the studies included in the systematic review and meta-analysis. (PDF) [file pone.0276009.s002.pdf]

Table 2. Study characteristics for the CPET studies included in the Systematic Review and Meta-analysis for chronic fatigue syndrome.

Year	Name	Type	Diagn.	test	type	Testing protocol	RPE	HR	AT	Lactate
2015	*Aerenhouts*	*CFS*	*CDC*	*VE*	*C*	*Cycling, test started at 60w with 30w increased every minute until exhaustion or participant pedalling rate sunk below 55rpm*				
2001	*Bazelmans*	*CFS*	*CDC*	*VE*	*C*	*Cycling: Workload was increased every minute in step of 10% of estimated maximal workload, from 10 to 30%*	*At 3min*	*Peak*		
1998	*Blackwood*	*CFS*	*Not specified*	*VE*	*T*	*Treadmill: Standardize Bruce Protocol*	*At 85% HRmax*			
2019	Bouquet	CFS	Canadian 2003	VE	C	Cycling: Workload was increased 15 watts per minute until volitional fatigue.		Peak	VT	
2003	Cook	CFS	CDC	M	T	Treadmill: exercise stage was 3 min, and the exercise test began at 67 m·min1 and no incline. For stage 2 of exercise, the treadmill speed was increased to 94 m·min1. For the remaining stages, speed was kept constant, and intensity was increased by raising the incline of the treadmill by 2% at each stage until the end of the test.	At GET	Peak	GET	
2006	Cook	CFS	CDC	M	C	Cycling: increased by 5W every 20 seconds	mean	Peak		
2012	Cook	CFS	CDC	S	C	Cycling: Constant Intensity at 40% VO2peak for 20minutes	mean	Mean		
2022	Cook	CFS	Not known	VE	C	3min unloaded warm-up. Exercise testing from 0 Watts with linear increases of 5 Watts every 20 seconds (15 Watts/min) until volitional exhaustion at 60-70rpm.	Mean	Peak		Peak
2000	De Becker	CFS	CDC	S/M	C	Cycling: starting at 10w and increasing 10w each minute		Peak		
2002	Farquhar	CFS	CDC	VE	C	Cycling: 2 min stages, workload increase of 25-30watts		Peak		
2000	Fulcher	CFS	Oxford	VE	T	Treadmill: 5kph with 2.5% of gradient increase every 2minutes		Peak		

2005	Gallagher	CFS	Oxford	VE	T	Treadmill: 5kph with 2.5% of gradient increase every 2minutes		Peak		
2003	Georgiades	CFS	CDC	VE	C	Cycling: incrementation rate for individual subjects varied between 3 and 10w/min		Peak		Peak
2020	Hodges	CFS	ICC	VE/M	C	Cycling: Starting at 15w and increase 15w/min	At VT	Peak	RER	
2013	Icksman	CFS	CDC	VE	C	Cycling: start from 60w and increase by 30w every minute		Peak		
2001	Inbar	CFS	CDC	M	T	Treadmill: Modified balke protocol - constant speed of 2.0-3.5mph while the slope is elevated by 2% every minute		Peak	GET	
2005	Jammes	CFS	symptoms for CFS	VE	C	Cycling: workload started at 20W and increased every minute of 20w until subjects stop pedalling			VT	
2007	Javierre	CFS	CDC	VE	C	Cycling: increase of 12.5 watt every one minute until exhaustion	Peak			
2019	Nelson	CFS	CDC	M	C	Cycling: 5minutes at 40w (male) and 30w (female) and then 5w increased every 20sec until exhaustion	at VT	Peak	VT	
2010	Nijs	CFS	CDC	S	C	Cycling: Submaximal Aerobic Power Index test				Peak
2017	Van Oosterwijck	CFS	CDC	S	C	Cycling: Submaximal Aerobic Power Index test		Peak		
2008	Neary	CFS	CDC	I	C	Cycling: at 60 W for a period of 2 min, followed by a work rate increase of 25 W every 2 min until exhaustion		Peak		
1990	Riley	CFS	CDC	VE	T	Treadmill: Modified Bruce Protocol	Peak	Peak		Peak
1998	Rowbottom	CFS	Komaroff	VE	T	Treadmill: Modified Bruce protocol	mean	Peak		
2002	Sargent	CFS	CDC	M	C	Cycling: 25w every 2 minute until the subject was not able to maintain the power output		Peak	LT	Peak
1996	Sisto	CFS	CDC	M	T	Treadmill: 3.5mph with the decline increase of 2% every minute	at GET	Peak	GET	
2013	Strahler	CFS	CDC	VE	C	Cycling: Started at 50 W for men and 30 W for women, respectively, with 40 W increments every 3 min until the subject was no longer able to continue or until predicted maximum heart rate (85% of 220bpm)	Peak	Peak		
2010	Suarez	CFS	CDC	VE	C	Cycling: 20w every minute until exhaustion	Peak	Peak		Peak
2007	Van Ness	CFS	CDC	VE	C/T	Cycling or treadmill: Bruce treadmill or 10w/min ramping protocol			AT (not specified)	
2021	Van Oosterwijck	CFS	CDCP	VE	C	Cycling: Submaximal Aerobic Power Index test	Peak	Peak		

2010	Vermeulen	CFS	CDC	VE	C	Cycling: RAMP protocol based on gender and history of physical examination, Weight and height		Peak	AT (not specified)
2014	Vermeulen	CFS	CDC	VE	C	Cycling: RAMP protocol based on gender and history of physical examination, Weight and height		Peak	AT (not specified)
2004	Wallman	CFS	CDC	S	C	Cycling: Aerobic Power Index test - Increased of 25w every minute until reaching 75%of age predicted target HR	At peak workload		3 min after the test

Abbreviations: FMS = Fibromyalgia Syndrome, ACR = American College of Rheumatology, VE= Voluntary Exhaustion, M= Maximal, S = Submaximal, I = Indirect, C= Cycling, T = Treadmill, HG = Handgrip, RPE = Rate of Perceived Exertion, HR = Heart Rate, AT = Anaerobic Threshold, VT = Ventilatory Threshold, GET = Gas Exchange Threshold.

Table 3. Study characteristics for the CPET studies included in the Systematic Review and Meta-analysis for Fibromyalgia.

Year	*Name*	*Type*	*Diagn.*	*Test*	*Type*	*Testing protocol*	*RPE*	*HR*	*VT*	*Lactate*
2013	*Bachassons*	*FMS*	*ACR*	*VE*	*C*	*Cycling:15w initial power and increase of 15w/min for FMS and initial power at 30w increase of 30w/min for control group*	*At 75 or 50%*	*Peak*		*Peak*
2014	*Balbaloglu*	*FMS*	*ACR*	*VE*	*T*	*Treadmill: Modified Bruce Protocol*		*Peak*		
2013	*Bardal*	*FMS*	*ACR*	*VE*	*C*	*Cycling: Stepwise Increase of 15w/min*	*Peak*	*Peak*	*At 4mmol*	*Peak*
2015	Bardal	FMS	ACR	S	C	Cycling: a stepwise increase in workload (10 W/min) until blood lactate concentration (bLa) reached > 5 mmol/l.	mean	Mean		
2021	Berardi	FMS	Not Known	Strength	Elbow Flexors	Submaximal intermittent isometric and concentric muscle contractions matched for intensity (20% of maximal voluntary isometric contraction), duration (10-min), and duty-cycle (2-s contraction: 1-s relaxation)	mean			
2006	Cook	FMS	ACR 1990	M	C	Cycling: 3min warm up at 20w, then 5w increase every 20sec until exhaustion	mean	Peak		

2012	Cook	FMS	ACR 1990	S	C	Cycling: Constant Intensity at 40% VO2peak for 20minutes	mean	Mean		
2011	Da Cunha	FMS	ACR	VE	T	Treadmill: A modified Balke treadmill maximal exercise test		Peak		
2007	Dinler	FMS	ACR	VE	T	Treadmill: modified Bruce multistage protocol				
2009	Dinler	FMS	ACR	VE	T	Treadmill: Standard Bruce multistage protocol	Mean			
2012	Gerdle	FMS	Not Known	I	C	Cycling: Astrand Indirect Protocol				
2015	Gomez-Cabello	FMS	Not Known	VE	T	Treadmill: Fernhall protocol 1996				
2010	Hsieh	FMS	ACR	VE	C	Cycling: starting from 0 watt, adding increment of 10-15w/min	peak	Peak	VT	
2003	Lund	FMS	ACR 1990	VE	C	Cycling: Starting with two steady state submaximal levels of 6 min each [20 and 40 W 30 and 60 W], participants were exercised with continual workload increments of 10 – 20 W/min		Peak	VE/VO2	
1990	Mengshoel	FMS	Yunus	I	C	Cycling: Indirect and submaximal Astrand protocol				
1995	Mengshoel	FMS	ACR	I	C	Cycling: Indirect and submaximal Astrand protocol	at 6min			
1994	Norregard	FMS	ACR	VE	C	Cycling: initial load of 40 watts was used with stepwise increments every 3 min using the following steps, 70, 100,130,150,170,190 W until exhaustion.	At 6 min		At 2mmol (W)	Peak
2017	Pieroni - Andrade	FMS	ACR	VE	C	Cycling: Incremental Protocol, with increment proposed by Wasserman based on age, weight, and height		Peak	VT (ml/kg/min)	
2016	Sener	FMS	ACR	I	C	Cycling: Indirect and submaximal Astrand protocol				

1994	Simms	FMS	ACR	VE	C	Cycling: 1-minute stages of 15W increases, beginning at 0 W.	at peak	Peak		
2013	Srikuea	FMS	ACR	strength	Knee Extensor	6 sets of 12 isometric contractions with each set followed by MVIC as described above. Incremental intensity from 20% to 70%.	mean			
2005	Staud	FMS	ACR	Strength	HG	Sustained handgrip exercise at 30% of MVC for 90sec	End			
2002	Valim	FMS	ACR	VE	T	Treadmill: a 3 km/h load, with a 1 km/h increase every minute up to 7 km/h, after that, a 2.5% slope inclination increase up to 15%.		Peak	AT (%)	
2008	Valkeinen	FMS	ACR	VE	C	Cycling: 3min at 50w initial load, then 20w every 2min increases		Peak		Peak
1992	Van Denderen	FMS	Yunus	VE	C	Cycling: Starting with 5O Watts. the workload was raised every three minutes by 30 Watts		Peak		
2016	Vincent	FMS	ACR	VE	C	Cycling: Incremental until reaching VO2max or patients too fatigued (not specified the increment)		Peak		

Abbreviations: FMS = Fibromyalgia Syndrome, ACR = American College of Rheumatology, VE= Voluntary Exhaustion, M= Maximal, S = Submaximal, I = Indirect, C= Cycling, T = Treadmill, HG = Handgrip, RPE = Rate of Perceived Exertion, HR = Heart Rate, AT = Anaerobic Threshold, VT = Ventilatory Threshold, GET = Gas Exchange Threshold.

Table 4. Study characteristics for the Strength Assessment studies included in the Meta-analysis for Fibromyalgia and Chronic Fatigue Syndrome.

	Study Characteristics					Type	Type	VA	Fatigability
year	name	syndr.	Diagnosis	Sex	Matched	test	Muscle Action	type	type
2015	Aerenhouts	CFS	CDC - Fukuda	F	age - BMI	MVC	Handgrip		
1998	Blackwood	CFS	CDC- Fukuda	F/M	Age-sex	MVC	Handgrip		
2021	Berardi	Not Known	Physician	F	Age-Sex-BMI-PA	MVC	HG/elbow flexors	ITT	MVC decline
2000	Fulcher	CFS	CDC - Fukuda	F/M	Age sex-BMI	MVC	Leg extension	ITT (data not reported)	
2013	Icksman	CFS	CDC - Fukuda	F	Age sex and BMI	MVC	Handgrip		

2014	Icksman	FMS	CDC - Fukuda	F	Age sex and BMI	MVC	Handgrip		
1991	Lloyd	CFS	Lloyd et al 1988	F/M	age-BMI	MVC	Elbow flexion	ITT (endurance sequence test)	
2010	Maquet	CFS	CDC – Fukuda	F	Age sex and BMI	Submaximal Isometric Contraction	Arm Abduction		Time to exhaustion
2018	Nacul	CFS	CDC	-	None	MVC	Handgrip		
1999	Paul	CFS	CDC - Fukuda	F/M	age sex-BMI	MVC-Torque	Leg extension		fatigue index (%MVC)
1999	Sacco	CFS	CDC - Fukuda	F/M	age-sex-BM	MVC (N)	Elbow flexor		endurance time
2004	Siemionow	CFS	CDC - Fukuda	F/M	age-sex-BMI	MVC (N)	Handgrip		
2010	Aparicio	FMS	ACR	M	Age Sex BMI	Isometric MAX	Handgrip		
2011	Aparicio	FMS	ACR	F	Age Sex BMI	Isometric MAX	Handgrip		
2013	Aparicio	FMS	ACR	F	Age Sex BMI	Isometric MAX	Handgrip		
2013	Aparicio	FMS	ACR	F	Age Sex BMI	Isometric MAX	Handgrip		
2015	Aparicio	FMS	ACR	F	Age Sex BMI	Isometric MAX	Handgrip		
2013	Bachassons	FMS	ACR	F	Age Sex BMI	MVC	Leg extension		
1999	Borman	FMS	ACR	F	age and sex	isokinetic (PT)	Lex extension/flexors		Endurance ratio
2018	Ceron Lorente	FMS	ACR	?	Age – BMI	MVC	Leg extension		
2001	Elert	FMS	ACR	F	Age -Sex BMI	Isokinetic (PT)	Shoulder Abduction		
2012	Ge	FMS	ACR	F	Age – Sex - BMI		Shoulder Abduction		
2013	Gerdle	FMS	ACR	F	Sex- BMI	MVC and submaximal Isometric Contraction	Handgrip		Endurance/Time

2016	Gerdle	FMS	ACR	F	age and sex	MVC	Elbow Flexion		
2016	Gerdle	FMS	ACR	F	age and sex	MVC	Leg extension		
2012	Goes	FMS	ACR	F	Age-sex-BMI-PA	MVC - Torque	Leg extension		
2016	Goes	FMS	ACR	F	Age-sex-BMI	MVC	plantar flexion and dorsiflexion	CAR	
2008	Giske	FMS	ACR	F	age sex and weight	MVC	Quadriceps		
2021	Jäkel	CFS	CCC	F/M	Age-Sex	MVC	Quadriceps/HG		Fatigue Ratio
2015	Gomez-Cabello	FMS	ACR	F	Age -Sex	MVC	Leg extension and Handgrip		
2022	Kapuczinski	CFS	ACR	F	Sex	MVC	Handgrip		
2015	Koklu	FMS	ACR	F	Age-sex-BMI	MVC	Handgrip		
2018	Larsson	FMS	ACR	F	Age – sex	MVC	Leg extension/ Elbow flexion/ Handgrip		
2015	La Torre Roman	FMS	ACR	F/M	Age	MVC	Handgrip		
2014	Lee	FMS	ACR	F/M	BMI	MVC	Handgrip		
2012	Klaver – Kol	FMS	ACR	F/M	Age sex and BMI	MVC	Elbow flexor		
2002	Maquet	FMS	ACR	F	Age -Sex – BMI	MVC/ Isokinetic (PT)	Leg (extensors/flexors)		NCW
2005	Maquet	FMS	ACR	F	Age sex and BMI	Isokinetic (PT)	Leg (extensors/flexors)		NCW
2010	Maquet	FMS	ACR	F	Age – Sex – BMI	Submaximal Isometric contraction			Endurance/Time
1990	Mengshoel	FMS	Yunus	F	Age – Sex	MVC, Dynamic, Static (paper)	Handgrip		n. contraction in 30 sec/ time (s)
1993	Nordenskiöld	FMS	ACR	F	Sex Matched	Sustained MVC (10sec)	Handgrip		
1994	NØrregard	FMS	ACR	F	age-sex-BMI	MVC	Leg extension	VA	Endurance time (min)
1995	NØrregard	FMS	ACR	F	Age-sex-BMI	MVC	Leg extension	VA	
2006	Panton	FMS	Not mentioned	F	age-sex-BMI	MVC	Handgrip		
2004	Sahin	FMS	ACR	F	age sex-BMI	MVC	Handgrip		

2020	Salaffi	FMS	ACR	F	Age-sex-BMI	sustained MVC (30sec)	Handgrip		
2019	Sempere-Rubio	FMS	ACR	F	Age – Sex	MVC	Handgrip, Elbow flexion		
2016	Sener	FMS	ACR	F	age-sex-BMI	MVC	Handgrip		
1994	Simms	FMS	ACR	F	age-sex-BMI	MVC	Trapezius/Tibialis		
2013	Srikuea	FMS	ACR	F	Age-sex-BMI	MVC	Knee extensors	CAR	loss of strength
2020	Tavares	FMS	ACR	F	Age-sex-BMI	Isokinetic (PT)	Knee (extensor /flexor)		fatigue index
2015	Umeda	FMS	ACR	F	Age -sex	MVC	Handgrip		
2008	Valkeinen	FMS	ACR	F	Age-sex-BMI	MVC	Leg extension		
2018	Villafaina	FMS	ACR	F	Age – Sex -BMI	MVC	Handgrip		
2022	Wåhlén	FMS	ACR	F	Age – Sex	Maximal Tests	Leg extension/elbow flexors/HG		

Abbreviations: FMS = Fibromyalgia Syndrome, CFS = Chronic Fatigue Syndrome, ACR = American College of Rheumatology, F =Female, M= Male, BMI = Body Mass Index, ITT= Interpolated twitch technique, CAR = Central Activation Ratio, NCW = Normalized Cumulative Work – PA = Physical activity CCC- Canadian Consensus Criteria.

Table 5. Study characteristics for the Body Composition studies included in the Meta-analysis for Fibromyalgia and Chronic Fatigue Syndrome.

	Study Characteristics					Test	Muscle Volume	Muscle mass	Fat Mass
year	name	Syndrome	Diagnosis	Sex	Matched	type	Type	type	type
2017	Acosta-Manzano	FMS	ACR	F	Age	BIA			Fat Mass (%)
2020	Andretta	FMS	ACR	F	Age-BMI	DEXA		Lean Mass (%)	Fat Mass (%)
2013	Aparicio	FMS	ACR	F	Age -BMI	BIA		Muscle Mass (kg)	Fat Mass (%)
2015	Aparicio	FMS	ACR	F	Age – Sex -BMI	BIA		Muscle Mass (kg)	Fat Mass (%)
2013	Bachassons	FMS	ACR	F	Age – Sex -BMI	Skinfolds – Truncated cone calculation	Thigh		Fat Mass (%)
2021	Berardi	FMS	Not Known	F	Age- sex-BMI-PA				
2012	Gerdle	FMS	ACR	F	Age – Sex -BMI	MRI	Thigh		Subcutaneous Fat
2015	Gomez-Cabello	FMS	ACR	F	Age – Sex	DEXA		Lean Muscle Mass (kg)	Fat mass (kg)
2022	Kapuczinski	FMS	ACR	F	Sex	BIA		Lean Mass (kg)	Fat mass (kg)

2015	La Torre-Roman	FMS	ACR	F/M	Age	BIA		Muscle Mass (kg)	
2006	Lowe	FMS	ACR	F	Age-Sex-BMI	BIA		Fat Free Weight (kg)	Fat Mass (%)
1994	Norregard	FMS	ACR	F	Age-Sex-BMI	MRI		Fat free area (cm^2)	
1995	Norregard	FMS	ACR	F	Age-Sex-BMI	MRI	Sum-max Area (cm^2)		
2017	Paiva	FMS	ACR	F	Age-Sex-BMI	DEXA			Fat Mass (kg)
2014	Segura	FMS	ACR	F	Age	BIA		Muscle Mass (kg)	Fat Mass (%)
2016	Sener	FMS	ACR	F	Age-Sex-BMI	BIA			Fat Mass (%)
2013	Srikuea	FMS	ACR	F	Age-Sex-BMI	DEXA		Lean Muscle Mass (kg)	Fat Mass (%)
2016	Vincent	FMS	ACR	F	Sex-BMI	DEXA			Fat Mass (%)

Abbreviations: FMS = Fibromyalgia Syndrome, ACR = American College of Rheumatology, F =Female, BMI = Body Mass Index, BIA = Bio-Electrical Impedance Analysis, DEXA = Dual-energy X-ray absorptiometry.
