# Supplementary material for: Fibromyalgia and Chronic Fatigue Syndromes: A systematic review and meta-analysis of cardiorespiratory fitness and neuromuscular function compared with healthy individuals
Source: PLoS One. 2022 Oct 20;17(10):e0276009. doi: 10.1371/journal.pone.0276009 (PMC9584387; doi:10.1371/journal.pone.0276009)
Supplement: S3 File — RevMan full graphs and sensitivity analysis for all the outcomes included in the systematic review and meta-analysis. (PDF) [file pone.0276009.s003.pdf]

	Patients			Control				Std. Mean Difference	
**Study or Subgroup**	**Mean**	**SD**	**Total**	**Mean**	**SD**	**Total**	**Weight**	**IV, Random, 95% CI**	**Year**
Cook 2022	23.4	8.6	178	29.9	10.9	169	2.4%	−0.66 [−0.88, −0.45]	2022
Van Oosterwijck 2021	16.98	4.25	20	19.96	6.8	20	1.9%	−0.52 [−1.15, 0.12]	2021
Hodges 2020	25.9	3.6	16	38.8	3.6	16	1.2%	−3.49 [−4.64, −2.35]	2020
Nelson 2019	27.3	9.2	10	29.9	6.1	10	1.5%	−0.32 [−1.20, 0.56]	2019
Bouquet 2019	23.2	8.8	14	25.6	4.5	14	1.7%	−0.33 [−1.08, 0.41]	2019
Oosterwijick 2017	16.9	4.2	20	19.9	6.9	20	1.9%	−0.51 [−1.15, 0.12]	2017
Pieroni − Andrade 2017	14.8	3.6	24	19	4.2	20	1.9%	−1.06 [−1.70, −0.42]	2017
Sener 2016	40	10	39	37	8.4	40	2.1%	0.32 [−0.12, 0.77]	2016
Vincent 2016	23.5	5.2	30	28.3	6.9	30	2.0%	−0.78 [−1.30, −0.25]	2016
Gomez − Cabello 2015	21.4	4.9	28	28.8	5.4	22	1.9%	−1.42 [−2.05, −0.79]	2015
Aerenhouts 2015	19.5	0.74	20	29.9	5.5	20	1.5%	−2.60 [−3.46, −1.74]	2015
Bardal 2015	1.45	0.2	16	1.6	0.29	19	1.8%	−0.58 [−1.26, 0.10]	2015
Vermeulen 2014	20.7	5.4	203	27.5	6.9	18	2.1%	−1.23 [−1.72, −0.73]	2014
Balbaloglu 2014	8.3	2.3	30	9.2	2.2	60	2.1%	−0.40 [−0.84, 0.04]	2014
Icksman 2013	19.1	4.6	31	27.2	5.6	13	1.7%	−1.62 [−2.36, −0.88]	2013
Bachassons 2013	23.7	2.7	11	36.1	6.3	11	1.2%	−2.46 [−3.62, −1.30]	2013
Bardal 2013	1.7	0.3	12	2.2	0.5	12	1.5%	−1.17 [−2.05, −0.29]	2013
Cook 2012 M	26.2	9	8	30.3	8.1	13	1.5%	−0.47 [−1.36, 0.43]	2012
Cook 2012 S	781	302	12	907	330	13	1.6%	−0.38 [−1.18, 0.41]	2012
Cook 2012 S	873	290	8	907	330	13	1.5%	−0.10 [−0.98, 0.78]	2012
Gerdle 2013	31.1	6.2	19	29.8	9.7	14	1.8%	0.16 [−0.53, 0.85]	2012
Cook 2012 M	24.2	5.4	12	30.3	8.1	13	1.6%	−0.85 [−1.68, −0.02]	2012
Da Cunha 2011	22	3.7	14	32	7.4	14	1.5%	−1.66 [−2.54, −0.78]	2011
Hsieh 2010	18.6	3.7	31	21	3.2	31	2.0%	−0.69 [−1.20, −0.17]	2010
Nijis 2010	24.1	13.9	22	27.6	24.7	22	1.9%	−0.17 [−0.76, 0.42]	2010
Suarez 2010	17.1	5.5	44	25.6	6	25	2.0%	−1.48 [−2.03, −0.93]	2010
Vermeulen 2010	22.3	5.7	15	31.2	7	15	1.6%	−1.36 [−2.16, −0.55]	2010
Dinler 2009	24.3	2.4	15	27.3	3.4	15	1.7%	−0.99 [−1.76, −0.23]	2009
Patrick − Neary 2008	23.8	10	6	33	3	8	1.2%	−1.26 [−2.45, −0.06]	2008
Valkeinenen 2008	20.6	2.37	23	22	3.1	11	1.7%	−0.52 [−1.25, 0.21]	2008
Dinler 2007	22.6	3.18	18	27.46	4.68	15	1.7%	−1.21 [−1.96, −0.45]	2007
Javierre 2007	14.3	4.6	85	23.4	6.1	15	1.9%	−1.86 [−2.47, −1.26]	2007
Van Ness 2007	26.2	4.9	6	28.2	7.2	6	1.2%	−0.30 [−1.44, 0.84]	2007
Cook 2006	23	4	23	30	8	32	2.0%	−1.04 [−1.61, −0.47]	2006
Cook 2006	26	6	29	29.7	8	32	2.0%	−0.51 [−1.02, −0.00]	2006
Gallagher 2005	27.8	7.6	42	31.3	5.8	42	2.2%	−0.51 [−0.95, −0.08]	2005
Jammes 2005	24	9.2	26	37	11.6	11	1.7%	−1.28 [−2.05, −0.51]	2005
Wallman 2004	16.3	5.6	31	19.9	6.7	31	2.1%	−0.58 [−1.08, −0.07]	2004
Georgiades 2003	21.2	6.3	12	28.3	6.4	11	1.5%	−1.08 [−1.96, −0.19]	2003
Lund 2003	26.6	6	9	36.4	6	9	1.3%	−1.56 [−2.64, −0.47]	2003
Cook 2003	29.8	5.8	19	30.7	4.6	20	1.9%	−0.17 [−0.80, 0.46]	2003
Farquhar 2002	22	4.9	17	33.6	7.8	17	1.6%	−1.74 [−2.54, −0.94]	2002
Valim 2002	26	5.2	50	31	5.6	50	2.2%	−0.92 [−1.33, −0.51]	2002
Inbar 2001	19.8	5.3	12	27.3	5.6	15	1.6%	−1.33 [−2.18, −0.48]	2001
Bazelman 2001	2	0.7	20	2.2	0.6	20	1.9%	−0.30 [−0.92, 0.32]	2001
De Becker 2000 M	22.7	5.01	157	32.9	7.6	163	2.4%	−1.58 [−1.83, −1.32]	2000
De Becker 2000 S	20.5	6.1	427	32	8.5	204	2.4%	−1.65 [−1.84, −1.46]	2000
Fulcher 2000	30.6	8.2	66	34.1	6.8	30	2.1%	−0.45 [−0.88, −0.01]	2000
Rowbottom 1998	34.7	17.2	33	36.2	10.2	20	2.0%	−0.10 [−0.65, 0.46]	1998
Sisto 1996 M	28	5	10	32	4	17	1.6%	−0.88 [−1.71, −0.06]	1996
Sisto 1996 S	23.9	5.6	11	23.4	2.7	5	1.3%	0.10 [−0.96, 1.15]	1996
Mengshoel 1995	28.2	1.8	37	30.2	2.4	20	2.0%	−0.97 [−1.55, −0.40]	1995
Simms 1994	30	8.1	13	32	7.2	13	1.7%	−0.25 [−1.03, 0.52]	1994
Mengshoel 1990	34	23.5	26	36	10.9	26	2.0%	−0.11 [−0.65, 0.44]	1990
Riley 1990	31.8	5.3	13	37.9	5.1	13	1.6%	−1.14 [−1.97, −0.30]	1990
Sargent 2002	35.9	7.7	33	38.6	8.4	33	2.1%	−0.33 [−0.82, 0.15]	
**Total (95% CI)**			**2156**			**1591**	**100.0%**	**−0.86 [−1.04, −0.69]**	

Heterogeneity: $Tau^2 = 0.32$; $Chi^2 = 281.04$, df = 55 ($P < 0.00001$); $I^2 = 80\%$
Test for overall effect: Z = 9.66 ($P < 0.00001$)

Std. Mean Difference
IV, Random, 95% CI

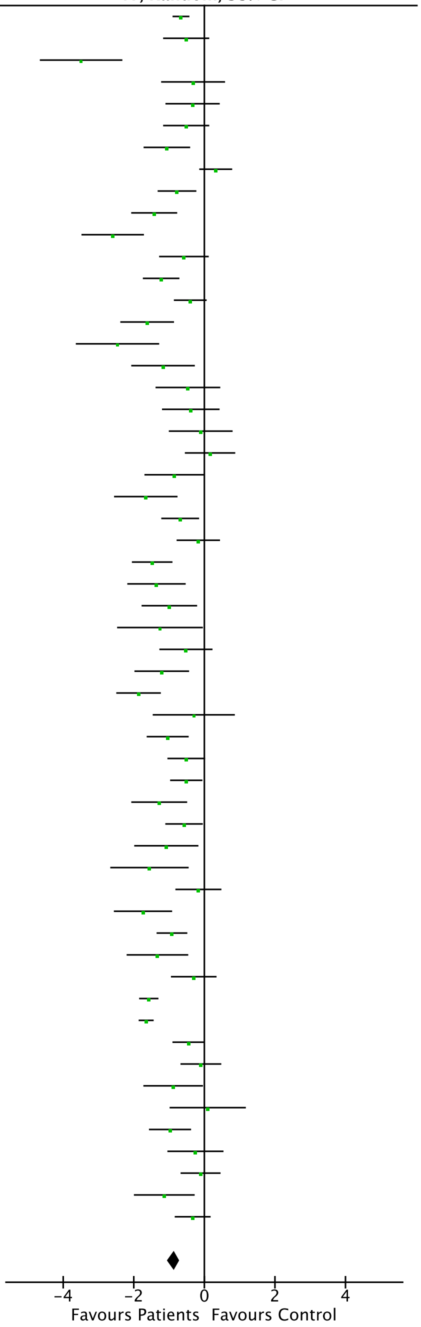

Figure A. Peak Oxygen Consumption (VO_2 Peak) values for all studies included in the analysis.

Study or Subgroup	Patients Mean	Patients SD	Patients Total	Control Mean	Control SD	Control Total	Weight	Std. Mean Difference IV, Random, 95% CI	Year
Cook 2022	23.4	8.6	178	29.9	10.9	169	6.0%	−0.66 [−0.88, −0.45]	2022
Bouquet 2019	23.2	8.8	14	25.6	4.5	14	2.6%	−0.33 [−1.08, 0.41]	2019
Nelson 2019	27.3	9.2	10	29.9	6.1	10	2.1%	−0.32 [−1.20, 0.56]	2019
Oosterwijick 2017	16.9	4.2	20	19.9	6.9	20	3.2%	−0.51 [−1.15, 0.12]	2017
Sener 2016	40	10	39	37	8.4	40	4.3%	0.32 [−0.12, 0.77]	2016
Bardal 2015	1.45	0.2	16	1.6	0.29	19	2.9%	−0.58 [−1.26, 0.10]	2015
Vermeulen 2014	20.7	5.4	203	27.5	6.9	18	4.0%	−1.23 [−1.72, −0.73]	2014
Cook 2012 S	873	290	8	907	330	13	2.1%	−0.10 [−0.98, 0.78]	2012
Cook 2012 M	24.2	5.4	12	30.3	8.1	13	2.3%	−0.85 [−1.68, −0.02]	2012
Cook 2012 M	26.2	9	8	30.3	8.1	13	2.1%	−0.47 [−1.36, 0.43]	2012
Cook 2012 S	781	302	12	907	330	13	2.4%	−0.38 [−1.18, 0.41]	2012
Da Cunha 2011	22	3.7	14	32	7.4	14	2.1%	−1.66 [−2.54, −0.78]	2011
Nijis 2010	24.1	13.9	22	27.6	24.7	22	3.4%	−0.17 [−0.76, 0.42]	2010
Vermeulen 2010	22.3	5.7	15	31.2	7	15	2.4%	−1.36 [−2.16, −0.55]	2010
Patrick – Neary 2008	23.8	10	6	33	3	8	1.3%	−1.26 [−2.45, −0.06]	2008
Cook 2006	23	4	23	30	8	32	3.5%	−1.04 [−1.61, −0.47]	2006
Cook 2006	26	6	29	29.7	8	32	3.9%	−0.51 [−1.02, −0.00]	2006
Jammes 2005	24	9.2	26	37	11.6	11	2.5%	−1.28 [−2.05, −0.51]	2005
Gallagher 2005	27.8	7.6	42	31.3	5.8	42	4.4%	−0.51 [−0.95, −0.08]	2005
Wallman 2004	16.3	5.6	31	19.9	6.7	31	3.9%	−0.58 [−1.08, −0.07]	2004
Cook 2003	29.8	5.8	19	30.7	4.6	20	3.2%	−0.17 [−0.80, 0.46]	2003
Lund 2003	26.6	6	9	36.4	6	9	1.5%	−1.56 [−2.64, −0.47]	2003
Georgiades 2003	21.2	6.3	12	28.3	6.4	11	2.1%	−1.08 [−1.96, −0.19]	2003
Valim 2002	26	5.2	50	31	5.6	50	4.6%	−0.92 [−1.33, −0.51]	2002
Bazelman 2001	2	0.7	20	2.2	0.6	20	3.2%	−0.30 [−0.92, 0.32]	2001
Inbar 2001	19.8	5.3	12	27.3	5.6	15	2.2%	−1.33 [−2.18, −0.48]	2001
Fulcher 2000	30.6	8.2	66	34.1	6.8	30	4.4%	−0.45 [−0.88, −0.01]	2000
Rowbottom 1998	34.7	17.2	33	36.2	10.2	20	3.6%	−0.10 [−0.65, 0.46]	1998
Sisto 1996 S	23.9	5.6	11	23.4	2.7	5	1.6%	0.10 [−0.96, 1.15]	1996
Sisto 1996 M	28	5	10	32	4	17	2.3%	−0.88 [−1.71, −0.06]	1996
Mengshoel 1995	28.2	1.8	37	30.2	2.4	20	3.5%	−0.97 [−1.55, −0.40]	1995
Simms 1994	30	8.1	13	32	7.2	13	2.5%	−0.25 [−1.03, 0.52]	1994
Sargent 2002	35.9	7.7	33	38.6	8.4	33	4.0%	−0.33 [−0.82, 0.15]	
**Total (95% CI)**			**1053**			**812**	**100.0%**	**−0.61 [−0.77, −0.46]**	

Heterogeneity: $Tau^2 = 0.09$; $Chi^2 = 64.78$, df = 32 (P = 0.0005); $I^2 = 51\%$

Test for overall effect: Z = 7.86 (P < 0.00001)

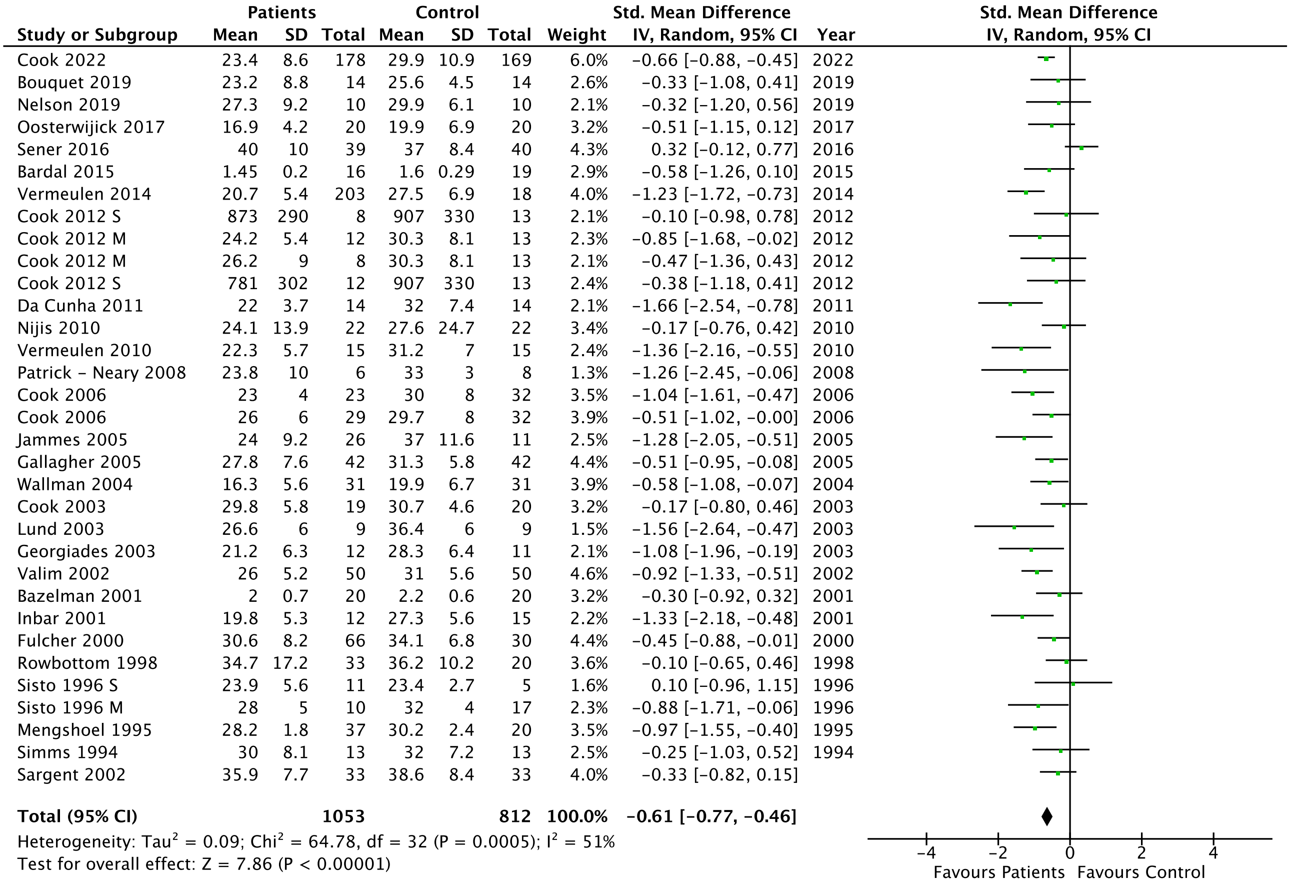

Figure A1. Peak Oxygen Consumption (VO_2 Peak) values after sensitivity analysis.

Study or Subgroup	Patients Mean	Patients SD	Patients Total	Control Mean	Control SD	Control Total	Weight	Std. Mean Difference IV, Random, 95% CI	Year
**1.3.1 CFS**									
Sargent 2002	35.9	7.7	33	38.6	8.4	33	2.1%	−0.33 [−0.82, 0.15]	
Riley 1990	31.8	5.3	13	37.9	5.1	13	1.6%	−1.14 [−1.97, −0.30]	1990
Sisto 1996 M	28	5	10	32	4	17	1.6%	−0.88 [−1.71, −0.06]	1996
Sisto 1996 S	23.9	5.6	11	23.4	2.7	5	1.3%	0.10 [−0.96, 1.15]	1996
Rowbottom 1998	34.7	17.2	33	36.2	10.2	20	2.0%	−0.10 [−0.65, 0.46]	1998
De Becker 2000 M	22.7	5.01	157	32.9	7.6	163	2.4%	−1.58 [−1.83, −1.32]	2000
De Becker 2000 S	20.5	6.1	427	32	8.5	204	2.4%	−1.65 [−1.84, −1.46]	2000
Fulcher 2000	30.6	8.2	66	34.1	6.8	30	2.1%	−0.45 [−0.88, −0.01]	2000
Inbar 2001	19.8	5.3	12	27.3	5.6	15	1.6%	−1.33 [−2.18, −0.48]	2001
Bazelman 2001	2	0.7	20	2.2	0.6	20	1.9%	−0.30 [−0.92, 0.32]	2001
Farquhar 2002	22	4.9	17	33.6	7.8	17	1.6%	−1.74 [−2.54, −0.94]	2002
Georgiades 2003	21.2	6.3	12	28.3	6.4	11	1.5%	−1.08 [−1.96, −0.19]	2003
Cook 2003	29.8	5.8	19	30.7	4.6	20	1.9%	−0.17 [−0.80, 0.46]	2003
Wallman 2004	16.3	5.6	31	19.9	6.7	31	2.1%	−0.58 [−1.08, −0.07]	2004
Gallagher 2005	27.8	7.6	42	31.3	5.8	42	2.2%	−0.51 [−0.95, −0.08]	2005
Jammes 2005	24	9.2	26	37	11.6	11	1.7%	−1.28 [−2.05, −0.51]	2005
Cook 2006	23	4	23	30	8	32	2.0%	−1.04 [−1.61, −0.47]	2006
Javierre 2007	14.3	4.6	85	23.4	6.1	15	1.9%	−1.86 [−2.47, −1.26]	2007
Van Ness 2007	26.2	4.9	6	28.2	7.2	6	1.2%	−0.30 [−1.44, 0.84]	2007
Patrick – Neary 2008	23.8	10	6	33	3	8	1.2%	−1.26 [−2.45, −0.06]	2008
Nijis 2010	24.1	13.9	22	27.6	24.7	22	1.9%	−0.17 [−0.76, 0.42]	2010
Suarez 2010	17.1	5.5	44	25.6	6	25	2.0%	−1.48 [−2.03, −0.93]	2010
Vermeulen 2010	22.3	5.7	15	31.2	7	15	1.6%	−1.36 [−2.16, −0.55]	2010
Cook 2012 M	26.2	9	8	30.3	8.1	13	1.5%	−0.47 [−1.36, 0.43]	2012
Cook 2012 S	781	302	12	907	330	13	1.6%	−0.38 [−1.18, 0.41]	2012
Icksman 2013	19.1	4.6	31	27.2	5.6	13	1.7%	−1.62 [−2.36, −0.88]	2013
Vermeulen 2014	20.7	5.4	203	27.5	6.9	18	2.1%	−1.23 [−1.72, −0.73]	2014
Balbaloglu 2014	8.3	2.3	30	9.2	2.2	60	2.1%	−0.40 [−0.84, 0.04]	2014
Aerenhouts 2015	19.5	0.74	20	29.9	5.5	20	1.5%	−2.60 [−3.46, −1.74]	2015
Oosterwijick 2017	16.9	4.2	20	19.9	6.9	20	1.9%	−0.51 [−1.15, 0.12]	2017
Bouquet 2019	23.2	8.8	14	25.6	4.5	14	1.7%	−0.33 [−1.08, 0.41]	2019
Nelson 2019	27.3	9.2	10	29.9	6.1	10	1.5%	−0.32 [−1.20, 0.56]	2019
Hodges 2020	25.9	3.6	16	38.8	3.6	16	1.2%	−3.49 [−4.64, −2.35]	2020
Van Oosterwijck 2021	16.98	4.25	20	19.96	6.8	20	1.9%	−0.52 [−1.15, 0.12]	2021
Cook 2022	23.4	8.6	178	29.9	10.9	169	2.4%	−0.66 [−0.88, −0.45]	2022
**Subtotal (95% CI)**			**1692**			**1161**	**62.8%**	**−0.92 [−1.14, −0.69]**	

Heterogeneity: $Tau^2 = 0.34$; $Chi^2 = 201.28$, df = 34 ($P < 0.00001$); $I^2 = 83\%$
Test for overall effect: Z = 7.99 ($P < 0.00001$)

Study or Subgroup	Patients Mean	Patients SD	Patients Total	Control Mean	Control SD	Control Total	Weight	Std. Mean Difference IV, Random, 95% CI	Year
**1.3.2 FMS**									
Mengshoel 1990	34	23.5	26	36	10.9	26	2.0%	−0.11 [−0.65, 0.44]	1990
Simms 1994	30	8.1	13	32	7.2	13	1.7%	−0.25 [−1.03, 0.52]	1994
Mengshoel 1995	28.2	1.8	37	30.2	2.4	20	2.0%	−0.97 [−1.55, −0.40]	1995
Valim 2002	26	5.2	50	31	5.6	50	2.2%	−0.92 [−1.33, −0.51]	2002
Lund 2003	26.6	6	9	36.4	6	9	1.3%	−1.56 [−2.64, −0.47]	2003
Cook 2006	26	6	29	29.7	8	32	2.0%	−0.51 [−1.02, −0.00]	2006
Dinler 2007	22.6	3.18	18	27.46	4.68	15	1.7%	−1.21 [−1.96, −0.45]	2007
Valkeinenen 2008	20.6	2.37	23	22	3.1	11	1.7%	−0.52 [−1.25, 0.21]	2008
Dinler 2009	24.3	2.4	15	27.3	3.4	15	1.7%	−0.99 [−1.76, −0.23]	2009
Hsieh 2010	18.6	3.7	31	21	3.2	31	2.0%	−0.69 [−1.20, −0.17]	2010
Da Cunha 2011	22	3.7	14	32	7.4	14	1.5%	−1.66 [−2.54, −0.78]	2011
Cook 2012 M	24.2	5.4	12	30.3	8.1	13	1.6%	−0.85 [−1.68, −0.02]	2012
Cook 2012 S	873	290	8	907	330	13	1.5%	−0.10 [−0.98, 0.78]	2012
Gerdle 2013	31.1	6.2	19	29.8	9.7	14	1.8%	0.16 [−0.53, 0.85]	2012
Bardal 2013	1.7	0.3	12	2.2	0.5	12	1.5%	−1.17 [−2.05, −0.29]	2013
Bachassons 2013	23.7	2.7	11	36.1	6.3	11	1.2%	−2.46 [−3.62, −1.30]	2013
Bardal 2015	1.45	0.2	16	1.6	0.29	19	1.8%	−0.58 [−1.26, 0.10]	2015
Gomez – Cabello 2015	21.4	4.9	28	28.8	5.4	22	1.9%	−1.42 [−2.05, −0.79]	2015
Vincent 2016	23.5	5.2	30	28.3	6.9	30	2.0%	−0.78 [−1.30, −0.25]	2016
Sener 2016	40	10	39	37	8.4	40	2.1%	0.32 [−0.12, 0.77]	2016
Pieroni – Andrade 2017	14.8	3.6	24	19	4.2	20	1.9%	−1.06 [−1.70, −0.42]	2017
**Subtotal (95% CI)**			**464**			**430**	**37.2%**	**−0.76 [−1.01, −0.51]**	

Heterogeneity: $Tau^2 = 0.23$; $Chi^2 = 62.35$, df = 20 ($P < 0.00001$); $I^2 = 68\%$
Test for overall effect: Z = 5.86 ($P < 0.00001$)

Study or Subgroup	Patients Mean	Patients SD	Patients Total	Control Mean	Control SD	Control Total	Weight	Std. Mean Difference IV, Random, 95% CI	Year
**Total (95% CI)**			**2156**			**1591**	**100.0%**	**−0.86 [−1.04, −0.69]**	

Heterogeneity: $Tau^2 = 0.32$; $Chi^2 = 281.04$, df = 55 ($P < 0.00001$); $I^2 = 80\%$
Test for overall effect: Z = 9.66 ($P < 0.00001$)
Test for subgroup differences: $Chi^2 = 0.84$, df = 1 ($P = 0.36$), $I^2 = 0\%$

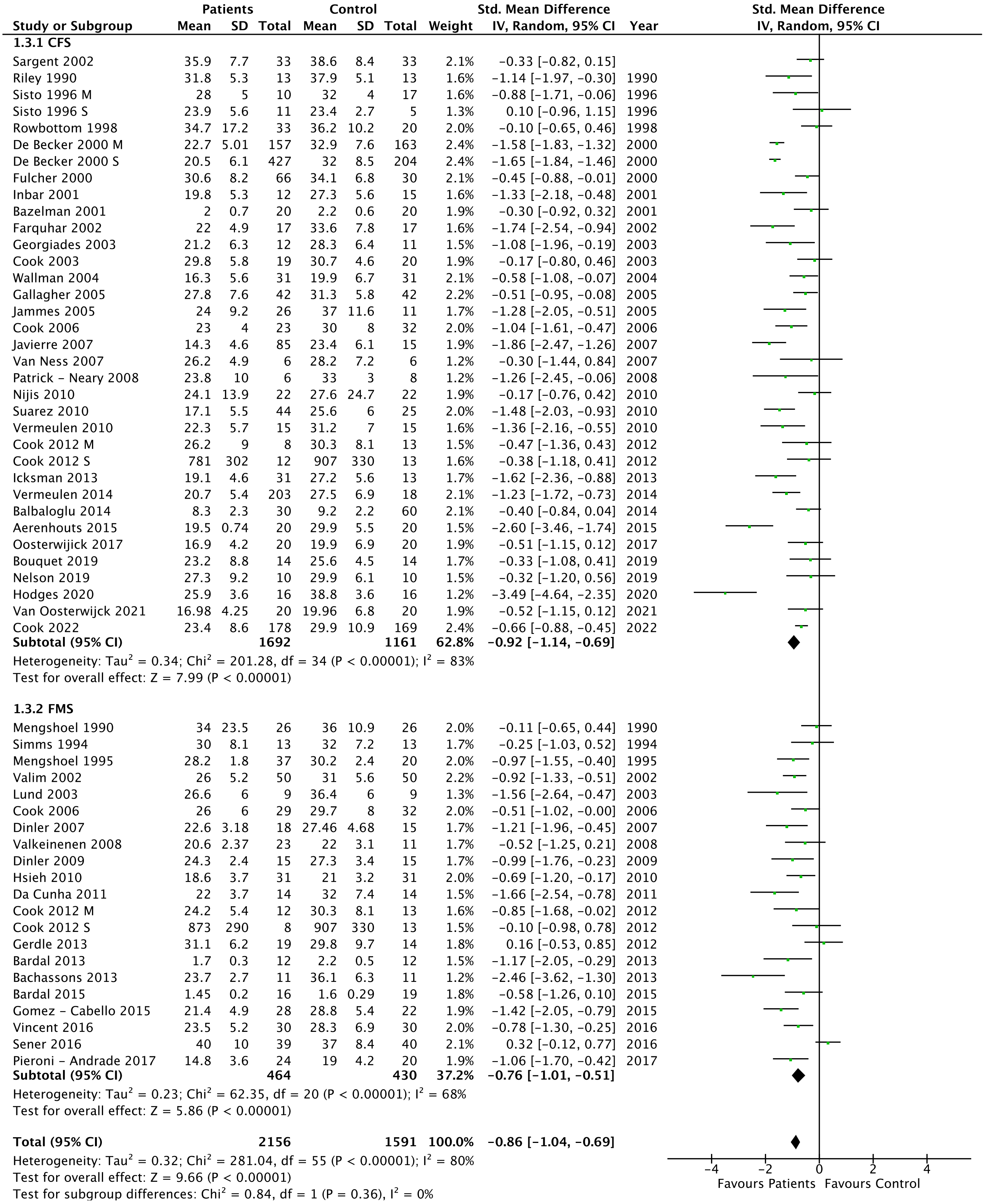

Figure A2. Peak Oxygen Consumption (VO_2 Peak) values between CFS and FMS

Study or Subgroup	Patients Mean	Patients SD	Patients Total	Controls Mean	Controls SD	Controls Total	Weight	Std. Mean Difference IV, Random, 95% CI	Year
Cook 2022	19.2	1	178	18.2	2	169	4.6%	0.64 [0.42, 0.85]	2022
Van Oosterwijck 2021	16	0.3	20	12	2	20	2.9%	2.74 [1.86, 3.63]	2021
Berardi 2021	6.4	2.4	47	4.6	2.6	47	4.2%	0.71 [0.30, 1.13]	2021
Berardi 2021	7.1	2.2	47	5.9	2.4	47	4.2%	0.52 [0.11, 0.93]	2021
Hodges 2020	14.6	1.7	16	13.9	2.9	16	3.4%	0.29 [−0.41, 0.98]	2020
Nelson 2019	13	1.4	10	10.8	1.9	10	2.6%	1.26 [0.28, 2.24]	2019
Bardal 2015	14.7	2.5	16	14.2	1.5	19	3.5%	0.24 [−0.43, 0.91]	2015
Bachassons 2013	5.5	1.4	11	4.6	2.9	11	3.0%	0.38 [−0.46, 1.23]	2013
Bardal 2013	18.5	1.5	12	18.6	0.9	12	3.1%	−0.08 [−0.88, 0.72]	2013
Bardal 2013	14.2	2.2	12	14.2	1.6	12	3.1%	0.00 [−0.80, 0.80]	2013
Strahler 2013	17.4	2.1	21	17.6	0.9	20	3.6%	−0.12 [−0.73, 0.49]	2013
Cook 2012 S	10.6	1.6	8	9	1.9	13	2.8%	0.86 [−0.07, 1.78]	2012
Cook 2012 S	12.2	2.7	12	9	1.9	13	2.9%	1.34 [0.45, 2.22]	2012
Hsieh 2010	7.9	0.9	31	8.2	0.7	31	3.9%	−0.37 [−0.87, 0.13]	2010
Suarez 2010	18.3	1.8	44	17.2	2.7	25	3.9%	0.50 [0.00, 1.00]	2010
Dinler 2007	8.3	1.6	18	8.4	1.1	15	3.4%	−0.07 [−0.76, 0.62]	2007
Javierre 2007	19.4	0.9	85	19.1	1.2	15	3.8%	0.31 [−0.24, 0.86]	2007
Cook 2006	11.2	2	29	10	2	32	3.9%	0.59 [0.08, 1.11]	2006
Cook 2006	13	2	23	10	2	32	3.6%	1.48 [0.87, 2.09]	2006
Staud 2005	15.4	2.6	12	12	4.7	11	2.9%	0.87 [0.01, 1.74]	2005
Wallman 2004	0.16	0.07	31	0.1	0.02	31	3.8%	1.15 [0.61, 1.69]	2004
Cook 2003	12.3	1.9	19	10	2.4	20	3.5%	1.04 [0.36, 1.71]	2003
Bazelman 2001	3.82	0.88	20	2.44	0.86	20	3.3%	1.55 [0.84, 2.27]	2001
Blackwood 1998	7.6	4.3	10	4.3	2.58	10	2.8%	0.89 [−0.04, 1.82]	2001
Sacco 1999	13.5	1.2	10	11.2	1.2	10	2.4%	1.84 [0.75, 2.92]	1999
Sisto 1996 M	13	2.2	10	11	1.6	17	3.0%	1.05 [0.22, 1.89]	1996
Sisto 1996 S	15	1.9	11	12	1.1	5	2.1%	1.66 [0.41, 2.91]	1996
Mengshoel 1995	17	2	37	13	2.5	20	3.5%	1.81 [1.16, 2.45]	1995
Mengshoel 1990	16.3	1.2	26	13.8	0.7	26	3.3%	2.51 [1.77, 3.25]	1990
Riley 1990	8.2	1.9	13	6.6	0.8	13	3.0%	1.06 [0.23, 1.89]	1990
**Total (95% CI)**			**839**			**742**	**100.0%**	**0.84 [0.60, 1.08]**	

Heterogeneity: Tau^2 = 0.31; Chi^2 = 124.69, df = 29 ($P < 0.00001$); I^2 = 77%
Test for overall effect: Z = 6.90 ($P < 0.00001$)

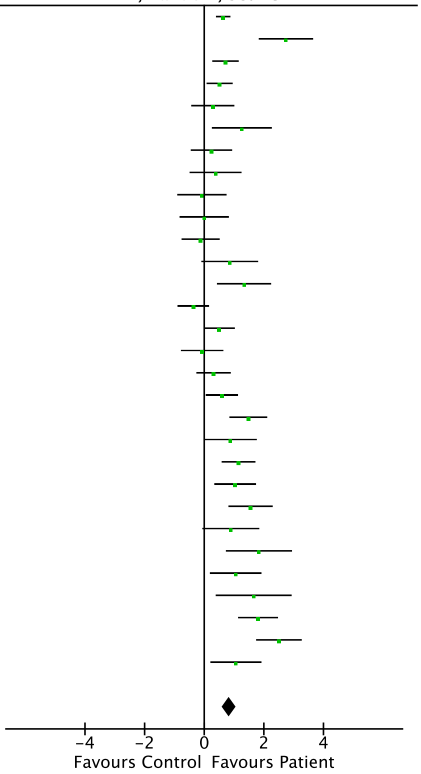

Figure B. Rate of Perceived Exertion (RPE) values for all studies included in the analysis.

Study or Subgroup	Patients Mean	Patients SD	Patients Total	Controls Mean	Controls SD	Controls Total	Weight	Std. Mean Difference IV, Random, 95% CI	Year
Berardi 2021	6.4	2.4	47	4.6	2.6	47	10.0%	0.71 [0.30, 1.13]	2021
Berardi 2021	7.1	2.2	47	5.9	2.4	47	10.1%	0.52 [0.11, 0.93]	2021
Nelson 2019	13	1.4	10	10.8	1.9	10	4.5%	1.26 [0.28, 2.24]	2019
Bardal 2015	14.7	2.5	16	14.2	1.5	19	7.0%	0.24 [−0.43, 0.91]	2015
Cook 2012 S	10.6	1.6	8	9	1.9	13	4.9%	0.86 [−0.07, 1.78]	2012
Cook 2012 S	12.2	2.7	12	9	1.9	13	5.2%	1.34 [0.45, 2.22]	2012
Cook 2006	11.2	2	29	10	2	32	8.8%	0.59 [0.08, 1.11]	2006
Cook 2006	13	2	23	10	2	32	7.7%	1.48 [0.87, 2.09]	2006
Wallman 2004	0.16	0.07	31	0.1	0.02	31	8.5%	1.15 [0.61, 1.69]	2004
Cook 2003	12.3	1.9	19	10	2.4	20	7.0%	1.04 [0.36, 1.71]	2003
Bazelman 2001	3.82	0.88	20	2.44	0.86	20	6.6%	1.55 [0.84, 2.27]	2001
Sacco 1999	13.5	1.2	10	11.2	1.2	10	3.9%	1.84 [0.75, 2.92]	1999
Sisto 1996 S	15	1.9	11	12	1.1	5	3.2%	1.66 [0.41, 2.91]	1996
Sisto 1996 M	13	2.2	10	11	1.6	17	5.5%	1.05 [0.22, 1.89]	1996
Mengshoel 1995	17	2	37	13	2.5	20	7.3%	1.81 [1.16, 2.45]	1995
**Total (95% CI)**			**330**			**336**	**100.0%**	**1.06 [0.81, 1.31]**	

Heterogeneity: Tau^2 = 0.12; Chi^2 = 29.67, df = 14 ($P = 0.008$); I^2 = 53%
Test for overall effect: Z = 8.18 ($P < 0.00001$)

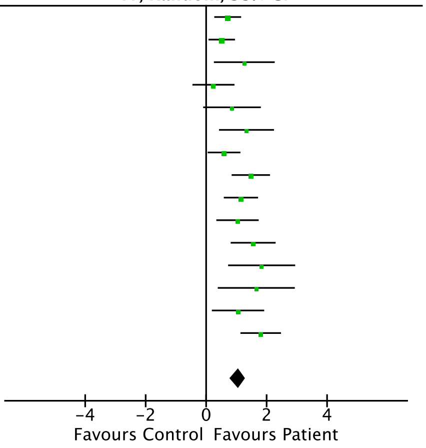

Figure B1. Rate of Perceived Exertion (RPE) values for all studies after sensitivity analysis.

Study or Subgroup	Patients Mean	Patients SD	Patients Total	Controls Mean	Controls SD	Controls Total	Weight	Std. Mean Difference IV, Random, 95% CI	Year
**2.3.1 FMS**									
Mengshoel 1990	16.3	1.2	26	13.8	0.7	26	2.8%	2.51 [1.77, 3.25]	1990
Mengshoel 1995	17	2	37	13	2.5	20	3.0%	1.81 [1.16, 2.45]	1995
Mengshoel 1995	17	2	37	13	2.5	20	3.0%	1.81 [1.16, 2.45]	1995
Staud 2005	15.4	2.6	12	12	4.7	11	2.5%	0.87 [0.01, 1.74]	2005
Cook 2006	13	2	23	10	2	32	3.1%	1.48 [0.87, 2.09]	2006
Dinler 2007	8.3	1.6	18	8.4	1.1	15	2.9%	−0.07 [−0.76, 0.62]	2007
Hsieh 2010	7.9	0.9	31	8.2	0.7	31	3.3%	−0.37 [−0.87, 0.13]	2010
Cook 2012 S	12.2	2.7	12	9	1.9	13	2.5%	1.34 [0.45, 2.22]	2012
Cook 2012 S	10.6	1.6	8	9	1.9	13	2.4%	0.86 [−0.07, 1.78]	2012
Bachassons 2013	5.5	1.4	11	4.6	2.9	11	2.5%	0.38 [−0.46, 1.23]	2013
Bardal 2013	18.5	1.5	12	18.6	0.9	12	2.6%	−0.08 [−0.88, 0.72]	2013
Bardal 2013	14.2	2.2	12	14.2	1.6	12	2.6%	0.00 [−0.80, 0.80]	2013
Bardal 2015	14.7	2.5	16	14.2	1.5	19	3.0%	0.24 [−0.43, 0.91]	2015
Berardi 2021	7.1	2.2	47	5.9	2.4	47	3.6%	0.52 [0.11, 0.93]	2021
Berardi 2021	6.4	2.4	47	4.6	2.6	47	3.5%	0.71 [0.30, 1.13]	2021
**Subtotal (95% CI)**			**349**			**329**	**43.4%**	**0.80 [0.38, 1.21]**	
Heterogeneity: Tau² = 0.55; Chi² = 85.87, df = 14 (P < 0.00001); I² = 84%									
Test for overall effect: Z = 3.75 (P = 0.0002)									
**2.3.2 CFS**									
Riley 1990	8.2	1.9	13	6.6	0.8	13	2.6%	1.06 [0.23, 1.89]	1990
Sisto 1996 M	13	2.2	10	11	1.6	17	2.6%	1.05 [0.22, 1.89]	1996
Sisto 1996 S	15	1.9	11	12	1.1	5	1.8%	1.66 [0.41, 2.91]	1996
Sisto 1996 M	13	2.2136	10	11	1.6492	17	2.6%	1.04 [0.20, 1.87]	1996
Sisto 1996 S	15	1.99	11	12	1.118	5	1.8%	1.59 [0.36, 2.82]	1996
Sacco 1999	13.5	1.2	10	11.2	1.2	10	2.1%	1.84 [0.75, 2.92]	1999
Sacco 1999	13.5	1.2649	10	11.2	1.2649	10	2.1%	1.74 [0.68, 2.80]	1999
Bazelman 2001	3.82	0.88	20	2.44	0.86	20	2.8%	1.55 [0.84, 2.27]	2001
Blackwood 1998	7.6	4.3	10	4.3	2.58	10	2.4%	0.89 [−0.04, 1.82]	2001
Cook 2003	12.3	1.9	19	10	2.4	20	2.9%	1.04 [0.36, 1.71]	2003
Wallman 2004	0.16	0.07	31	0.1	0.02	31	3.3%	1.15 [0.61, 1.69]	2004
Cook 2006	11.2	2	29	10	2	32	3.3%	0.59 [0.08, 1.11]	2006
Javierre 2007	19.4	0.9	85	19.1	1.2	15	3.2%	0.31 [−0.24, 0.86]	2007
Suarez 2010	18.3	1.8	44	17.2	2.7	25	3.4%	0.50 [0.00, 1.00]	2010
Cook 2012 S	10.6	1.6	8	9	1.9	13	2.4%	0.86 [−0.07, 1.78]	2012
Strahler 2013	17.4	2.1	21	17.6	0.9	20	3.1%	−0.12 [−0.73, 0.49]	2013
Bardal 2015	14.7	2.5	15	14.2	1.5	19	2.9%	0.24 [−0.44, 0.92]	2015
Nelson 2019	13	1.4	10	10.8	1.9	10	2.3%	1.26 [0.28, 2.24]	2019
Hodges 2020	14.6	1.7	16	13.9	2.9	16	2.9%	0.29 [−0.41, 0.98]	2020
Van Oosterwijck 2021	16	0.3	20	12	2	20	2.5%	2.74 [1.86, 3.63]	2021
Cook 2022	19.2	1	178	18.2	2	169	3.9%	0.64 [0.42, 0.85]	2022
**Subtotal (95% CI)**			**581**			**497**	**56.6%**	**0.94 [0.69, 1.19]**	
Heterogeneity: Tau² = 0.19; Chi² = 57.05, df = 20 (P < 0.0001); I² = 65%									
Test for overall effect: Z = 7.31 (P < 0.00001)									
**Total (95% CI)**			**930**			**826**	**100.0%**	**0.89 [0.67, 1.11]**	
Heterogeneity: Tau² = 0.31; Chi² = 143.04, df = 35 (P < 0.00001); I² = 76%									
Test for overall effect: Z = 7.89 (P < 0.00001)									
Test for subgroup differences: Chi² = 0.33, df = 1 (P = 0.56), I² = 0%									

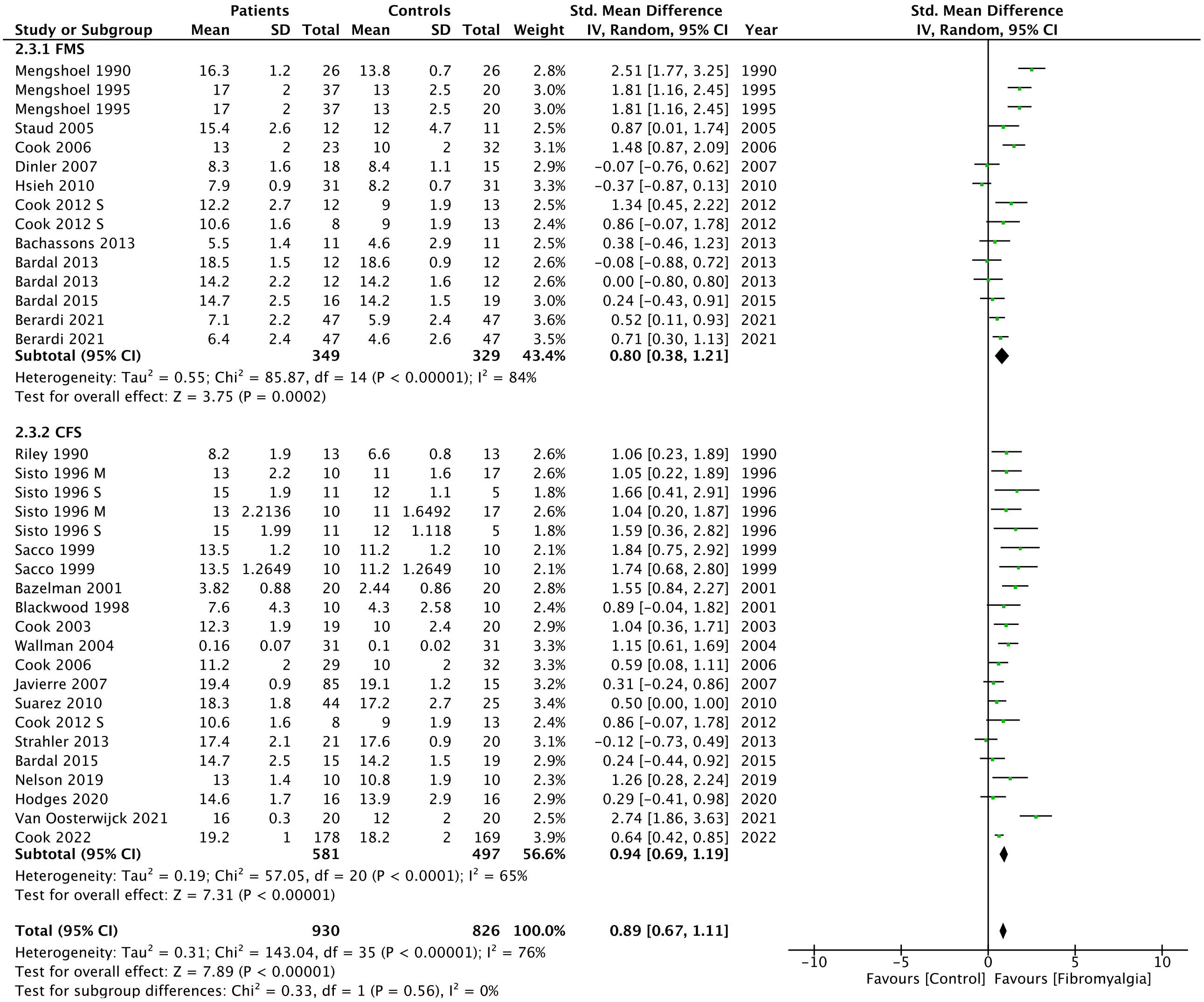

Figure B2. Rate of Perceived Exertion (RPE) values for all studies between CFS and FMS patients.

Study or Subgroup	Patient Mean	Patient SD	Patient Total	Control Mean	Control SD	Control Total	Weight	Std. Mean Difference IV, Random, 95% CI	Year
Cook 2022	156	20.2	178	166.5	17.6	169	4.6%	−0.55 [−0.77, −0.34]	2022
Van Oosterwijck 2021	140	9	20	142	10	20	2.5%	−0.21 [−0.83, 0.42]	2021
Hodges 2020	162	14	16	165	18	16	2.2%	−0.18 [−0.88, 0.51]	2020
Nelson 2019	170	10	10	167	15.7	10	1.6%	0.22 [−0.66, 1.10]	2019
Oosterwijick 2017	140	9	20	142	10	20	2.5%	−0.21 [−0.83, 0.42]	2017
Pieroni – Andrade 2017	131	18	24	148	12	20	2.4%	−1.07 [−1.71, −0.43]	2017
Vincent 2016	165.5	13.8	30	169.6	12.5	30	3.0%	−0.31 [−0.82, 0.20]	2016
Bardal 2015	134	13.7	16	140	17.1	19	2.3%	−0.37 [−1.05, 0.30]	2015
Balbaloglu 2014	166	11	30	165	9	60	3.4%	0.10 [−0.34, 0.54]	2014
Vermeulen 2014	157.9	19.3	203	163.6	15.1	18	3.1%	−0.30 [−0.78, 0.18]	2014
Bachassons 2013	152	20	11	172	10	11	1.5%	−1.22 [−2.14, −0.29]	2013
Bardal 2013	165.8	15.5	12	177.8	12.8	12	1.7%	−0.82 [−1.65, 0.02]	2013
Icksman 2013	145.1	22.4	31	164.7	7	31	2.8%	−1.17 [−1.71, −0.63]	2013
Strahler 2013	159.7	20.5	21	162.8	1.5	20	2.5%	−0.21 [−0.82, 0.41]	2013
Cook 2012 S	101	9.6	8	105	18.6	13	1.6%	−0.24 [−1.13, 0.64]	2012
Cook 2012 S	104	13.3	12	105	18.6	13	1.9%	−0.06 [−0.84, 0.73]	2012
Da Cunha 2011	148	18.7	14	177	11.2	14	1.6%	−1.83 [−2.73, −0.92]	2011
Hsieh 2010	145.8	16.7	31	158.9	13.7	31	2.9%	−0.85 [−1.37, −0.33]	2010
Suarez 2010	132	25.1	44	164.6	17.2	25	2.8%	−1.43 [−1.98, −0.88]	2010
Vermeulen 2010	158	20	15	167	8	15	2.1%	−0.57 [−1.31, 0.16]	2010
Patrick – Neary 2008	154	13	6	186	11	8	0.7%	−2.52 [−4.05, −0.99]	2008
Valkeinenen 2008	158	7.9	23	177.3	3.39	11	1.3%	−2.76 [−3.77, −1.76]	2008
Cook 2006	163	20	23	173	16	32	2.8%	−0.55 [−1.10, −0.01]	2006
Cook 2006	169	14	29	173	16	32	3.0%	−0.26 [−0.77, 0.24]	2006
Gallagher 2005	178	5.05	42	182.6	3.2	42	3.2%	−1.08 [−1.54, −0.62]	2005
Cook 2003	174	14	19	183	11	20	2.4%	−0.70 [−1.35, −0.05]	2003
Georgiades 2003	160	29	12	176	13	11	1.7%	−0.68 [−1.52, 0.17]	2003
Lund 2003	160.5	19.7	9	176.8	15.4	9	1.4%	−0.88 [−1.86, 0.10]	2003
Farquhar 2002	169	20.6	17	176	12.3	17	2.2%	−0.40 [−1.08, 0.28]	2002
Valim 2002	168	13.6	50	174	13.2	50	3.6%	−0.44 [−0.84, −0.05]	2002
Bazelman 2001	165	16.2	20	173	13.4	20	2.4%	−0.53 [−1.16, 0.10]	2001
Inbar 2001	150	19	12	172	10.5	15	1.7%	−1.44 [−2.30, −0.57]	2001
De Becker 2000 M	167	12.5	157	175	10.2	163	4.5%	−0.70 [−0.93, −0.48]	2000
De Becker 2000 S	151	24.7	427	171	18.5	204	4.8%	−0.87 [−1.05, −0.70]	2000
Fulcher 2000	171	18	66	182	14	30	3.3%	−0.65 [−1.09, −0.21]	2000
Rowbottom 1998	160	24	16	175	16	16	2.1%	−0.72 [−1.43, 0.00]	1998
Sisto 1996 M	176	11.6	10	182	10.4	17	1.8%	−0.54 [−1.33, 0.26]	1996
Sisto 1996 S	146	21.4	11	160	14.3	5	1.2%	−0.67 [−1.76, 0.42]	1996
Simms 1994	176	8.5	13	179	13.9	13	1.9%	−0.25 [−1.02, 0.52]	1994
Riley 1990	177	18	13	182	7	13	1.9%	−0.35 [−1.13, 0.42]	1990
Sargent 2002	183.4	10.3	33	188.9	11.2	33	3.1%	−0.51 [−1.00, −0.01]	
**Total (95% CI)**			**1754**			**1328**	**100.0%**	**−0.64 [−0.77, −0.50]**	

Heterogeneity: $Tau^2 = 0.09$; $Chi^2 = 94.73$, df = 40 ($P < 0.00001$); $I^2 = 58\%$
Test for overall effect: $Z = 9.25$ ($P < 0.00001$)

Std. Mean Difference
IV, Random, 95% CI

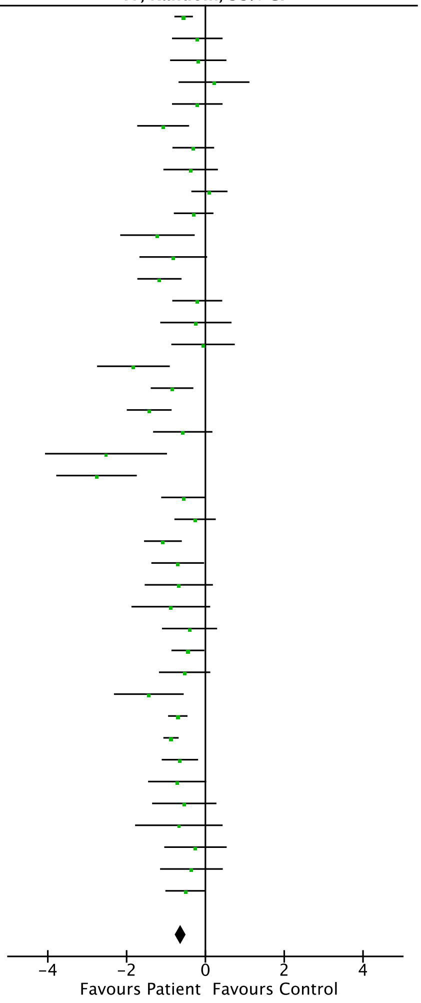

Figure C. Peak Heart Rate (HR) values for all studies included in the analysis.

Study or Subgroup	Patient Mean	Patient SD	Patient Total	Control Mean	Control SD	Control Total	Weight	Std. Mean Difference IV, Random, 95% CI	Year
Cook 2022	156	20.2	178	166.5	17.6	169	11.8%	−0.55 [−0.77, −0.34]	2022
Cook 2022	156	20.2	178	166.5	17.6	169	11.8%	−0.55 [−0.77, −0.34]	2022
Nelson 2019	170	10	10	167	15.7	10	1.9%	0.22 [−0.66, 1.10]	2019
Oosterwijick 2017	140	9	20	142	10	20	3.5%	−0.21 [−0.83, 0.42]	2017
Bardal 2015	134	13.7	16	140	17.1	19	3.1%	−0.37 [−1.05, 0.30]	2015
Vermeulen 2014	157.9	19.3	203	163.6	15.1	18	5.1%	−0.30 [−0.78, 0.18]	2014
Cook 2012 S	101	9.6	8	105	18.6	13	1.9%	−0.24 [−1.13, 0.64]	2012
Cook 2012 S	104	13.3	12	105	18.6	13	2.4%	−0.06 [−0.84, 0.73]	2012
Da Cunha 2011	148	18.7083	14	177	11.225	14	1.9%	−1.83 [−2.73, −0.92]	2011
Vermeulen 2010	158	20	15	167	8	15	2.7%	−0.57 [−1.31, 0.16]	2010
Patrick – Neary 2008	154	13	6	186	11	8	0.7%	−2.52 [−4.05, −0.99]	2008
Cook 2006	169	14	29	173	16	32	4.8%	−0.26 [−0.77, 0.24]	2006
Cook 2006	163	20	23	173	16	32	4.3%	−0.55 [−1.10, −0.01]	2006
Gallagher 2005	178	5.05	42	182.6	3.2	42	5.5%	−1.08 [−1.54, −0.62]	2005
Lund 2003	160.5	19.7	9	176.8	15.4	9	1.6%	−0.88 [−1.86, 0.10]	2003
Cook 2003	174	14	19	183	11	20	3.3%	−0.70 [−1.35, −0.05]	2003
Georgiades 2003	160	29	12	176	13	11	2.1%	−0.68 [−1.52, 0.17]	2003
Valim 2002	168	13.67	50	174	13.22	50	6.6%	−0.44 [−0.84, −0.05]	2002
Inbar 2001	150	19	12	172	10.5	15	2.0%	−1.44 [−2.30, −0.57]	2001
Bazelman 2001	165	16.2	20	173	13.4	20	3.4%	−0.53 [−1.16, 0.10]	2001
Fulcher 2000	171	18	66	182	14	30	5.8%	−0.65 [−1.09, −0.21]	2000
Rowbottom 1998	160	24	16	175	16	16	2.8%	−0.72 [−1.43, 0.00]	1998
Sisto 1996 S	146	21.4	11	160	14.3	5	1.3%	−0.67 [−1.76, 0.42]	1996
Sisto 1996 M	176	11.6	10	182	10.4	17	2.3%	−0.54 [−1.33, 0.26]	1996
Simms 1994	176	8.5	13	179	13.9	13	2.4%	−0.25 [−1.02, 0.52]	1994
Sargent 2002	183.4	10.37	33	188.9	11.25	33	5.0%	−0.50 [−0.99, −0.01]	
**Total (95% CI)**			**1025**			**813**	**100.0%**	**−0.57 [−0.70, −0.44]**	

Heterogeneity: $Tau^2 = 0.03$; $Chi^2 = 33.85$, df = 25 ($P = 0.11$); $I^2 = 26\%$
Test for overall effect: $Z = 8.53$ ($P < 0.00001$)

Std. Mean Difference
IV, Random, 95% CI

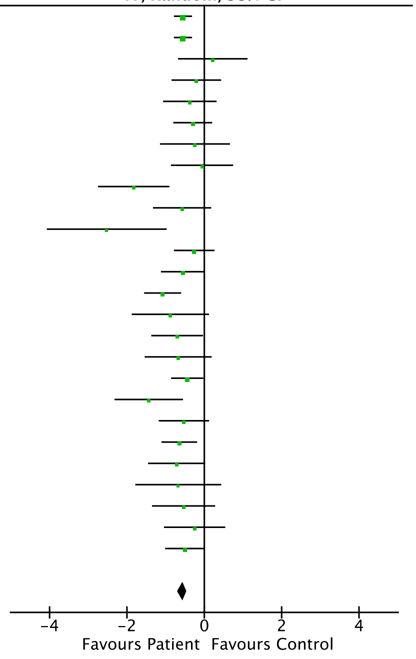

Figure C1. Peak Heart Rate (HR) values for all studies after sensitivity analysis.

Study or Subgroup	Patient Mean	Patient SD	Patient Total	Control Mean	Control SD	Control Total	Weight	Std. Mean Difference IV, Random, 95% CI	Year
**3.3.1 CFS**									
Sargent 2002	183.4	10.3	33	188.9	11.2	33	3.1%	−0.51 [−1.00, −0.01]	
Riley 1990	177	18	13	182	7	13	1.9%	−0.35 [−1.13, 0.42]	1990
Simms 1994	176	8.5	13	179	13.9	13	1.9%	−0.25 [−1.02, 0.52]	1994
Sisto 1996 M	176	11.6	10	182	10.4	17	1.8%	−0.54 [−1.33, 0.26]	1996
Sisto 1996 S	146	21.4	11	160	14.3	5	1.2%	−0.67 [−1.76, 0.42]	1996
Rowbottom 1998	160	24	16	175	16	16	2.1%	−0.72 [−1.43, 0.00]	1998
De Becker 2000 M	167	12.5	157	175	10.2	163	4.5%	−0.70 [−0.93, −0.48]	2000
De Becker 2000 S	151	24.7	427	171	18.5	204	4.8%	−0.87 [−1.05, −0.70]	2000
Fulcher 2000	171	18	66	182	14	30	3.3%	−0.65 [−1.09, −0.21]	2000
Bazelman 2001	165	16.2	20	173	13.4	20	2.4%	−0.53 [−1.16, 0.10]	2001
Inbar 2001	150	19	12	172	10.5	15	1.7%	−1.44 [−2.30, −0.57]	2001
Farquhar 2002	169	20.6	17	176	12.3	17	2.2%	−0.40 [−1.08, 0.28]	2002
Cook 2003	174	14	19	183	11	20	2.4%	−0.70 [−1.35, −0.05]	2003
Georgiades 2003	160	29	12	176	13	11	1.7%	−0.68 [−1.52, 0.17]	2003
Gallagher 2005	178	5.05	42	182.6	3.2	42	3.2%	−1.08 [−1.54, −0.62]	2005
Cook 2006	163	20	23	173	16	32	2.8%	−0.55 [−1.10, −0.01]	2006
Patrick – Neary 2008	154	13	6	186	11	8	0.7%	−2.52 [−4.05, −0.99]	2008
Valkeinenen 2008	158	7.9	23	177.3	3.39	11	1.3%	−2.76 [−3.77, −1.76]	2008
Suarez 2010	132	25.1	44	164.6	17.2	25	2.8%	−1.43 [−1.98, −0.88]	2010
Vermeulen 2010	158	20	15	167	8	15	2.1%	−0.57 [−1.31, 0.16]	2010
Cook 2012 S	101	9.6	8	105	18.6	13	1.6%	−0.24 [−1.13, 0.64]	2012
Icksman 2013	145.1	22.4	31	164.7	7	31	2.8%	−1.17 [−1.71, −0.63]	2013
Strahler 2013	159.7	20.5	21	162.8	1.5	20	2.5%	−0.21 [−0.82, 0.41]	2013
Vermeulen 2014	157.9	19.3	203	163.6	15.1	18	3.1%	−0.30 [−0.78, 0.18]	2014
Oosterwijick 2017	140	9	20	142	10	20	2.5%	−0.21 [−0.83, 0.42]	2017
Pieroni – Andrade 2017	131	18	24	148	12	20	2.4%	−1.07 [−1.71, −0.43]	2017
Nelson 2019	170	10	10	167	15.7	10	1.6%	0.22 [−0.66, 1.10]	2019
Hodges 2020	162	14	16	165	18	16	2.2%	−0.18 [−0.88, 0.51]	2020
Van Oosterwijck 2021	140	9	20	142	10	20	2.5%	−0.21 [−0.83, 0.42]	2021
Cook 2022	156	20.2	178	166.5	17.6	169	4.6%	−0.55 [−0.77, −0.34]	2022
**Subtotal (95% CI)**			**1510**			**1047**	**73.8%**	**−0.68 [−0.83, −0.53]**	
Heterogeneity: $Tau^2 = 0.08$; $Chi^2 = 65.31$, df = 29 (P = 0.0001); $I^2 = 56\%$									
Test for overall effect: Z = 8.82 (P < 0.00001)									
**3.3.2 FMS**									
Valim 2002	168	13.6	50	174	13.2	50	3.6%	−0.44 [−0.84, −0.05]	2002
Lund 2003	160.5	19.7	9	176.8	15.4	9	1.4%	−0.88 [−1.86, 0.10]	2003
Cook 2006	169	14	29	173	16	32	3.0%	−0.26 [−0.77, 0.24]	2006
Hsieh 2010	145.8	16.7	31	158.9	13.7	31	2.9%	−0.85 [−1.37, −0.33]	2010
Da Cunha 2011	148	18.7	14	177	11.2	14	1.6%	−1.83 [−2.73, −0.92]	2011
Cook 2012 S	104	13.3	12	105	18.6	13	1.9%	−0.06 [−0.84, 0.73]	2012
Bardal 2013	165.8	15.5	12	177.8	12.8	12	1.7%	−0.82 [−1.65, 0.02]	2013
Bachassons 2013	152	20	11	172	10	11	1.5%	−1.22 [−2.14, −0.29]	2013
Balbaloglu 2014	166	11	30	165	9	60	3.4%	0.10 [−0.34, 0.54]	2014
Bardal 2015	134	13.7	16	140	17.1	19	2.3%	−0.37 [−1.05, 0.30]	2015
Vincent 2016	165.5	13.8	30	169.6	12.5	30	3.0%	−0.31 [−0.82, 0.20]	2016
**Subtotal (95% CI)**			**244**			**281**	**26.2%**	**−0.54 [−0.82, −0.25]**	
Heterogeneity: $Tau^2 = 0.12$; $Chi^2 = 23.19$, df = 10 (P = 0.01); $I^2 = 57\%$									
Test for overall effect: Z = 3.68 (P = 0.0002)									
**Total (95% CI)**			**1754**			**1328**	**100.0%**	**−0.64 [−0.77, −0.50]**	
Heterogeneity: $Tau^2 = 0.09$; $Chi^2 = 94.73$, df = 40 (P < 0.00001); $I^2 = 58\%$									
Test for overall effect: Z = 9.25 (P < 0.00001)									
Test for subgroup differences: $Chi^2 = 0.71$, df = 1 (P = 0.40), $I^2 = 0\%$									

Std. Mean Difference IV, Random, 95% CI

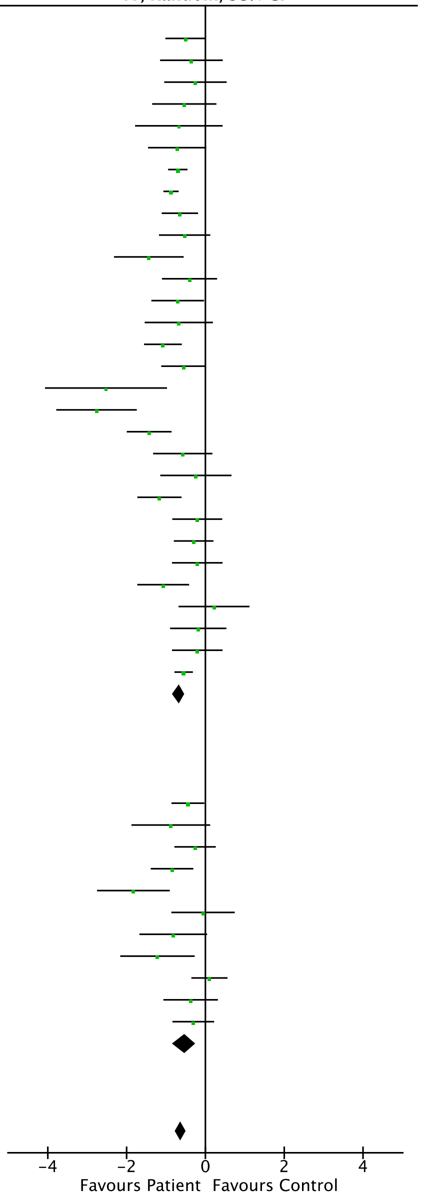

Figure C2. Peak Heart Rate (HR) values for all studies between CFS and FMS patients.

Study or Subgroup	Patient Mean	Patient SD	Patient Total	Control Mean	Control SD	Control Total	Weight	Std. Mean Difference IV, Random, 95% CI	Year
Hodges 2020	21.2	5.6	16	31.1	6.6	16	5.6%	−1.58 [−2.38, −0.77]	2020
Nelson 2019	16	4.1	10	17	2	10	5.1%	−0.30 [−1.18, 0.59]	2019
Bouquet 2019	24	6.3	14	24.3	7.6	11	5.7%	−0.04 [−0.83, 0.75]	2019
Pieroni – Andrade 2017	9.5	2.8	24	12.1	2.2	20	7.0%	−1.00 [−1.64, −0.37]	2017
Vermeulen 2014	11	2.6	203	13.6	3.3	18	8.4%	−0.97 [−1.46, −0.48]	2014
Bardal 2013	1.3	0.3	12	1.7	0.4	12	5.2%	−1.09 [−1.96, −0.22]	2013
Vermeulen 2010	13	3	15	17	4	15	5.8%	−1.10 [−1.88, −0.33]	2010
Hsieh 2010	10.7	2.3	31	13.1	2.9	31	8.0%	−0.91 [−1.43, −0.38]	2010
Van Ness 2007	15	4.9	6	17.5	4.8	6	3.6%	−0.48 [−1.63, 0.68]	2007
Jammes 2005	16	5	26	25	6.6	11	5.6%	−1.60 [−2.40, −0.80]	2005
Lund 2003	17.2	4.3	9	27	4.6	9	3.4%	−2.10 [−3.30, −0.89]	2003
Cook 2003	15	2.7	19	15	2.2	20	7.0%	0.00 [−0.63, 0.63]	2003
Valim 2002	16.3	2.9	50	18.7	3.8	50	9.2%	−0.70 [−1.11, −0.30]	2002
Inbar 2001	14	3.1	12	19	3.5	15	5.2%	−1.46 [−2.32, −0.59]	2001
Sisto 1996	15.5	0.8	21	16.4	0.7	22	6.8%	−1.18 [−1.83, −0.52]	1996
Sargent 2002	17.7	4.8	33	18.8	6.2	33	8.4%	−0.20 [−0.68, 0.29]	
**Total (95% CI)**			**501**			**299**	**100.0%**	**−0.86 [−1.13, −0.60]**	

Heterogeneity: $Tau^2 = 0.15$; $Chi^2 = 34.85$, df = 15 (P = 0.003); $I^2 = 57\%$
Test for overall effect: Z = 6.46 (P < 0.00001)

Std. Mean Difference IV, Random, 95% CI

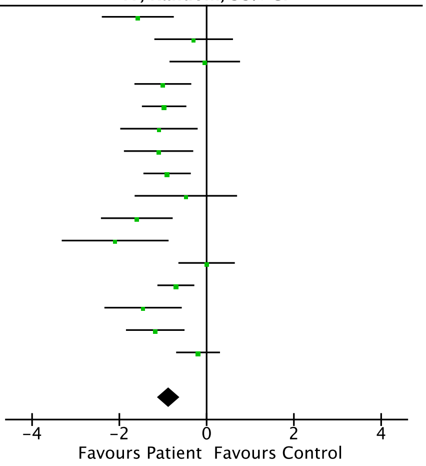

Figure D. Anaerobic Threshold (AT) values for all studies included in the analysis.

Study or Subgroup	Patient Mean	Patient SD	Patient Total	Control Mean	Control SD	Control Total	Weight	Std. Mean Difference IV, Random, 95% CI
Lund 2003	17.2	4.3	9	27	4.6	9	5.3%	−2.10 [−3.30, −0.89]
Jammes 2005	16	5	26	25	6.6	11	8.2%	−1.60 [−2.40, −0.80]
Inbar 2001	14	3.1	12	19	3.5	15	7.6%	−1.46 [−2.32, −0.59]
Sisto 1996	15.5	0.8	21	16.4	0.7	22	9.7%	−1.18 [−1.83, −0.52]
Vermeulen 2010	13	3	15	17	4	15	8.5%	−1.10 [−1.88, −0.33]
Vermeulen 2014	11	2.6	203	13.6	3.3	18	11.4%	−0.97 [−1.46, −0.48]
Valim 2002	16.3	2.9	50	18.7	3.8	50	12.3%	−0.70 [−1.11, −0.30]
Nelson 2019	16	4.1	10	17	2	10	7.5%	−0.30 [−1.18, 0.59]
Sargent 2002	17.7	4.8	33	18.8	6.2	33	11.4%	−0.20 [−0.68, 0.29]
Bouquet 2019	24	6.3	14	24.3	7.6	11	8.3%	−0.04 [−0.83, 0.75]
Cook 2003	15	2.7	19	15	2.2	20	9.9%	0.00 [−0.63, 0.63]
**Total (95% CI)**			**412**			**214**	**100.0%**	**−0.80 [−1.15, −0.46]**

Heterogeneity: $Tau^2 = 0.21$; $Chi^2 = 29.48$, df = 10 (P = 0.001); $I^2 = 66\%$
Test for overall effect: Z = 4.57 (P < 0.00001)

Std. Mean Difference IV, Random, 95% CI

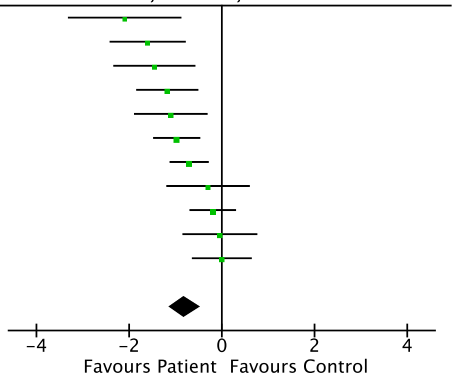

Figure D1. Anaerobic Threshold (AT) values for all studies after sensitivity analysis.

Study or Subgroup	Patient Mean	Patient SD	Patient Total	Control Mean	Control SD	Control Total	Weight	Std. Mean Difference IV, Random, 95% CI
**4.3.1 CFS**								
Jammes 2005	16	5	26	25	6.6	11	5.6%	−1.60 [−2.40, −0.80]
Hodges 2020	21.2	5.6	16	31.1	6.6	16	5.6%	−1.58 [−2.38, −0.77]
Inbar 2001	14	3.1	12	19	3.5	15	5.2%	−1.46 [−2.32, −0.59]
Sisto 1996	15.5	0.8	21	16.4	0.7	22	6.8%	−1.18 [−1.83, −0.52]
Vermeulen 2010	13	3	15	17	4	15	5.8%	−1.10 [−1.88, −0.33]
Vermeulen 2014	11	2.6	203	13.6	3.3	18	8.4%	−0.97 [−1.46, −0.48]
Van Ness 2007	15	4.9	6	17.5	4.8	6	3.6%	−0.48 [−1.63, 0.68]
Nelson 2019	16	4.1	10	17	2	10	5.1%	−0.30 [−1.18, 0.59]
Sargent 2002	17.7	4.8	33	18.8	6.2	33	8.4%	−0.20 [−0.68, 0.29]
Bouquet 2019	24	6.3	14	24.3	7.6	11	5.7%	−0.04 [−0.83, 0.75]
Cook 2003	15	2.7	19	15	2.2	20	7.0%	0.00 [−0.63, 0.63]
**Subtotal (95% CI)**			**375**			**177**	**67.2%**	**−0.80 [−1.16, −0.43]**
Heterogeneity: $Tau^2 = 0.24$; $Chi^2 = 28.94$, df = 10 (P = 0.001); $I^2 = 65\%$								
Test for overall effect: Z = 4.24 (P < 0.0001)								
**4.3.2 FMS**								
Lund 2003	17.2	4.3	9	27	4.6	9	3.4%	−2.10 [−3.30, −0.89]
Bardal 2013	1.3	0.3	12	1.7	0.4	12	5.2%	−1.09 [−1.96, −0.22]
Pieroni – Andrade 2017	9.5	2.8	24	12.1	2.2	20	7.0%	−1.00 [−1.64, −0.37]
Hsieh 2010	10.7	2.3	31	13.1	2.9	31	8.0%	−0.91 [−1.43, −0.38]
Valim 2002	16.3	2.9	50	18.7	3.8	50	9.2%	−0.70 [−1.11, −0.30]
**Subtotal (95% CI)**			**126**			**122**	**32.8%**	**−0.94 [−1.25, −0.63]**
Heterogeneity: $Tau^2 = 0.02$; $Chi^2 = 4.96$, df = 4 (P = 0.29); $I^2 = 19\%$								
Test for overall effect: Z = 5.98 (P < 0.00001)								
**Total (95% CI)**			**501**			**299**	**100.0%**	**−0.86 [−1.13, −0.60]**

Heterogeneity: $Tau^2 = 0.15$; $Chi^2 = 34.85$, df = 15 (P = 0.003); $I^2 = 57\%$
Test for overall effect: Z = 6.46 (P < 0.00001)
Test for subgroup differences: $Chi^2 = 0.37$, df = 1 (P = 0.54), $I^2 = 0\%$

Std. Mean Difference IV, Random, 95% CI

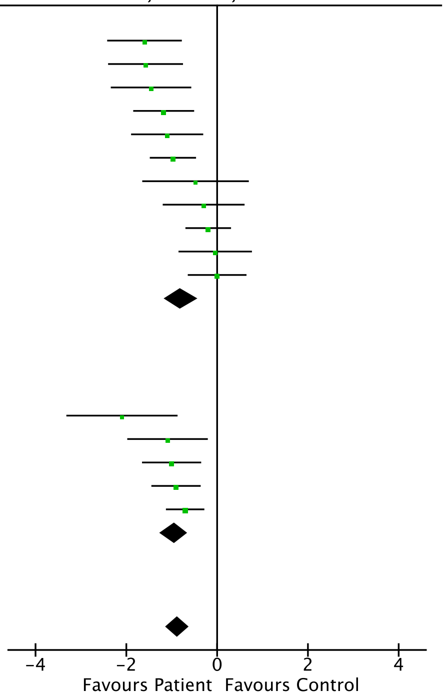

Figure D2. Anaerobic Threshold (AT) values for all studies between FMS and CFS

	Patient			Control				Std. Mean Difference	
Study or Subgroup	Mean	SD	Total	Mean	SD	Total	Weight	IV, Random, 95% CI	Year
Wahlen 2022	154	101.5	40	234	41.5	24	2.0%	−0.94 [−1.47, −0.40]	2022
Wahlen 2022	299	128	40	399.5	90.3	24	2.0%	−0.86 [−1.39, −0.33]	2022
Kapuczinski 2022	18	8	45	30	6	39	2.0%	−1.66 [−2.16, −1.16]	2022
Wahlen 2022	11.8	7.9	40	18.4	7.8	24	2.0%	−0.83 [−1.36, −0.30]	2022
Berardi 2021	38	12.2	47	40	12.2	47	2.1%	−0.16 [−0.57, 0.24]	2021
Berardi 2021	27.7	7.9	47	29.2	8.4	47	2.1%	−0.18 [−0.59, 0.22]	2021
Jakel 2021	31.4	8.8	105	37.1	8.8	66	2.2%	−0.64 [−0.96, −0.33]	2021
Jakel 2021	264	108	105	333	82	66	2.2%	−0.70 [−1.01, −0.38]	2021
Salaffi 2020	14.8	4.74	110	19.9	5.39	11	1.8%	−1.06 [−1.69, −0.42]	2020
Sempere Rubio 2019	82.2	56.8	123	55.2	49.8	100	2.2%	0.50 [0.23, 0.77]	2019
Sempere Rubio 2019	52.5	18.7	123	95.9	25.7	100	2.2%	−1.96 [−2.28, −1.63]	2019
Villafaina 2018	24.04	4.7	30	25.9	3.7	31	2.0%	−0.44 [−0.94, 0.07]	2018
Ceron Lorente 2018	21.7	13.5	34	38.5	12.9	22	1.9%	−1.25 [−1.84, −0.66]	2018
Larsson 2018	152.8	65.3	118	233.3	56.9	93	2.2%	−1.30 [−1.60, −1.00]	2018
Larsson 2018	120.1	49.3	118	185.3	52.5	93	2.2%	−1.28 [−1.58, −0.98]	2018
Larsson 2018	322.8	108.9	118	401.3	81.7	93	2.2%	−0.80 [−1.08, −0.52]	2018
Nacul 2018	27.1	12.2	136	36.2	14.1	216	2.3%	−0.68 [−0.90, −0.46]	2018
Sener 2016	19.6	5.6	39	24.5	4.5	40	2.0%	−0.96 [−1.42, −0.49]	2016
Gerdle 2016 HG	12.8	4.6	29	17	4.5	28	1.9%	−0.91 [−1.46, −0.36]	2016
Gerdle 2016 Lext	340.8	98.1	29	370.1	85.8	28	2.0%	−0.31 [−0.84, 0.21]	2016
Umeda 2015	22.5	5.3	14	28.6	6.8	14	1.6%	−0.97 [−1.76, −0.18]	2015
Aparicio 2015	19.7	7.6	487	19.6	8.1	250	2.3%	0.01 [−0.14, 0.17]	2015
Gomez – Cabello 2015	17.1	9.6	28	42.2	13	22	1.7%	−2.20 [−2.92, −1.49]	2015
Gomez – Cabello 2015	18.7	5.9	28	26.3	5	22	1.8%	−1.35 [−1.98, −0.73]	2015
Koklu 2015	22.2	4.9	25	27.3	5.2	23	1.9%	−0.99 [−1.60, −0.39]	2015
La Torre – Roman 2015	19.6	7.4	492	29.4	7.8	279	2.3%	−1.30 [−1.46, −1.14]	2015
Icksman 2014	28.9	7.74	30	33.7	10.1	30	2.0%	−0.53 [−1.04, −0.01]	2014
Icksman 2014	32.8	7	30	33.7	10.1	30	2.0%	−0.10 [−0.61, 0.40]	2014
Lee 2014	13.34	5.2	29	20.1	2.66	9	1.6%	−1.39 [−2.21, −0.57]	2014
Srikuea 2013	114.5	20.1	11	131.5	30.5	11	1.5%	−0.63 [−1.49, 0.23]	2013
Aparicio 2013	19.6	7.7	208	26.5	11.6	108	2.2%	−0.75 [−0.99, −0.51]	2013
Bachassons 2013	102	18	11	120	24	11	1.5%	−0.82 [−1.69, 0.06]	2013
Icksman 2013	50.9	13.3	30	58.9	20	13	1.8%	−0.50 [−1.16, 0.16]	2013
Gerdle 2013	235	81	19	312	89	14	1.7%	−0.89 [−1.62, −0.16]	2012
Goes 2012	31.14	18.1	16	47.3	20.1	16	1.7%	−0.82 [−1.55, −0.10]	2012
Klaver-Kol 2012	92.9	20	13	114.2	17.9	14	1.6%	−1.09 [−1.91, −0.27]	2012
Aparicio 2011	19.3	6.5	81	27.9	4.1	44	2.1%	−1.48 [−1.89, −1.07]	2011
Aparicio 2010	32.9	10.4	20	39.8	8.9	60	2.0%	−0.74 [−1.26, −0.22]	2010
Giske 2008	407	118	19	574	99	19	1.7%	−1.50 [−2.23, −0.77]	2008
Valkeinenen 2008	1,285	248.1	23	1,931	385.1	11	1.5%	−2.12 [−3.01, −1.22]	2008
Valkeinenen 2008	414	68.1	13	501.7	100.9	11	1.5%	−1.00 [−1.86, −0.14]	2008
Panton 2006	39	11	29	40	12	12	1.8%	−0.09 [−0.76, 0.59]	2006
Semionow 2004	305.8	150.4	8	412	103.6	8	1.4%	−0.78 [−1.81, 0.25]	2004
Sahin 2004	20.1	5.9	41	23	4.8	40	2.1%	−0.53 [−0.98, −0.09]	2004
Maquet 2002	19.7	9	16	27.1	7	85	1.9%	−1.00 [−1.55, −0.45]	2002
Blackwood 1998	33.1	8.5	10	35.5	11.4	10	1.5%	−0.23 [−1.11, 0.65]	2001
Elert 2001	29.9	8.5	22	67.3	12.1	22	1.4%	−3.51 [−4.48, −2.54]	2001
Fulcher 2000	310	115.2	66	441	119.8	30	2.0%	−1.11 [−1.57, −0.65]	2000
Sacco 1999	159.5	23	10	180.5	15.5	10	1.4%	−1.03 [−1.97, −0.08]	1999
Simms 1994	50.1	22	13	55.8	17.5	13	1.7%	−0.28 [−1.05, 0.50]	1994
Simms 1994	20.9	5.7	13	21.4	3.3	13	1.7%	−0.10 [−0.87, 0.67]	1994
Nordenskiold 1993	110	50.7	14	273.7	67.5	105	1.8%	−2.47 [−3.11, −1.83]	1993
Mengshoel 1990	58	22	26	97	12	26	1.8%	−2.17 [−2.86, −1.47]	1990
**Total (95% CI)**			**3371**			**2577**	**100.0%**	**−0.93 [−1.12, −0.75]**	

Heterogeneity: $Tau^2 = 0.38$; $Chi^2 = 486.46$, df = 52 ($P < 0.00001$); $I^2 = 89\%$

Test for overall effect: Z = 9.94 ($P < 0.00001$)

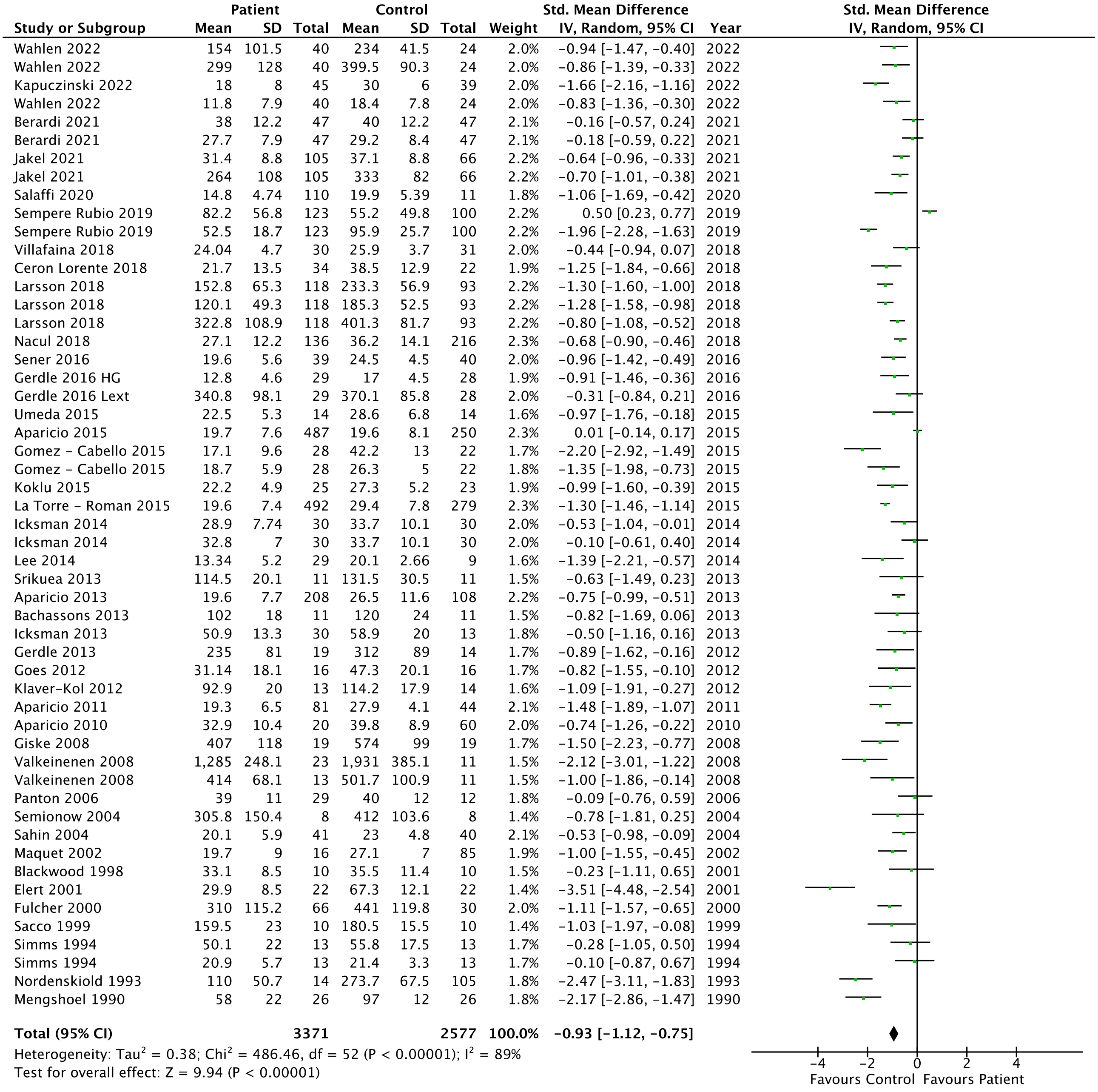

Figure E. Maximal Voluntary Contraction (MVC) values for all studies included in the analysis.

	Patient			Control				Std. Mean Difference	
**Study or Subgroup**	**Mean**	**SD**	**Total**	**Mean**	**SD**	**Total**	**Weight**	**IV, Random, 95% CI**	**Year**
Wahlen 2022	154	101.5	40	234	41.5	24	4.3%	−0.94 [−1.47, −0.40]	2022
Wahlen 2022	299	128	40	399.5	90.3	24	4.4%	−0.86 [−1.39, −0.33]	2022
Wahlen 2022	11.8	7.9	40	18.4	7.8	24	4.4%	−0.83 [−1.36, −0.30]	2022
Jakel 2021	31.4	8.8	105	37.1	8.8	66	7.2%	−0.64 [−0.96, −0.33]	2021
Jakel 2021	264	108	105	333	82	66	7.2%	−0.70 [−1.01, −0.38]	2021
Berardi 2021	38	12.2	47	40	12.2	47	5.8%	−0.16 [−0.57, 0.24]	2021
Berardi 2021	27.7	7.9	47	29.2	8.4	47	5.8%	−0.18 [−0.59, 0.22]	2021
Gerdle 2016 Lext	340.8	98.1	29	370.1	85.8	28	4.4%	−0.31 [−0.84, 0.21]	2016
Sener 2016	19.6	5.6	39	24.5	4.5	40	5.0%	−0.96 [−1.42, −0.49]	2016
Gerdle 2016 HG	12.8	4.6	29	17	4.5	28	4.2%	−0.91 [−1.46, −0.36]	2016
Umeda 2015	22.5	5.3	14	28.6	6.8	14	2.5%	−0.97 [−1.76, −0.18]	2015
Icksman 2014	32.8	7	30	33.7	10.1	30	4.6%	−0.10 [−0.61, 0.40]	2014
Icksman 2014	28.9	7.74	30	33.7	10.1	30	4.5%	−0.53 [−1.04, −0.01]	2014
Icksman 2013	50.9	13.3	30	58.9	20	13	3.3%	−0.50 [−1.16, 0.16]	2013
Srikuea 2013	114.5	20.1	11	131.5	30.5	11	2.2%	−0.63 [−1.49, 0.23]	2013
Klaver−Kol 2012	92.9	20	13	114.2	17.9	14	2.4%	−1.09 [−1.91, −0.27]	2012
Goes 2012	31.14	18.1	16	47.3	20.1	16	2.8%	−0.82 [−1.55, −0.10]	2012
Giske 2008	407	118	19	574	99	19	2.8%	−1.50 [−2.23, −0.77]	2008
Panton 2006	39	11	29	40	12	12	3.2%	−0.09 [−0.76, 0.59]	2006
Semionow 2004	305.8	150.4	8	412	103.6	8	1.6%	−0.78 [−1.81, 0.25]	2004
Sahin 2004	20.1	5.9	41	23	4.8	40	5.3%	−0.53 [−0.98, −0.09]	2004
Fulcher 2000	310	115.2	66	441	119.8	30	5.1%	−1.11 [−1.57, −0.65]	2000
Sacco 1999	159.5	23	10	180.5	15.5	10	1.9%	−1.03 [−1.97, −0.08]	1999
Simms 1994	50.1	22	13	55.8	17.5	13	2.6%	−0.28 [−1.05, 0.50]	1994
Simms 1994	20.9	5.7	13	21.4	3.3	13	2.6%	−0.10 [−0.87, 0.67]	1994
**Total (95% CI)**			**864**			**667**	**100.0%**	**−0.63 [−0.78, −0.49]**	

Heterogeneity: $Tau^2 = 0.05$; $Chi^2 = 39.23$, df = 24 (P = 0.03); $I^2 = 39\%$
Test for overall effect: Z = 8.79 (P < 0.00001)

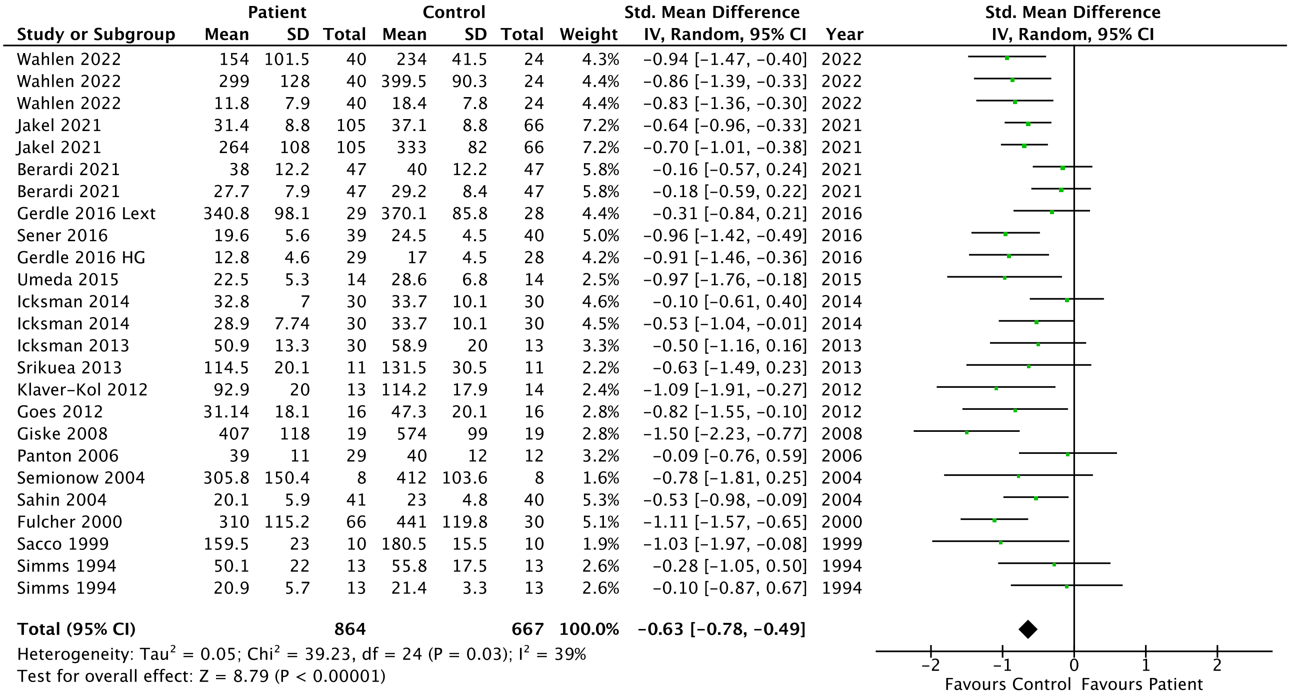

Figure E1. Maximal Voluntary Contraction (MVC) values for all studies after sensitivity analysis.

Study or Subgroup	Patient Mean	Patient SD	Patient Total	Control Mean	Control SD	Control Total	Weight	Std. Mean Difference IV, Random, 95% CI	Year
**9.3.1 CFS**									
Sacco 1999	159.5	23	10	180.5	15.5	10	1.4%	−1.03 [−1.97, −0.08]	1999
Fulcher 2000	310	115.2	66	441	119.8	30	2.0%	−1.11 [−1.57, −0.65]	2000
Blackwood 1998	33.1	8.5	10	35.5	11.4	10	1.5%	−0.23 [−1.11, 0.65]	2001
Semionow 2004	305.8	150.4	8	412	103.6	8	1.4%	−0.78 [−1.81, 0.25]	2004
Icksman 2013	50.9	13.3	30	58.9	20	13	1.8%	−0.50 [−1.16, 0.16]	2013
Nacul 2018	27.1	12.2	136	36.2	14.1	216	2.3%	−0.68 [−0.90, −0.46]	2018
Jakel 2021	31.4	8.8	105	37.1	8.8	66	2.2%	−0.64 [−0.96, −0.33]	2021
Jakel 2021	264	108	105	333	82	66	2.2%	−0.70 [−1.01, −0.38]	2021
**Subtotal (95% CI)**			**470**			**419**	**14.8%**	**−0.71 [−0.85, −0.57]**	
Heterogeneity: $Tau^2 = 0.00$; $Chi^2 = 5.17$, df = 7 (P = 0.64); $I^2 = 0\%$									
Test for overall effect: Z = 9.87 (P < 0.00001)									
**9.3.2 FMS**									
Mengshoel 1990	58	22	26	97	12	26	1.8%	−2.17 [−2.86, −1.47]	1990
Nordenskiold 1993	110	50.7	14	273.7	67.5	105	1.8%	−2.47 [−3.11, −1.83]	1993
Simms 1994	50.1	22	13	55.8	17.5	13	1.7%	−0.28 [−1.05, 0.50]	1994
Simms 1994	20.9	5.7	13	21.4	3.3	13	1.7%	−0.10 [−0.87, 0.67]	1994
Elert 2001	29.9	8.5	22	67.3	12.1	22	1.4%	−3.51 [−4.48, −2.54]	2001
Maquet 2002	19.7	9	16	27.1	7	85	1.9%	−1.00 [−1.55, −0.45]	2002
Sahin 2004	20.1	5.9	41	23	4.8	40	2.1%	−0.53 [−0.98, −0.09]	2004
Panton 2006	39	11	29	40	12	12	1.8%	−0.09 [−0.76, 0.59]	2006
Giske 2008	407	118	19	574	99	19	1.7%	−1.50 [−2.23, −0.77]	2008
Valkeinenen 2008	1,285	248.1	23	1,931	385.1	11	1.5%	−2.12 [−3.01, −1.22]	2008
Valkeinenen 2008	414	68.1	13	501.7	100.9	11	1.5%	−1.00 [−1.86, −0.14]	2008
Aparicio 2010	32.9	10.4	20	39.8	8.9	60	2.0%	−0.74 [−1.26, −0.22]	2010
Aparicio 2011	19.3	6.5	81	27.9	4.1	44	2.1%	−1.48 [−1.89, −1.07]	2011
Goes 2012	31.14	18.1	16	47.3	20.1	16	1.7%	−0.82 [−1.55, −0.10]	2012
Klaver–Kol 2012	92.9	20	13	114.2	17.9	14	1.6%	−1.09 [−1.91, −0.27]	2012
Gerdle 2013	235	81	19	312	89	14	1.7%	−0.89 [−1.62, −0.16]	2012
Bachassons 2013	102	18	11	120	24	11	1.5%	−0.82 [−1.69, 0.06]	2013
Aparicio 2013	19.6	7.7	208	26.5	11.6	108	2.2%	−0.75 [−0.99, −0.51]	2013
Srikuea 2013	114.5	20.1	11	131.5	30.5	11	1.5%	−0.63 [−1.49, 0.23]	2013
Lee 2014	13.34	5.2	29	20.1	2.66	9	1.6%	−1.39 [−2.21, −0.57]	2014
Icksman 2014	32.8	7	30	33.7	10.1	30	2.0%	−0.10 [−0.61, 0.40]	2014
Icksman 2014	28.9	7.74	30	33.7	10.1	30	2.0%	−0.53 [−1.04, −0.01]	2014
La Torre – Roman 2015	19.6	7.4	492	29.4	7.8	279	2.3%	−1.30 [−1.46, −1.14]	2015
Koklu 2015	22.2	4.9	25	27.3	5.2	23	1.9%	−0.99 [−1.60, −0.39]	2015
Gomez – Cabello 2015	17.1	9.6	28	42.2	13	22	1.7%	−2.20 [−2.92, −1.49]	2015
Gomez – Cabello 2015	18.7	5.9	28	26.3	5	22	1.8%	−1.35 [−1.98, −0.73]	2015
Aparicio 2015	19.7	7.6	487	19.6	8.1	250	2.3%	0.01 [−0.14, 0.17]	2015
Umeda 2015	22.5	5.3	14	28.6	6.8	14	1.6%	−0.97 [−1.76, −0.18]	2015
Gerdle 2016 HG	12.8	4.6	29	17	4.5	28	1.9%	−0.91 [−1.46, −0.36]	2016
Gerdle 2016 Lext	340.8	98.1	29	370.1	85.8	28	2.0%	−0.31 [−0.84, 0.21]	2016
Sener 2016	19.6	5.6	39	24.5	4.5	40	2.0%	−0.96 [−1.42, −0.49]	2016
Larsson 2018	152.8	65.3	118	233.3	56.9	93	2.2%	−1.30 [−1.60, −1.00]	2018
Larsson 2018	120.1	49.3	118	185.3	52.5	93	2.2%	−1.28 [−1.58, −0.98]	2018
Larsson 2018	322.8	108.9	118	401.3	81.7	93	2.2%	−0.80 [−1.08, −0.52]	2018
Ceron Lorente 2018	21.7	13.5	34	38.5	12.9	22	1.9%	−1.25 [−1.84, −0.66]	2018
Villafaina 2018	24.04	4.7	30	25.9	3.7	31	2.0%	−0.44 [−0.94, 0.07]	2018
Sempere Rubio 2019	52.5	18.7	123	95.9	25.7	100	2.2%	−1.96 [−2.28, −1.63]	2019
Sempere Rubio 2019	82.2	56.8	123	55.2	49.8	100	2.2%	0.50 [0.23, 0.77]	2019
Salaffi 2020	14.8	4.74	110	19.9	5.39	11	1.8%	−1.06 [−1.69, −0.42]	2020
Berardi 2021	27.7	7.9	47	29.2	8.4	47	2.1%	−0.18 [−0.59, 0.22]	2021
Berardi 2021	38	12.2	47	40	12.2	47	2.1%	−0.16 [−0.57, 0.24]	2021
Wahlen 2022	154	101.5	40	234	41.5	24	2.0%	−0.94 [−1.47, −0.40]	2022
Wahlen 2022	299	128	40	399.5	90.3	24	2.0%	−0.86 [−1.39, −0.33]	2022
Wahlen 2022	11.8	7.9	40	18.4	7.8	24	2.0%	−0.83 [−1.36, −0.30]	2022
Kapuczinski 2022	18	8	45	30	6	39	2.0%	−1.66 [−2.16, −1.16]	2022
**Subtotal (95% CI)**			**2901**			**2158**	**85.2%**	**−0.98 [−1.19, −0.76]**	
Heterogeneity: $Tau^2 = 0.46$; $Chi^2 = 479.95$, df = 44 (P < 0.00001); $I^2 = 91\%$									
Test for overall effect: Z = 8.85 (P < 0.00001)									
**Total (95% CI)**			**3371**			**2577**	**100.0%**	**−0.93 [−1.12, −0.75]**	
Heterogeneity: $Tau^2 = 0.38$; $Chi^2 = 486.46$, df = 52 (P < 0.00001); $I^2 = 89\%$									
Test for overall effect: Z = 9.94 (P < 0.00001)									
Test for subgroup differences: $Chi^2 = 4.29$, df = 1 (P = 0.04), $I^2 = 76.7\%$									

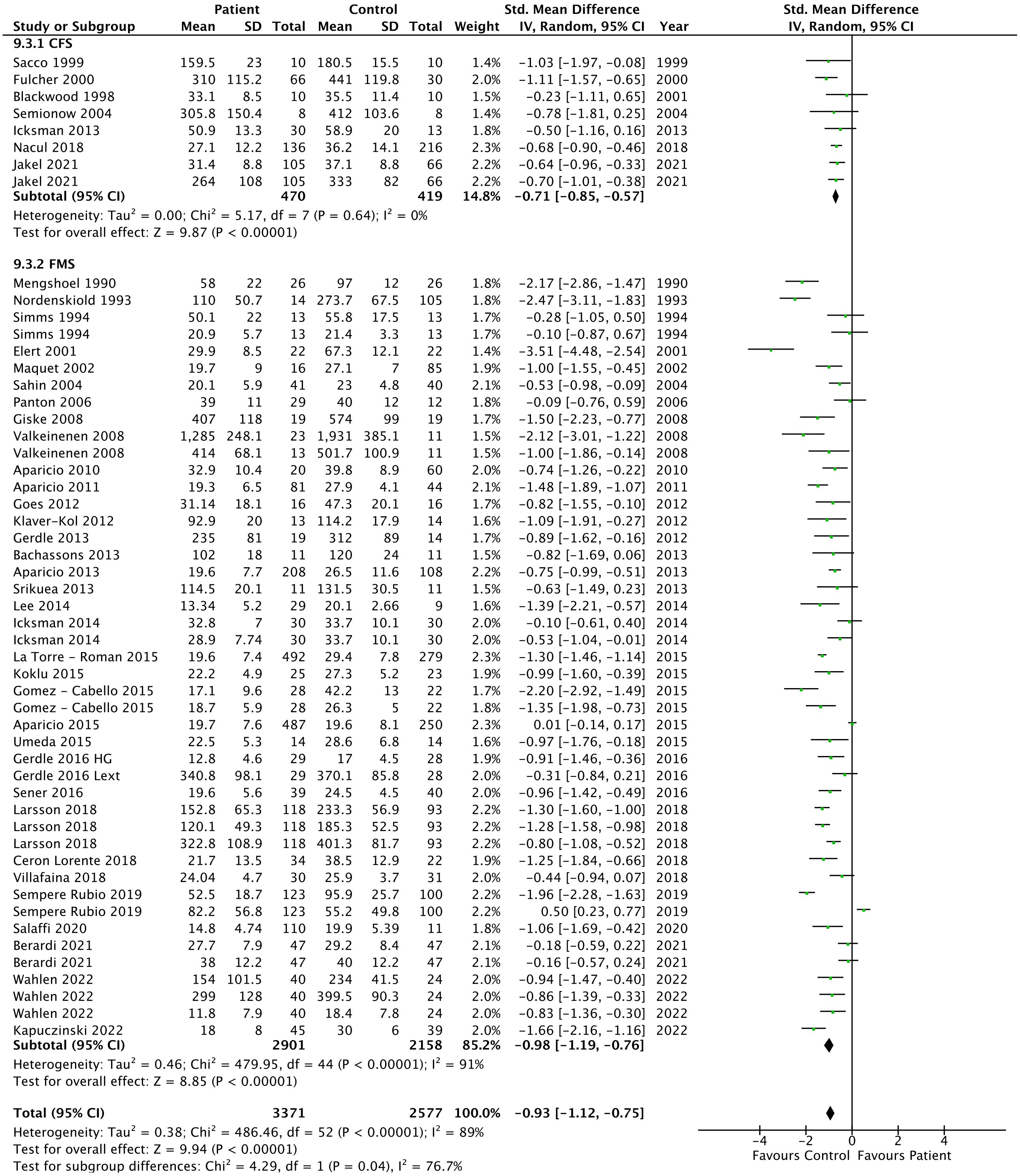

Figure E2. Maximal Voluntary Contraction (MVC) values for all CFS and FMS patients.

Study or Subgroup	Patient Mean	Patient SD	Patient Total	Control Mean	Control SD	Control Total	Weight	Std. Mean Difference IV, Random, 95% CI	Year
Berardi 2021	93.5	8.7	47	93.9	8	47	20.6%	−0.05 [−0.45, 0.36]	2021
Goes 2016	89.5	12.4	29	97.8	4.3	30	17.3%	−0.89 [−1.43, −0.35]	2016
Srikuea 2013	0.99	0.01	11	0.99	0.02	11	11.3%	0.00 [−0.84, 0.84]	2013
Bachassons 2013	93.5	3	11	93.1	3.4	11	11.3%	0.12 [−0.72, 0.96]	2013
Norregard 1995	79	12	15	88	9	14	12.5%	−0.82 [−1.58, −0.06]	1995
Norregard 1994	75	4	20	79	6	21	15.0%	−0.77 [−1.40, −0.13]	1994
Lloyd 1991	93	7.8	12	90.9	9.5	13	12.1%	0.23 [−0.56, 1.02]	1991
**Total (95% CI)**			**145**			**147**	**100.0%**	**−0.34 [−0.70, 0.03]**	

Heterogeneity: $Tau^2 = 0.13$; $Chi^2 = 13.09$, df = 6 (P = 0.04); $I^2 = 54\%$
Test for overall effect: Z = 1.82 (P = 0.07)

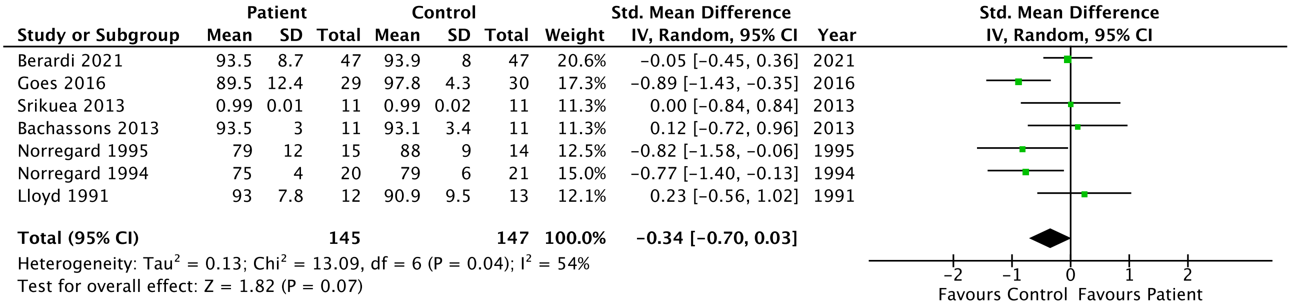

Figure F. Voluntary Activation values for all studies included in the analysis.

Study or Subgroup	Patient Mean	Patient SD	Patient Total	Control Mean	Control SD	Control Total	Weight	Std. Mean Difference IV, Random, 95% CI	Year
**10.3.1 CFS**									
Lloyd 1991	93	7.8	12	90.9	9.5	13	12.1%	0.23 [−0.56, 1.02]	1991
**Subtotal (95% CI)**			**12**			**13**	**12.1%**	**0.23 [−0.56, 1.02]**	
Heterogeneity: Not applicable									
Test for overall effect: Z = 0.58 (P = 0.56)									
**10.3.2 FMS**									
Norregard 1994	75	4	20	79	6	21	15.0%	−0.77 [−1.40, −0.13]	1994
Norregard 1995	79	12	15	88	9	14	12.5%	−0.82 [−1.58, −0.06]	1995
Bachassons 2013	93.5	3	11	93.1	3.4	11	11.3%	0.12 [−0.72, 0.96]	2013
Srikuea 2013	0.99	0.01	11	0.99	0.02	11	11.3%	0.00 [−0.84, 0.84]	2013
Goes 2016	89.5	12.4	29	97.8	4.3	30	17.3%	−0.89 [−1.43, −0.35]	2016
Berardi 2021	93.5	8.7	47	93.9	8	47	20.6%	−0.05 [−0.45, 0.36]	2021
**Subtotal (95% CI)**			**133**			**134**	**87.9%**	**−0.42 [−0.80, −0.03]**	
Heterogeneity: $Tau^2 = 0.12$; $Chi^2 = 10.90$, df = 5 (P = 0.05); $I^2 = 54\%$									
Test for overall effect: Z = 2.13 (P = 0.03)									
**Total (95% CI)**			**145**			**147**	**100.0%**	**−0.34 [−0.70, 0.03]**	

Heterogeneity: $Tau^2 = 0.13$; $Chi^2 = 13.09$, df = 6 (P = 0.04); $I^2 = 54\%$
Test for overall effect: Z = 1.82 (P = 0.07)
Test for subgroup differences: $Chi^2 = 2.12$, df = 1 (P = 0.15), $I^2 = 52.8\%$

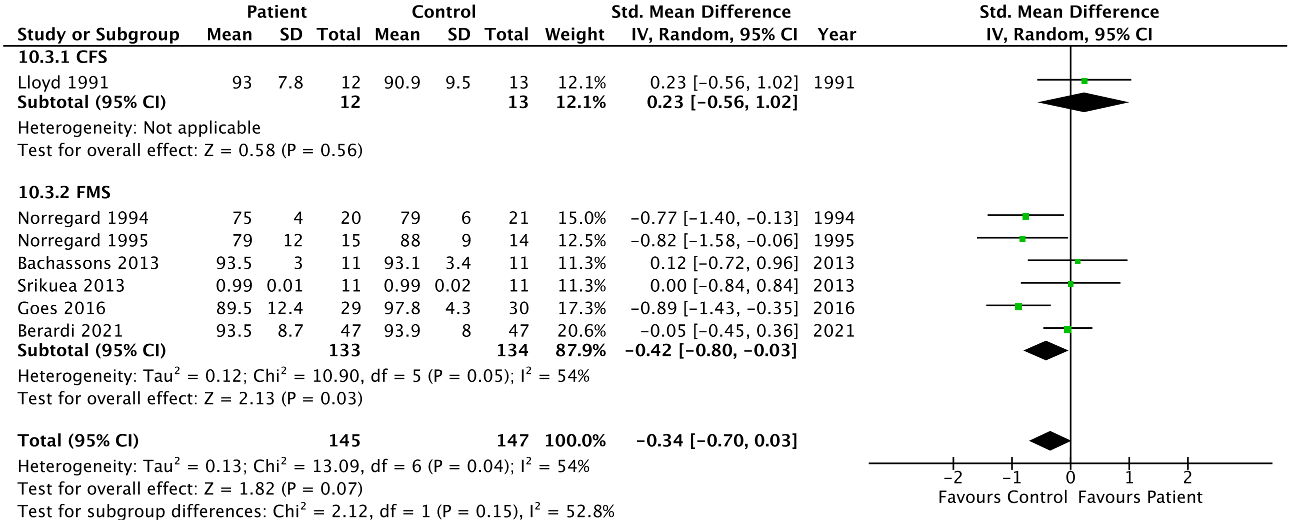

Figure F1. Voluntary Activation values for CFS and FMS patients

Study or Subgroup	Patient Mean	Patient SD	Patient Total	Control Mean	Control SD	Control Total	Weight	Std. Mean Difference IV, Random, 95% CI	Year
Berardi 2021	−9.8	13.8	47	−4	6.7	47	5.4%	−0.53 [−0.94, −0.12]	2021
Jakel 2021	−1.25	0.22	105	−1.1	0.05	66	5.6%	−0.85 [−1.17, −0.53]	2021
Jakel 2021	−1.23	0.11	105	−1.12	0.06	66	5.6%	−1.17 [−1.50, −0.83]	2021
Berardi 2021	−6.5	10.2	47	−6.5	10.2	47	5.4%	0.00 [−0.40, 0.40]	2021
Tavares 2020	−31.5	9.1	40	−28.1	8.9	20	5.1%	−0.37 [−0.91, 0.17]	2020
Tavares 2020	−30.4	10.57	40	−31.1	10.2	20	5.1%	0.07 [−0.47, 0.60]	2020
Srikuea 2013	−30.3	4.9	11	−27.7	10.5	10	4.1%	−0.31 [−1.17, 0.55]	2013
Gerdle 2013	184	65	19	254	75	14	4.5%	−0.98 [−1.72, −0.25]	2012
Maquet 2010	242	79	22	421	174	44	5.0%	−1.18 [−1.73, −0.63]	2010
Maquet 2010	358	328	11	421	174	44	4.7%	−0.29 [−0.96, 0.37]	2010
Maquet 2005	33	7	10	47	12	10	3.7%	−1.36 [−2.36, −0.37]	2005
Maquet 2005	2,278	394	10	3,058	839	15	4.1%	−1.08 [−1.94, −0.21]	2005
Maquet 2002	59	18	16	107	21	85	4.8%	−2.32 [−2.94, −1.69]	2002
Maquet 2002	3,971	1,198	16	6,247	1,588	85	5.0%	−1.47 [−2.04, −0.90]	2002
Elert 2001	16.2	4.7	36	39.6	7.9	27	4.2%	−3.69 [−4.52, −2.86]	2001
Sacco 1999	13.1	10.1	10	18.6	8.2	10	4.0%	−0.57 [−1.47, 0.33]	1999
Borman 1999	37.8	12.1	24	41.8	5.4	15	4.7%	−0.39 [−1.04, 0.26]	1999
Paul 1999	69	4.9	10	70.5	4.1	10	4.0%	−0.32 [−1.20, 0.57]	1999
Norregard 1994	24	19.1	20	29.3	16.6	21	4.9%	−0.29 [−0.91, 0.32]	1994
Mengshoel 1990	152	147	26	413	244	26	4.9%	−1.28 [−1.88, −0.68]	1990
Mengshoel 1990	10	6	26	13	6	26	5.0%	−0.49 [−1.04, 0.06]	1990
**Total (95% CI)**			**651**			**708**	**100.0%**	**−0.88 [−1.19, −0.57]**	

Heterogeneity: $Tau^2 = 0.42$; $Chi^2 = 124.32$, df = 20 (P < 0.00001); $I^2 = 84\%$
Test for overall effect: Z = 5.52 (P < 0.00001)

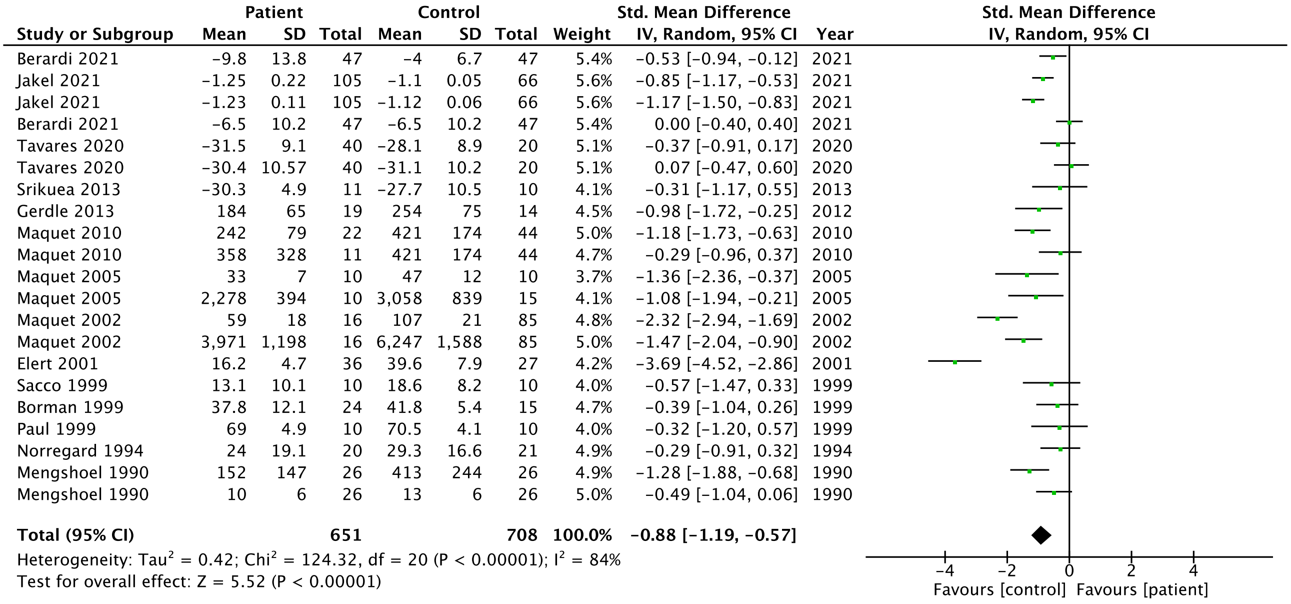

Figure G. Fatigability values for all studies included in the analysis.

Study or Subgroup	Patient Mean	Patient SD	Patient Total	Control Mean	Control SD	Control Total	Weight	Std. Mean Difference IV, Random, 95% CI	Year
Jakel 2021	−1.23	0.11	105	−1.12	0.06	66	13.1%	−1.17 [−1.50, −0.83]	2021
Jakel 2021	−1.25	0.22	105	−1.1	0.05	66	13.3%	−0.85 [−1.17, −0.53]	2021
Berardi 2021	−9.8	13.8	47	−4	6.7	47	12.1%	−0.53 [−0.94, −0.12]	2021
Berardi 2021	−6.5	10.2	47	−6.5	10.2	47	12.2%	0.00 [−0.40, 0.40]	2021
Tavares 2020	−31.5	9.1	40	−28.1	8.9	20	10.3%	−0.37 [−0.91, 0.17]	2020
Tavares 2020	−30.4	10.57	40	−31.1	10.2	20	10.4%	0.07 [−0.47, 0.60]	2020
Srikuea 2013	−30.3	4.9	11	−27.7	10.5	10	6.7%	−0.31 [−1.17, 0.55]	2013
Sacco 1999	13.1	10.1	10	18.6	8.2	10	6.4%	−0.57 [−1.47, 0.33]	1999
Borman 1999	37.8	12.1	24	41.8	5.4	15	8.9%	−0.39 [−1.04, 0.26]	1999
Paul 1999	69	4.9	10	70.5	4.1	10	6.6%	−0.32 [−1.20, 0.57]	1999
**Total (95% CI)**			**439**			**311**	**100.0%**	**−0.47 [−0.77, −0.18]**	

Heterogeneity: $Tau^2 = 0.14$; $Chi^2 = 29.81$, df = 9 (P = 0.0005); $I^2 = 70\%$
Test for overall effect: Z = 3.15 (P = 0.002)

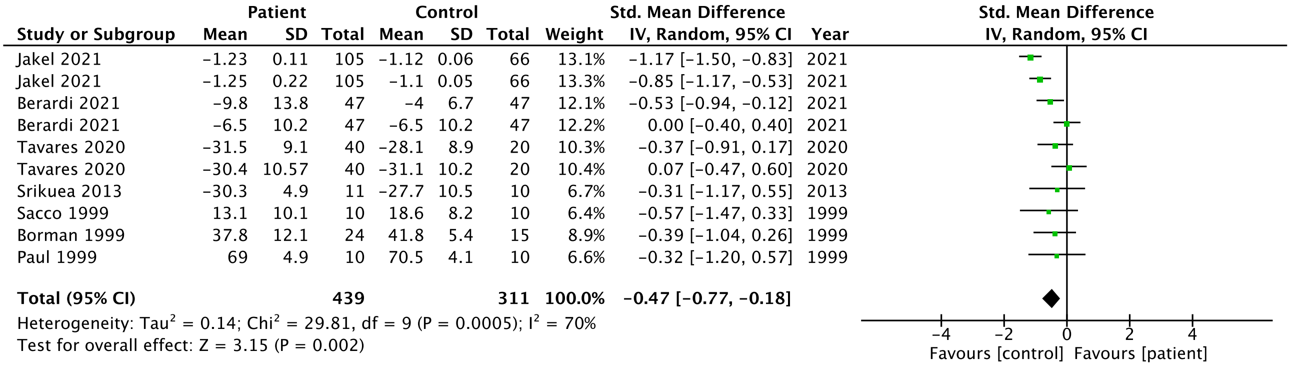

Figure G1. Fatigability values for all studies after sensitivity analysis.

Study or Subgroup	Patient Mean	Patient SD	Patient Total	Control Mean	Control SD	Control Total	Weight	Std. Mean Difference IV, Random, 95% CI	Year
**11.3.1 CFS**									
Sacco 1999	13.1	10.1	10	18.6	8.2	10	4.0%	−0.57 [−1.47, 0.33]	1999
Paul 1999	69	4.9	10	70.5	4.1	10	4.0%	−0.32 [−1.20, 0.57]	1999
Maquet 2010	242	79	22	421	174	44	5.0%	−1.18 [−1.73, −0.63]	2010
Gerdle 2013	184	65	19	254	75	14	4.5%	−0.98 [−1.72, −0.25]	2012
Jakel 2021	−1.25	0.22	105	−1.1	0.05	66	5.6%	−0.85 [−1.17, −0.53]	2021
Jakel 2021	−1.23	0.11	105	−1.12	0.06	66	5.6%	−1.17 [−1.50, −0.83]	2021
**Subtotal (95% CI)**			**271**			**210**	**28.8%**	**−0.97 [−1.17, −0.76]**	
Heterogeneity: $Tau^2 = 0.00$; $Chi^2 = 5.26$, df = 5 (P = 0.38); $I^2 = 5\%$									
Test for overall effect: Z = 9.30 (P < 0.00001)									
**11.3.2 FMS**									
Mengshoel 1990	10	6	26	13	6	26	5.0%	−0.49 [−1.04, 0.06]	1990
Mengshoel 1990	152	147	26	413	244	26	4.9%	−1.28 [−1.88, −0.68]	1990
Norregard 1994	24	19.1	20	29.3	16.6	21	4.9%	−0.29 [−0.91, 0.32]	1994
Borman 1999	37.8	12.1	24	41.8	5.4	15	4.7%	−0.39 [−1.04, 0.26]	1999
Elert 2001	16.2	4.7	36	39.6	7.9	27	4.2%	−3.69 [−4.52, −2.86]	2001
Maquet 2002	59	18	16	107	21	85	4.8%	−2.32 [−2.94, −1.69]	2002
Maquet 2002	3,971	1,198	16	6,247	1,588	85	5.0%	−1.47 [−2.04, −0.90]	2002
Maquet 2005	33	7	10	47	12	10	3.7%	−1.36 [−2.36, −0.37]	2005
Maquet 2005	2,278	394	10	3,058	839	15	4.1%	−1.08 [−1.94, −0.21]	2005
Maquet 2010	358	328	11	421	174	44	4.7%	−0.29 [−0.96, 0.37]	2010
Srikuea 2013	−30.3	4.9	11	−27.7	10.5	10	4.1%	−0.31 [−1.17, 0.55]	2013
Tavares 2020	−31.5	9.1	40	−28.1	8.9	20	5.1%	−0.37 [−0.91, 0.17]	2020
Tavares 2020	−30.4	10.57	40	−31.1	10.2	20	5.1%	0.07 [−0.47, 0.60]	2020
Berardi 2021	−9.8	13.8	47	−4	6.7	47	5.4%	−0.53 [−0.94, −0.12]	2021
Berardi 2021	−6.5	10.2	47	−6.5	10.2	47	5.4%	0.00 [−0.40, 0.40]	2021
**Subtotal (95% CI)**			**380**			**498**	**71.2%**	**−0.89 [−1.34, −0.44]**	
Heterogeneity: $Tau^2 = 0.67$; $Chi^2 = 114.90$, df = 14 (P < 0.00001); $I^2 = 88\%$									
Test for overall effect: Z = 3.91 (P < 0.0001)									
**Total (95% CI)**			**651**			**708**	**100.0%**	**−0.88 [−1.19, −0.57]**	

Heterogeneity: $Tau^2 = 0.42$; $Chi^2 = 124.32$, df = 20 (P < 0.00001); $I^2 = 84\%$
Test for overall effect: Z = 5.52 (P < 0.00001)
Test for subgroup differences: $Chi^2 = 0.09$, df = 1 (P = 0.76), $I^2 = 0\%$

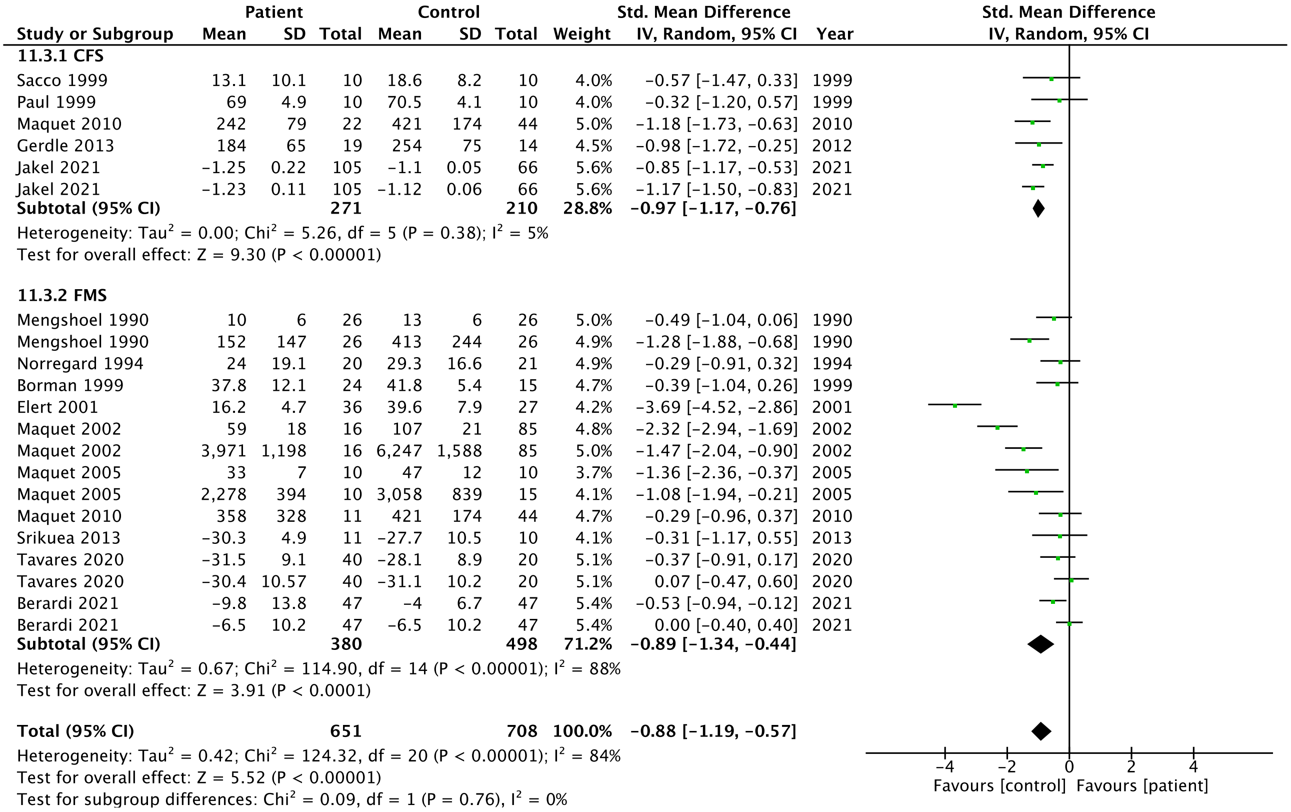

Figure G2. Fatigability values between CFS and FMS patients

Study or Subgroup	Patient Mean	Patient SD	Patient Total	Control Mean	Control SD	Control Total	Weight	Std. Mean Difference IV, Random, 95% CI
Bachassons 2013	539	93	11	650	109	11	16.6%	−1.05 [−1.96, −0.15]
Gerdle 2013	1.04	0.17	19	1.15	0.14	14	26.7%	−0.68 [−1.39, 0.03]
Norregard 1994	164	30	20	181	29	21	34.6%	−0.57 [−1.19, 0.06]
Norregard 1995	58.5	12	13	59	5.7	12	22.0%	−0.05 [−0.84, 0.73]
**Total (95% CI)**			**63**			**58**	**100.0%**	**−0.56 [−0.93, −0.20]**

Heterogeneity: $Tau^2 = 0.00$; $Chi^2 = 2.87$, df = 3 (P = 0.41); $I^2 = 0\%$
Test for overall effect: Z = 3.00 (P = 0.003)

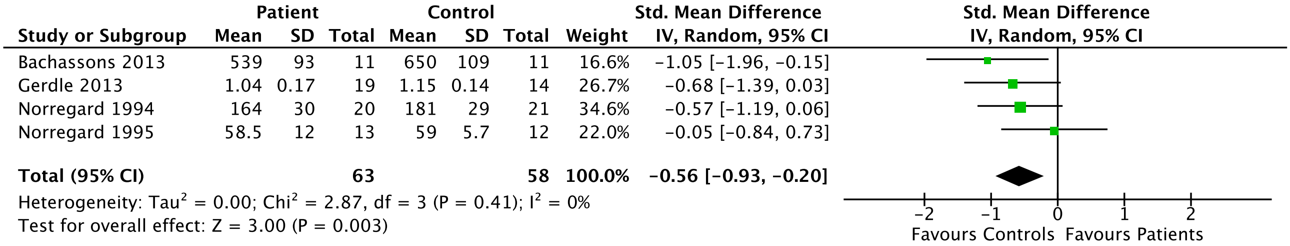

Figure H. Muscle Volume values for all studies included in the analysis.

Study or Subgroup	Experimental Mean	SD	Total	Control Mean	SD	Total	Weight	Std. Mean Difference IV, Random, 95% CI	Year
Kapuczinski 2022	42.4	6.3	45	42.8	6.4	39	7.5%	−0.06 [−0.49, 0.37]	2022
Berardi 2021	44.3	7.5	47	43.7	7.2	47	8.0%	0.08 [−0.32, 0.49]	2021
Andretta 2020	39.6	7.5	53	39.8	6.9	50	8.4%	−0.03 [−0.41, 0.36]	2020
Sener 2016	42.9	5.3	39	43.5	7.5	40	7.3%	−0.09 [−0.53, 0.35]	2016
Vincent 2016	25.8	4.6	19	26	4	19	4.6%	−0.05 [−0.68, 0.59]	2016
Aparicio 2015	22.7	3.3	487	23	3.3	250	14.2%	−0.09 [−0.24, 0.06]	2015
Gomez – Cabello 2015	40.4	7.6	28	38.2	4.4	22	5.4%	0.34 [−0.22, 0.90]	2015
La Torre – Roman 2015	22.9	3.8	492	24.8	5.3	279	14.3%	−0.43 [−0.58, −0.28]	2015
Segura 2014	22.8	3.3	563	23	3.4	249	14.3%	−0.06 [−0.21, 0.09]	2014
Aparicio 2013	24.7	3.8	208	27.4	5.7	108	12.0%	−0.59 [−0.83, −0.36]	2013
Lowe 2006	47.4	6.1	15	46.2	7.4	15	3.8%	0.17 [−0.55, 0.89]	2006
**Total (95% CI)**			**1996**			**1118**	**100.0%**	**−0.14 [−0.30, 0.02]**	

Heterogeneity: $Tau^2 = 0.04$; $Chi^2 = 33.28$, df = 10 (P = 0.0002); $I^2 = 70\%$
Test for overall effect: Z = 1.69 (P = 0.09)

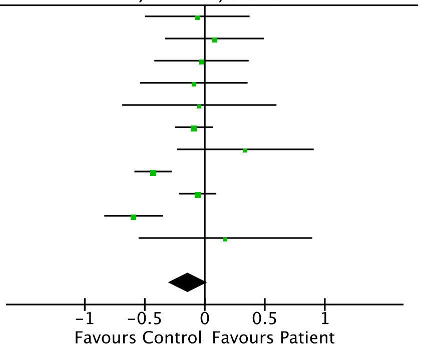

Figure I. Muscle Mass values for all studies included in the analysis.

Study or Subgroup	Experimental Mean	SD	Total	Control Mean	SD	Total	Weight	Std. Mean Difference IV, Random, 95% CI
Sener 2016	42.9	5.3	39	43.5	7.5	40	23.0%	−0.09 [−0.53, 0.35]
Vincent 2016	25.8	4.6	19	26	4	19	11.1%	−0.05 [−0.68, 0.59]
Andretta 2020	39.6	7.5	53	39.8	6.9	50	29.9%	−0.03 [−0.41, 0.36]
Berardi 2021	44.3	7.5	47	43.7	7.2	47	27.3%	0.08 [−0.32, 0.49]
Lowe 2006	47.4	6.1	15	46.2	7.4	15	8.7%	0.17 [−0.55, 0.89]
**Total (95% CI)**			**173**			**171**	**100.0%**	**0.00 [−0.21, 0.21]**

Heterogeneity: $Tau^2 = 0.00$; $Chi^2 = 0.58$, df = 4 (P = 0.97); $I^2 = 0\%$
Test for overall effect: Z = 0.03 (P = 0.98)

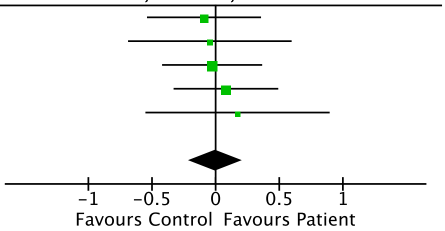

Figure I1. Muscle Mass values after sensitivity analysis for all studies included in the analysis.

Study or Subgroup	Patient Mean	SD	Total	Control Mean	SD	Total	Weight	Std. Mean Difference IV, Random, 95% CI	Year
Kapuczinski 2022	23.8	7.1	45	23.4	7.7	39	6.1%	0.05 [−0.38, 0.48]	2022
Berardi 2021	33.6	12.8	47	28.5	12.2	47	6.5%	0.40 [−0.00, 0.81]	2021
Andretta 2020	39.6	7.5	53	39.8	6.9	50	6.9%	−0.03 [−0.41, 0.36]	2020
Paiva 2017	30.9	4.9	27	31.4	4.7	25	4.5%	−0.10 [−0.65, 0.44]	2017
Acosta Manzano 2017	39.7	6.2	436	37.4	7.3	217	12.6%	0.35 [0.18, 0.51]	2017
Sener 2016	29.8	8.9	39	28.4	8.6	40	5.9%	0.16 [−0.28, 0.60]	2016
Vincent 2016	37.5	7.4	19	35.5	7.6	19	3.5%	0.26 [−0.38, 0.90]	2016
Aparicio 2015	40.1	7.7	487	36.7	7.5	250	12.9%	0.44 [0.29, 0.60]	2015
Gomez – Cabello 2015	28.7	9.3	28	20.1	6.1	22	3.9%	1.05 [0.45, 1.65]	2015
Segura 2014	29.2	10.5	563	25.6	9.2	249	13.0%	0.36 [0.21, 0.51]	2014
Bachassons 2013	32	5	11	32	4	11	2.3%	0.00 [−0.84, 0.84]	2013
Srikuea 2013	38.9	9.1	11	37.1	6.6	11	2.3%	0.22 [−0.62, 1.06]	2013
Aparicio 2013	46.8	5.1	208	42.4	6.3	108	10.4%	0.79 [0.55, 1.03]	2013
Gerdle 2013	9	2.2	19	6.6	2.2	14	2.8%	1.06 [0.32, 1.81]	2012
Lowe 2006	35.9	4.6	15	33.4	6	15	2.9%	0.45 [−0.27, 1.18]	2006
Norregard 1994	1.6	0.6	20	1.6	0.4	21	3.8%	0.00 [−0.61, 0.61]	1994
**Total (95% CI)**			**2028**			**1138**	**100.0%**	**0.36 [0.22, 0.50]**	

Heterogeneity: $Tau^2 = 0.03$; $Chi^2 = 33.33$, df = 15 (P = 0.004); $I^2 = 55\%$
Test for overall effect: Z = 5.16 (P < 0.00001)

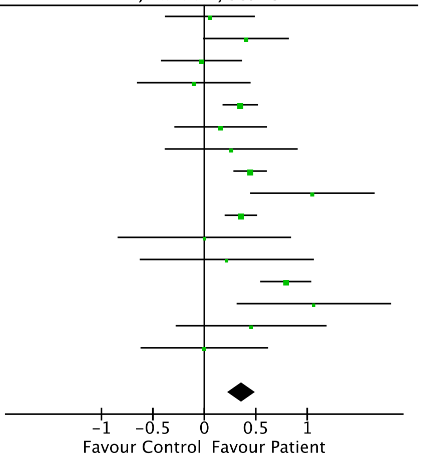

Figure L. Fat Mass values for all studies included in the analysis.

Study or Subgroup	Patient Mean	SD	Total	Control Mean	SD	Total	Weight	Std. Mean Difference IV, Random, 95% CI	Year
Berardi 2021	33.6	12.8	47	28.5	12.2	47	25.4%	0.40 [−0.00, 0.81]	2021
Andretta 2020	39.6	7.5	53	39.8	6.9	50	28.4%	−0.03 [−0.41, 0.36]	2020
Sener 2016	29.8	8.9	39	28.4	8.6	40	21.7%	0.16 [−0.28, 0.60]	2016
Vincent 2016	37.5	7.4	19	35.5	7.6	19	10.4%	0.26 [−0.38, 0.90]	2016
Srikuea 2013	38.9	9.1	11	37.1	6.6	11	6.0%	0.22 [−0.62, 1.06]	2013
Lowe 2006	35.9	4.6	15	33.4	6	15	8.0%	0.45 [−0.27, 1.18]	2006
**Total (95% CI)**			**184**			**182**	**100.0%**	**0.21 [0.00, 0.41]**	

Heterogeneity: $Tau^2 = 0.00$; $Chi^2 = 2.84$, df = 5 (P = 0.73); $I^2 = 0\%$
Test for overall effect: Z = 1.96 (P = 0.05)

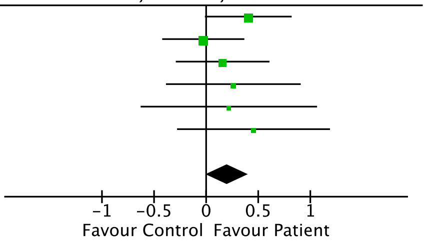

Figure L1. Fat Mass values for all studies after sensitivity analysis.

Study or Subgroup	Patients Mean	Patients SD	Patients Total	Control Mean	Control SD	Control Total	Weight	Std. Mean Difference IV, Random, 95% CI	Year
Cook 2022	7.9	2.5	178	8.8	2.6	169	12.1%	−0.35 [−0.56, −0.14]	2022
Bachassons 2013	5.3	2	11	8.6	1.7	11	6.9%	−1.71 [−2.72, −0.71]	2013
Bardal 2013	6.9	1.7	12	10	2.3	12	7.4%	−1.48 [−2.40, −0.56]	2013
Suarez 2010	3.1	1.77	44	6.6	1.7	25	9.7%	−1.98 [−2.58, −1.39]	2010
Nijis 2010	2.9	1.5	22	2.6	1.1	22	9.8%	0.22 [−0.37, 0.82]	2010
Valkeinenen 2008	7.1	1.2	23	9.1	1.7	11	8.2%	−1.42 [−2.22, −0.62]	2008
Wallman 2004	1.7	0.8	31	2.3	0.9	31	10.3%	−0.70 [−1.21, −0.18]	2004
Georgiades 2003	5.6	3.5	12	7.3	1.3	11	8.0%	−0.61 [−1.45, 0.23]	2003
Norregard 1994	4.4	1.7	15	4.9	1.6	15	8.8%	−0.29 [−1.01, 0.43]	1994
Riley 1990	4.2	1.5	13	5.4	1.2	13	8.2%	−0.86 [−1.66, −0.05]	1990
Sargent 2002	10.6	2.9	33	10.9	2.5	33	10.5%	−0.11 [−0.59, 0.37]	
**Total (95% CI)**			**394**			**353**	**100.0%**	**−0.79 [−1.18, −0.39]**	

Heterogeneity: $Tau^2 = 0.33$; $Chi^2 = 50.06$, df = 10 ($P < 0.00001$); $I^2 = 80\%$
Test for overall effect: Z = 3.90 ($P < 0.0001$)

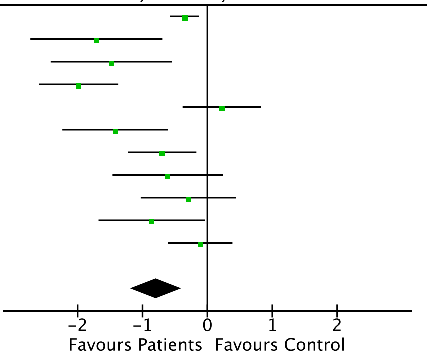

Figure M. Lactate values for all studies included in the analysis.

Study or Subgroup	Patients Mean	Patients SD	Patients Total	Control Mean	Control SD	Control Total	Weight	Std. Mean Difference IV, Random, 95% CI	Year
Cook 2022	7.9	2.5	178	8.8	2.6	169	42.4%	−0.35 [−0.56, −0.14]	2022
Nijis 2010	2.9	1.5	22	2.6	1.1	22	11.7%	0.22 [−0.37, 0.82]	2010
Wallman 2004	1.7	0.8	31	2.3	0.9	31	14.8%	−0.70 [−1.21, −0.18]	2004
Georgiades 2003	5.6	3.5	12	7.3	1.3	11	6.4%	−0.61 [−1.45, 0.23]	2003
Norregard 1994	4.4	1.7	15	4.9	1.6	15	8.4%	−0.29 [−1.01, 0.43]	1994
Sargent 2002	10.6	2.9	33	10.9	2.5	33	16.3%	−0.11 [−0.59, 0.37]	
**Total (95% CI)**			**291**			**281**	**100.0%**	**−0.31 [−0.53, −0.08]**	

Heterogeneity: $Tau^2 = 0.02$; $Chi^2 = 6.57$, df = 5 ($P = 0.25$); $I^2 = 24\%$
Test for overall effect: Z = 2.70 ($P = 0.007$)

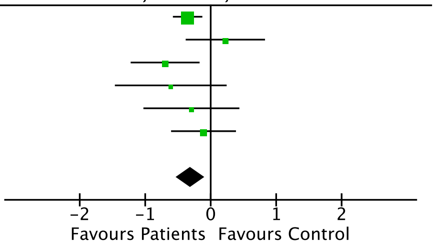

Figure M1. Lactate values for all studies after sensitivity analysis.

Study or Subgroup	Patients Mean	Patients SD	Patients Total	Control Mean	Control SD	Control Total	Weight	Std. Mean Difference IV, Random, 95% CI	Year
**15.3.1 CFS**									
Sargent 2002	10.6	2.9	33	10.9	2.5	33	10.5%	−0.11 [−0.59, 0.37]	
Riley 1990	4.2	1.5	13	5.4	1.2	13	8.2%	−0.86 [−1.66, −0.05]	1990
Georgiades 2003	5.6	3.5	12	7.3	1.3	11	8.0%	−0.61 [−1.45, 0.23]	2003
Wallman 2004	1.7	0.8	31	2.3	0.9	31	10.3%	−0.70 [−1.21, −0.18]	2004
Suarez 2010	3.1	1.77	44	6.6	1.7	25	9.7%	−1.98 [−2.58, −1.39]	2010
Nijis 2010	2.9	1.5	22	2.6	1.1	22	9.8%	0.22 [−0.37, 0.82]	2010
Cook 2022	7.9	2.5	178	8.8	2.6	169	12.1%	−0.35 [−0.56, −0.14]	2022
**Subtotal (95% CI)**			**333**			**304**	**68.6%**	**−0.60 [−1.07, −0.14]**	
Heterogeneity: $Tau^2 = 0.30$; $Chi^2 = 34.93$, df = 6 ($P < 0.00001$); $I^2 = 83\%$									
Test for overall effect: Z = 2.55 ($P = 0.01$)									
**15.3.2 FMS**									
Norregard 1994	4.4	1.7	15	4.9	1.6	15	8.8%	−0.29 [−1.01, 0.43]	1994
Valkeinenen 2008	7.1	1.2	23	9.1	1.7	11	8.2%	−1.42 [−2.22, −0.62]	2008
Bardal 2013	6.9	1.7	12	10	2.3	12	7.4%	−1.48 [−2.40, −0.56]	2013
Bachassons 2013	5.3	2	11	8.6	1.7	11	6.9%	−1.71 [−2.72, −0.71]	2013
**Subtotal (95% CI)**			**61**			**49**	**31.4%**	**−1.18 [−1.85, −0.50]**	
Heterogeneity: $Tau^2 = 0.28$; $Chi^2 = 7.48$, df = 3 ($P = 0.06$); $I^2 = 60\%$									
Test for overall effect: Z = 3.42 ($P = 0.0006$)									
**Total (95% CI)**			**394**			**353**	**100.0%**	**−0.79 [−1.18, −0.39]**	

Heterogeneity: $Tau^2 = 0.33$; $Chi^2 = 50.06$, df = 10 ($P < 0.00001$); $I^2 = 80\%$
Test for overall effect: Z = 3.90 ($P < 0.0001$)
Test for subgroup differences: $Chi^2 = 1.88$, df = 1 ($P = 0.17$), $I^2 = 46.8\%$

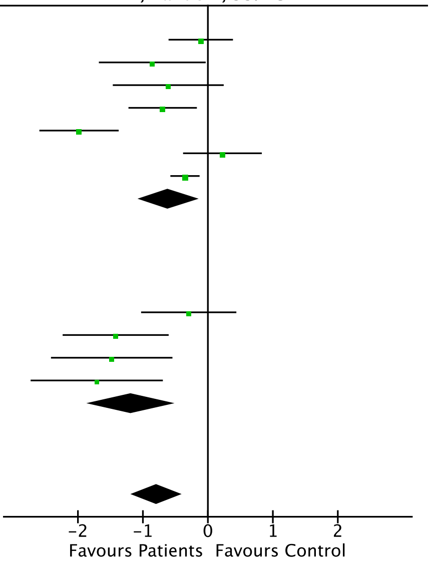

Figure M2. Lactate values in CFS and FMS patients

Study or Subgroup	Patients Mean	Patients SD	Patients Total	Control Mean	Control SD	Control Total	Weight	Std. Mean Difference IV, Random, 95% CI	Year
**1.4.1 No PA matched**									
Cook 2022	23.4	8.6	178	29.9	10.9	169	5.6%	−0.66 [−0.88, −0.45]	2022
Sener 2016	40	10	39	37	8.4	40	4.2%	0.32 [−0.12, 0.77]	2016
Da Cunha 2011	22	3.7417	14	32	7.4833	14	2.1%	−1.64 [−2.52, −0.77]	2011
Nijis 2010	24.1	13.9	22	27.6	24.7	22	3.3%	−0.17 [−0.76, 0.42]	2010
Vermeulen 2010	22.3	5.7	15	31.2	7	15	2.4%	−1.36 [−2.16, −0.55]	2010
Fulcher 2000	30.6	8.2	66	34.1	6.8	30	4.2%	−0.45 [−0.88, −0.01]	2000
**Subtotal (95% CI)**			**334**			**290**	**21.7%**	**−0.57 [−1.03, −0.11]**	
Heterogeneity: $Tau^2 = 0.25$; $Chi^2 = 27.40$, df = 5 (P < 0.0001); $I^2 = 82\%$									
Test for overall effect: Z = 2.44 (P = 0.01)									
**1.4.2 PA matched sedentary/self reported**									
Nelson 2019	27.3	9.2	10	29.9	6.1	10	2.1%	−0.32 [−1.20, 0.56]	2019
Oosterwijick 2017	16.9	4.2	20	19.9	6.9	20	3.1%	−0.51 [−1.15, 0.12]	2017
Vermeulen 2014	20.7	5.4	203	27.5	6.9	18	3.8%	−1.23 [−1.72, −0.73]	2014
Cook 2012 S	781	302	12	907	330	13	2.4%	−0.38 [−1.18, 0.41]	2012
Cook 2012 S	873	290	8	907	330	13	2.1%	−0.10 [−0.98, 0.78]	2012
Cook 2012 M	24.2	5.4	12	30.3	8.1	13	2.3%	−0.85 [−1.68, −0.02]	2012
Cook 2012 M	26.2	9	8	30.3	8.1	13	2.1%	−0.47 [−1.36, 0.43]	2012
Da Cunha 2011	22	3.7	14	32	7.4	14	2.1%	−1.66 [−2.54, −0.78]	2011
Nijis 2010	24.1	13.9	22	27.6	24.7	22	3.3%	−0.17 [−0.76, 0.42]	2010
Patrick – Neary 2008	23.8	10	6	33	3	8	1.4%	−1.26 [−2.45, −0.06]	2008
Gallagher 2005	27.8	7.6	42	31.3	5.8	42	4.2%	−0.51 [−0.95, −0.08]	2005
Jammes 2005	24	9.2	26	37	11.6	11	2.5%	−1.28 [−2.05, −0.51]	2005
Lund 2003	26.6	6	9	36.4	6	9	1.6%	−1.56 [−2.64, −0.47]	2003
Cook 2003	29.8	5.8	19	30.7	4.6	20	3.1%	−0.17 [−0.80, 0.46]	2003
Georgiades 2003	21.2	6.3	12	28.3	6.4	11	2.1%	−1.08 [−1.96, −0.19]	2003
Valim 2002	26	5.2	50	31	5.6	50	4.4%	−0.92 [−1.33, −0.51]	2002
Inbar 2001	19.8	5.3	12	27.3	5.6	15	2.2%	−1.33 [−2.18, −0.48]	2001
Rowbottom 1998	34.7	17.2	33	36.2	10.2	20	3.5%	−0.10 [−0.65, 0.46]	1998
Sisto 1996 M	28	5	10	32	4	17	2.3%	−0.88 [−1.71, −0.06]	1996
Sisto 1996 S	23.9	5.6	11	23.4	2.7	5	1.6%	0.10 [−0.96, 1.15]	1996
Mengshoel 1995	28.2	1.8	37	30.2	2.4	20	3.4%	−0.97 [−1.55, −0.40]	1995
Simms 1994	30	8.1	13	32	7.2	13	2.5%	−0.25 [−1.03, 0.52]	1994
Sargent 2002	35.9	7.7	33	38.6	8.4	33	3.9%	−0.33 [−0.82, 0.15]	
**Subtotal (95% CI)**			**622**			**410**	**61.9%**	**−0.67 [−0.87, −0.48]**	
Heterogeneity: $Tau^2 = 0.09$; $Chi^2 = 39.45$, df = 22 (P = 0.01); $I^2 = 44\%$									
Test for overall effect: Z = 6.76 (P < 0.00001)									
**1.4.4 PA matched questionnaires/accelerometer**									
Bouquet 2019	23.2	8.8	14	25.6	4.5	14	2.6%	−0.33 [−1.08, 0.41]	2019
Bardal 2015	1.45	0.2	16	1.6	0.29	19	2.9%	−0.58 [−1.26, 0.10]	2015
Cook 2006	26	6	29	29.7	8	32	3.8%	−0.51 [−1.02, −0.00]	2006
Cook 2006	23	4	23	30	8	32	3.4%	−1.04 [−1.61, −0.47]	2006
Wallman 2004	16.3	5.6	31	19.9	6.7	31	3.8%	−0.58 [−1.08, −0.07]	2004
**Subtotal (95% CI)**			**113**			**128**	**16.4%**	**−0.63 [−0.89, −0.37]**	
Heterogeneity: $Tau^2 = 0.00$; $Chi^2 = 2.84$, df = 4 (P = 0.59); $I^2 = 0\%$									
Test for overall effect: Z = 4.71 (P < 0.00001)									
**Total (95% CI)**			**1069**			**828**	**100.0%**	**−0.63 [−0.79, −0.48]**	
Heterogeneity: $Tau^2 = 0.10$; $Chi^2 = 71.33$, df = 33 (P = 0.0001); $I^2 = 54\%$									
Test for overall effect: Z = 7.91 (P < 0.00001)									
Test for subgroup differences: $Chi^2 = 0.19$, df = 2 (P = 0.91), $I^2 = 0\%$									

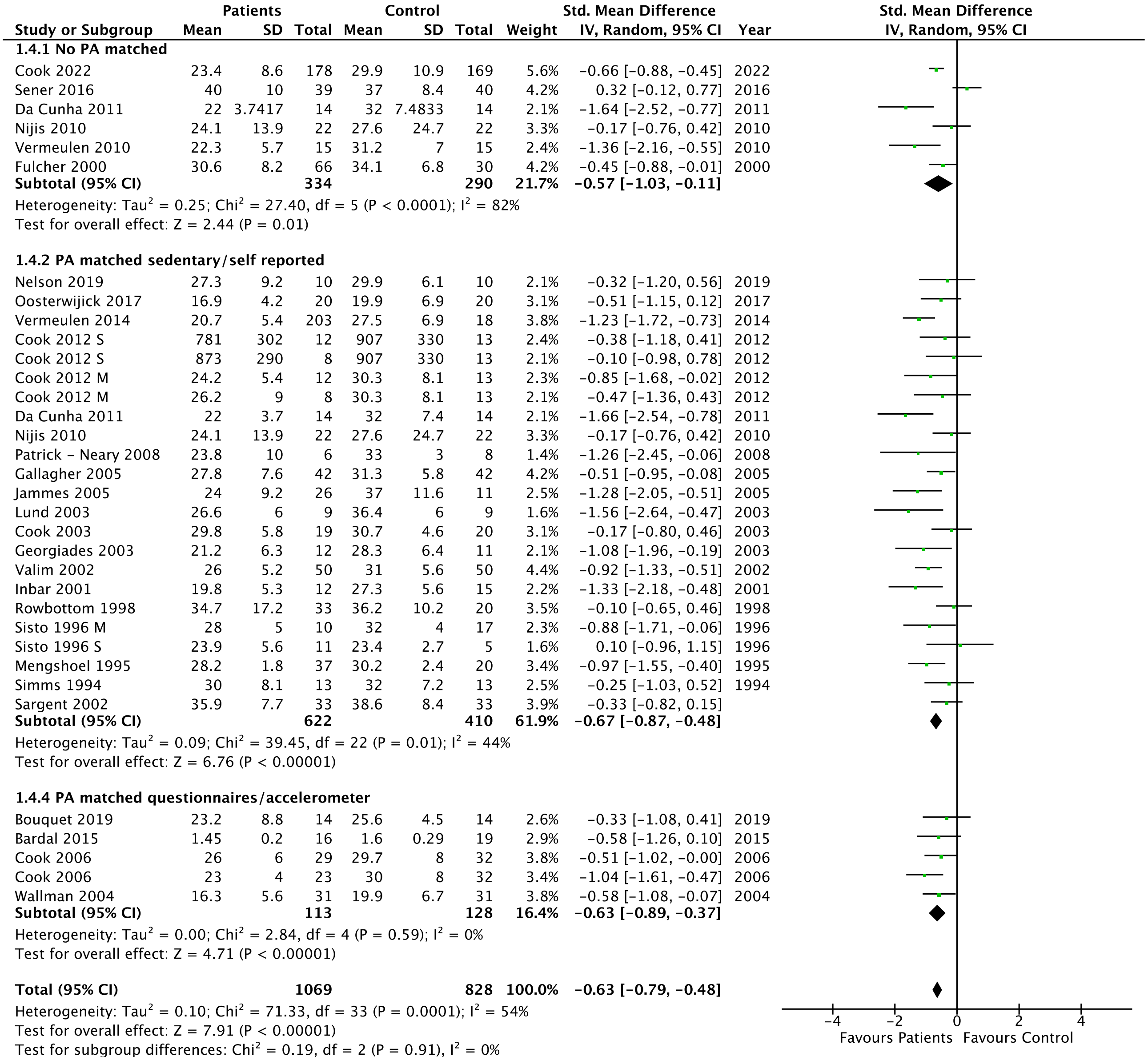

Figure N. Sensitivity analysis for low risk of bias studies based on matched physical activity levels (only VO2 Outcome)

Study or Subgroup	Patients Mean	Patients SD	Patients Total	Control Mean	Control SD	Control Total	Weight	Std. Mean Difference IV, Random, 95% CI	Year
**1.6.1 no pharmacological wash out**									
Cook 2022	23.4	8.6	178	29.9	10.9	169	6.0%	−0.66 [−0.88, −0.45]	2022
Bouquet 2019	23.2	8.8	14	25.6	4.5	14	2.6%	−0.33 [−1.08, 0.41]	2019
Sener 2016	40	10	39	37	8.4	40	4.3%	0.32 [−0.12, 0.77]	2016
Cook 2012 S	873	290	8	907	330	13	2.1%	−0.10 [−0.98, 0.78]	2012
Cook 2012 M	24.2	5.4	12	30.3	8.1	13	2.3%	−0.85 [−1.68, −0.02]	2012
Cook 2012 M	26.2	9	8	30.3	8.1	13	2.1%	−0.47 [−1.36, 0.43]	2012
Cook 2012 S	781	302	12	907	330	13	2.4%	−0.38 [−1.18, 0.41]	2012
Da Cunha 2011	22	3.7	14	32	7.4	14	2.1%	−1.66 [−2.54, −0.78]	2011
Nijis 2010	24.1	13.9	22	27.6	24.7	22	3.4%	−0.17 [−0.76, 0.42]	2010
Patrick − Neary 2008	23.8	10	6	33	3	8	1.3%	−1.26 [−2.45, −0.06]	2008
Jammes 2005	24	9.2	26	37	11.6	11	2.5%	−1.28 [−2.05, −0.51]	2005
Cook 2003	29.8	5.8	19	30.7	4.6	20	3.2%	−0.17 [−0.80, 0.46]	2003
Lund 2003	26.6	6	9	36.4	6	9	1.5%	−1.56 [−2.64, −0.47]	2003
Georgiades 2003	21.2	6.3	12	28.3	6.4	11	2.1%	−1.08 [−1.96, −0.19]	2003
Valim 2002	26	5.2	50	31	5.6	50	4.6%	−0.92 [−1.33, −0.51]	2002
Bazelman 2001	2	0.7	20	2.2	0.6	20	3.2%	−0.30 [−0.92, 0.32]	2001
Fulcher 2000	30.6	8.2	66	34.1	6.8	30	4.4%	−0.45 [−0.88, −0.01]	2000
Rowbottom 1998	34.7	17.2	33	36.2	10.2	20	3.6%	−0.10 [−0.65, 0.46]	1998
Mengshoel 1995	28.2	1.8	37	30.2	2.4	20	3.5%	−0.97 [−1.55, −0.40]	1995
Simms 1994	30	8.1	13	32	7.2	13	2.5%	−0.25 [−1.03, 0.52]	1994
**Subtotal (95% CI)**			**598**			**523**	**59.7%**	**−0.56 [−0.78, −0.35]**	
Heterogeneity: $Tau^2 = 0.12$; $Chi^2 = 45.52$, df = 19 (P = 0.0006); $I^2 = 58\%$									
Test for overall effect: Z = 5.14 (P < 0.00001)									
**1.6.2 yes pharmacological wash out**									
Nelson 2019	27.3	9.2	10	29.9	6.1	10	2.1%	−0.32 [−1.20, 0.56]	2019
Oosterwijick 2017	16.9	4.2	20	19.9	6.9	20	3.2%	−0.51 [−1.15, 0.12]	2017
Bardal 2015	1.45	0.2	16	1.6	0.29	19	2.9%	−0.58 [−1.26, 0.10]	2015
Vermeulen 2014	20.7	5.4	203	27.5	6.9	18	4.0%	−1.23 [−1.72, −0.73]	2014
Vermeulen 2010	22.3	5.7	15	31.2	7	15	2.4%	−1.36 [−2.16, −0.55]	2010
Cook 2006	23	4	23	30	8	32	3.5%	−1.04 [−1.61, −0.47]	2006
Cook 2006	26	6	29	29.7	8	32	3.9%	−0.51 [−1.02, −0.00]	2006
Gallagher 2005	27.8	7.6	42	31.3	5.8	42	4.4%	−0.51 [−0.95, −0.08]	2005
Wallman 2004	16.3	5.6	31	19.9	6.7	31	3.9%	−0.58 [−1.08, −0.07]	2004
Inbar 2001	19.8	5.3	12	27.3	5.6	15	2.2%	−1.33 [−2.18, −0.48]	2001
Sisto 1996 S	23.9	5.6	11	23.4	2.7	5	1.6%	0.10 [−0.96, 1.15]	1996
Sisto 1996 M	28	5	10	32	4	17	2.3%	−0.88 [−1.71, −0.06]	1996
Sargent 2002	35.9	7.7	33	38.6	8.4	33	4.0%	−0.33 [−0.82, 0.15]	
**Subtotal (95% CI)**			**455**			**289**	**40.3%**	**−0.70 [−0.91, −0.49]**	
Heterogeneity: $Tau^2 = 0.04$; $Chi^2 = 17.51$, df = 12 (P = 0.13); $I^2 = 31\%$									
Test for overall effect: Z = 6.57 (P < 0.00001)									
**Total (95% CI)**			**1053**			**812**	**100.0%**	**−0.61 [−0.77, −0.46]**	
Heterogeneity: $Tau^2 = 0.09$; $Chi^2 = 64.78$, df = 32 (P = 0.0005); $I^2 = 51\%$									
Test for overall effect: Z = 7.86 (P < 0.00001)									
Test for subgroup differences: $Chi^2 = 0.81$, df = 1 (P = 0.37), $I^2 = 0\%$									

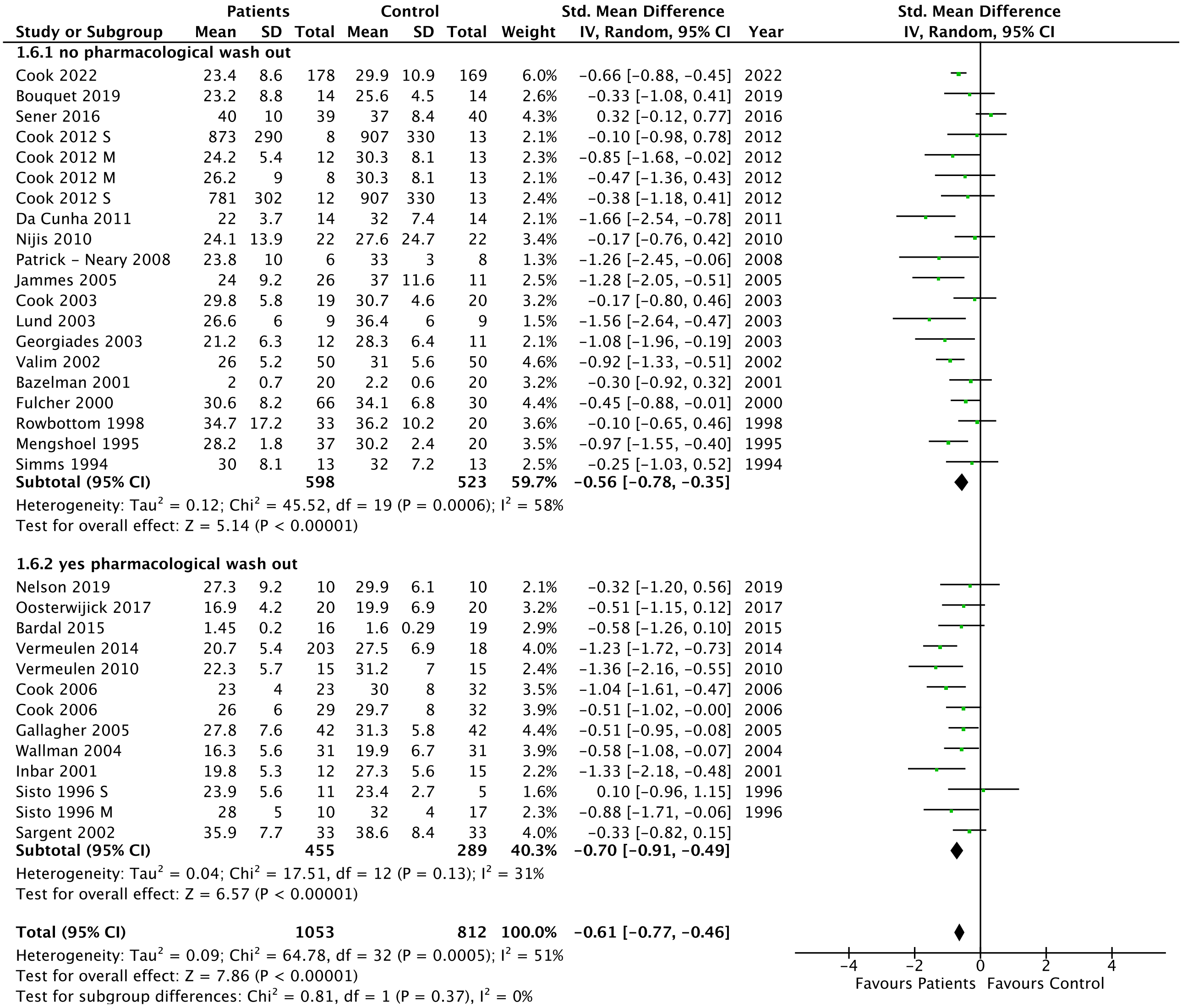

Figure 0. Sensitivity analysis for low risk of bias studies based on presence of pharmacological wash-out prior to the test levels (only VO2 outcome)

Study or Subgroup	Patient Mean	Patient SD	Patient Total	Control Mean	Control SD	Control Total	Weight	Std. Mean Difference IV, Random, 95% CI	Year
**9.4.1 PA matched questionners/accelerometer**									
Berardi 2021	38	12.2	47	40	12.2	47	5.8%	−0.16 [−0.57, 0.24]	2021
Berardi 2021	27.7	7.9	47	29.2	8.4	47	5.8%	−0.18 [−0.59, 0.22]	2021
Icksman 2013	50.9	13.3	30	58.9	20	13	3.3%	−0.50 [−1.16, 0.16]	2013
Srikuea 2013	114.5	20.1	11	131.5	30.5	11	2.2%	−0.63 [−1.49, 0.23]	2013
Goes 2012	31.14	18.1	16	47.3	20.1	16	2.8%	−0.82 [−1.55, −0.10]	2012
**Subtotal (95% CI)**			**151**			**134**	**19.9%**	**−0.32 [−0.56, −0.08]**	
Heterogeneity: $Tau^2 = 0.00$; $Chi^2 = 3.68$, df = 4 (P = 0.45); $I^2 = 0\%$									
Test for overall effect: Z = 2.64 (P = 0.008)									
**9.4.2 PA matched sedentary/self reported**									
Wahlen 2022	154	101.5	40	234	41.5	24	4.3%	−0.94 [−1.47, −0.40]	2022
Wahlen 2022	299	128	40	399.5	90.3	24	4.4%	−0.86 [−1.39, −0.33]	2022
Wahlen 2022	11.8	7.9	40	18.4	7.8	24	4.4%	−0.83 [−1.36, −0.30]	2022
Gerdle 2016 HG	12.8	4.6	29	17	4.5	28	4.2%	−0.91 [−1.46, −0.36]	2016
Gerdle 2016 Lext	340.8	98.1	29	370.1	85.8	28	4.4%	−0.31 [−0.84, 0.21]	2016
Icksman 2014	28.9	7.74	30	33.7	10.1	30	4.5%	−0.53 [−1.04, −0.01]	2014
Icksman 2014	32.8	7	30	33.7	10.1	30	4.6%	−0.10 [−0.61, 0.40]	2014
Klaver−Kol 2012	92.9	20	13	114.2	17.9	14	2.4%	−1.09 [−1.91, −0.27]	2012
Giske 2008	407	118	19	574	99	19	2.8%	−1.50 [−2.23, −0.77]	2008
Sahin 2004	20.1	5.9	41	23	4.8	40	5.3%	−0.53 [−0.98, −0.09]	2004
Fulcher 2000	310	115.2	66	441	119.8	30	5.1%	−1.11 [−1.57, −0.65]	2000
Sacco 1999	159.5	23	10	180.5	15.5	10	1.9%	−1.03 [−1.97, −0.08]	1999
Simms 1994	20.9	5.7	13	21.4	3.3	13	2.6%	−0.10 [−0.87, 0.67]	1994
Simms 1994	50.1	22	13	55.8	17.5	13	2.6%	−0.28 [−1.05, 0.50]	1994
**Subtotal (95% CI)**			**413**			**327**	**53.4%**	**−0.71 [−0.91, −0.50]**	
Heterogeneity: $Tau^2 = 0.07$; $Chi^2 = 22.87$, df = 13 (P = 0.04); $I^2 = 43\%$									
Test for overall effect: Z = 6.69 (P < 0.00001)									
**9.4.3 no PA matched**									
Jakel 2021	264	108	105	333	82	66	7.2%	−0.70 [−1.01, −0.38]	2021
Jakel 2021	31.4	8.8	105	37.1	8.8	66	7.2%	−0.64 [−0.96, −0.33]	2021
Sener 2016	19.6	5.6	39	24.5	4.5	40	5.0%	−0.96 [−1.42, −0.49]	2016
Umeda 2015	22.5	5.3	14	28.6	6.8	14	2.5%	−0.97 [−1.76, −0.18]	2015
Panton 2006	39	11	29	40	12	12	3.2%	−0.09 [−0.76, 0.59]	2006
Semionow 2004	305.8	150.4	8	412	103.6	8	1.6%	−0.78 [−1.81, 0.25]	2004
**Subtotal (95% CI)**			**300**			**206**	**26.7%**	**−0.69 [−0.88, −0.51]**	
Heterogeneity: $Tau^2 = 0.00$; $Chi^2 = 4.93$, df = 5 (P = 0.42); $I^2 = 0\%$									
Test for overall effect: Z = 7.34 (P < 0.00001)									
**Total (95% CI)**			**864**			**667**	**100.0%**	**−0.63 [−0.78, −0.49]**	
Heterogeneity: $Tau^2 = 0.05$; $Chi^2 = 39.23$, df = 24 (P = 0.03); $I^2 = 39\%$									
Test for overall effect: Z = 8.79 (P < 0.00001)									
Test for subgroup differences: $Chi^2 = 7.29$, df = 2 (P = 0.03), $I^2 = 72.5\%$									

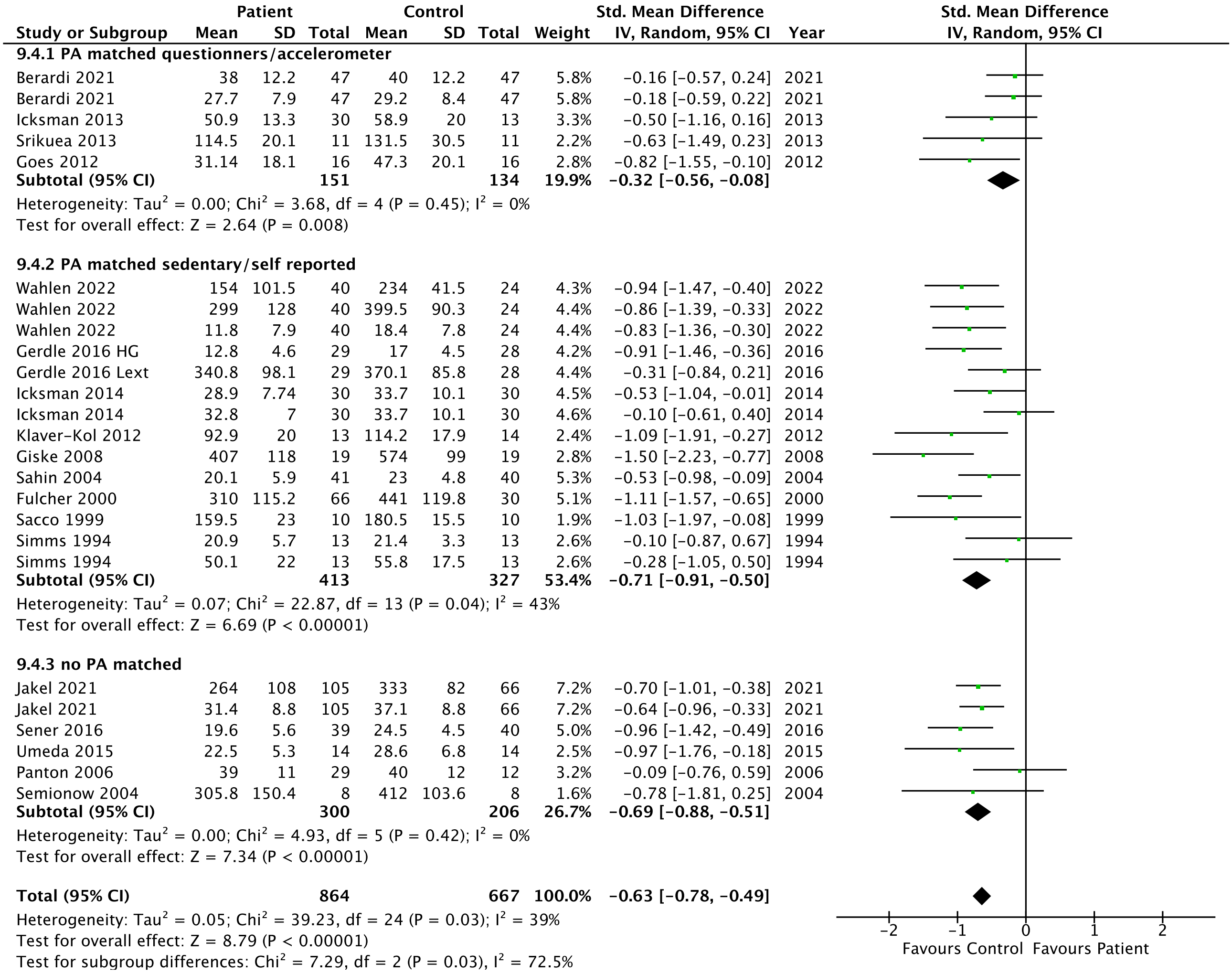

Figure P. Sub-group analysis for low risk of bias studies based on matched physical activity levels (only MVC Outcome)

Study or Subgroup	Patient Mean	Patient SD	Patient Total	Control Mean	Control SD	Control Total	Weight	Std. Mean Difference IV, Random, 95% CI	Year
**9.5.1 No pharmacological wash out**									
Jakel 2021	31.4	8.8	105	37.1	8.8	66	7.2%	−0.64 [−0.96, −0.33]	2021
Jakel 2021	264	108	105	333	82	66	7.2%	−0.70 [−1.01, −0.38]	2021
Berardi 2021	38	12.2	47	40	12.2	47	5.8%	−0.16 [−0.57, 0.24]	2021
Berardi 2021	27.7	7.9	47	29.2	8.4	47	5.8%	−0.18 [−0.59, 0.22]	2021
Sener 2016	19.6	5.6	39	24.5	4.5	40	5.0%	−0.96 [−1.42, −0.49]	2016
Goes 2012	31.14	18.1	16	47.3	20.1	16	2.8%	−0.82 [−1.55, −0.10]	2012
Giske 2008	407	118	19	574	99	19	2.8%	−1.50 [−2.23, −0.77]	2008
Panton 2006	39	11	29	40	12	12	3.2%	−0.09 [−0.76, 0.59]	2006
Fulcher 2000	310	115.2	66	441	119.8	30	5.1%	−1.11 [−1.57, −0.65]	2000
Sacco 1999	159.5	23	10	180.5	15.5	10	1.9%	−1.03 [−1.97, −0.08]	1999
Simms 1994	50.1	22	13	55.8	17.5	13	2.6%	−0.28 [−1.05, 0.50]	1994
Simms 1994	20.9	5.7	13	21.4	3.3	13	2.6%	−0.10 [−0.87, 0.67]	1994
**Subtotal (95% CI)**			**509**			**379**	**52.0%**	**−0.62 [−0.85, −0.38]**	
Heterogeneity: $Tau^2 = 0.09$; $Chi^2 = 27.51$, df = 11 (P = 0.004); $I^2 = 60\%$									
Test for overall effect: Z = 5.12 (P < 0.00001)									
**9.5.2 pharmacological wash out**									
Wahlen 2022	11.8	7.9	40	18.4	7.8	24	4.4%	−0.83 [−1.36, −0.30]	2022
Wahlen 2022	299	128	40	399.5	90.3	24	4.4%	−0.86 [−1.39, −0.33]	2022
Wahlen 2022	154	101.5	40	234	41.5	24	4.3%	−0.94 [−1.47, −0.40]	2022
Gerdle 2016 HG	12.8	4.6	29	17	4.5	28	4.2%	−0.91 [−1.46, −0.36]	2016
Gerdle 2016 Lext	340.8	98.1	29	370.1	85.8	28	4.4%	−0.31 [−0.84, 0.21]	2016
Umeda 2015	22.5	5.3	14	28.6	6.8	14	2.5%	−0.97 [−1.76, −0.18]	2015
Icksman 2014	28.9	7.74	30	33.7	10.1	30	4.5%	−0.53 [−1.04, −0.01]	2014
Icksman 2014	32.8	7	30	33.7	10.1	30	4.6%	−0.10 [−0.61, 0.40]	2014
Icksman 2013	50.9	13.3	30	58.9	20	13	3.3%	−0.50 [−1.16, 0.16]	2013
Srikuea 2013	114.5	20.1	11	131.5	30.5	11	2.2%	−0.63 [−1.49, 0.23]	2013
Klaver–Kol 2012	92.9	20	13	114.2	17.9	14	2.4%	−1.09 [−1.91, −0.27]	2012
Sahin 2004	20.1	5.9	41	23	4.8	40	5.3%	−0.53 [−0.98, −0.09]	2004
Semionow 2004	305.8	150.4	8	412	103.6	8	1.6%	−0.78 [−1.81, 0.25]	2004
**Subtotal (95% CI)**			**355**			**288**	**48.0%**	**−0.64 [−0.81, −0.48]**	
Heterogeneity: $Tau^2 = 0.00$; $Chi^2 = 11.60$, df = 12 (P = 0.48); $I^2 = 0\%$									
Test for overall effect: Z = 7.80 (P < 0.00001)									
**Total (95% CI)**			**864**			**667**	**100.0%**	**−0.63 [−0.78, −0.49]**	
Heterogeneity: $Tau^2 = 0.05$; $Chi^2 = 39.23$, df = 24 (P = 0.03); $I^2 = 39\%$									
Test for overall effect: Z = 8.79 (P < 0.00001)									
Test for subgroup differences: $Chi^2 = 0.04$, df = 1 (P = 0.85), $I^2 = 0\%$									

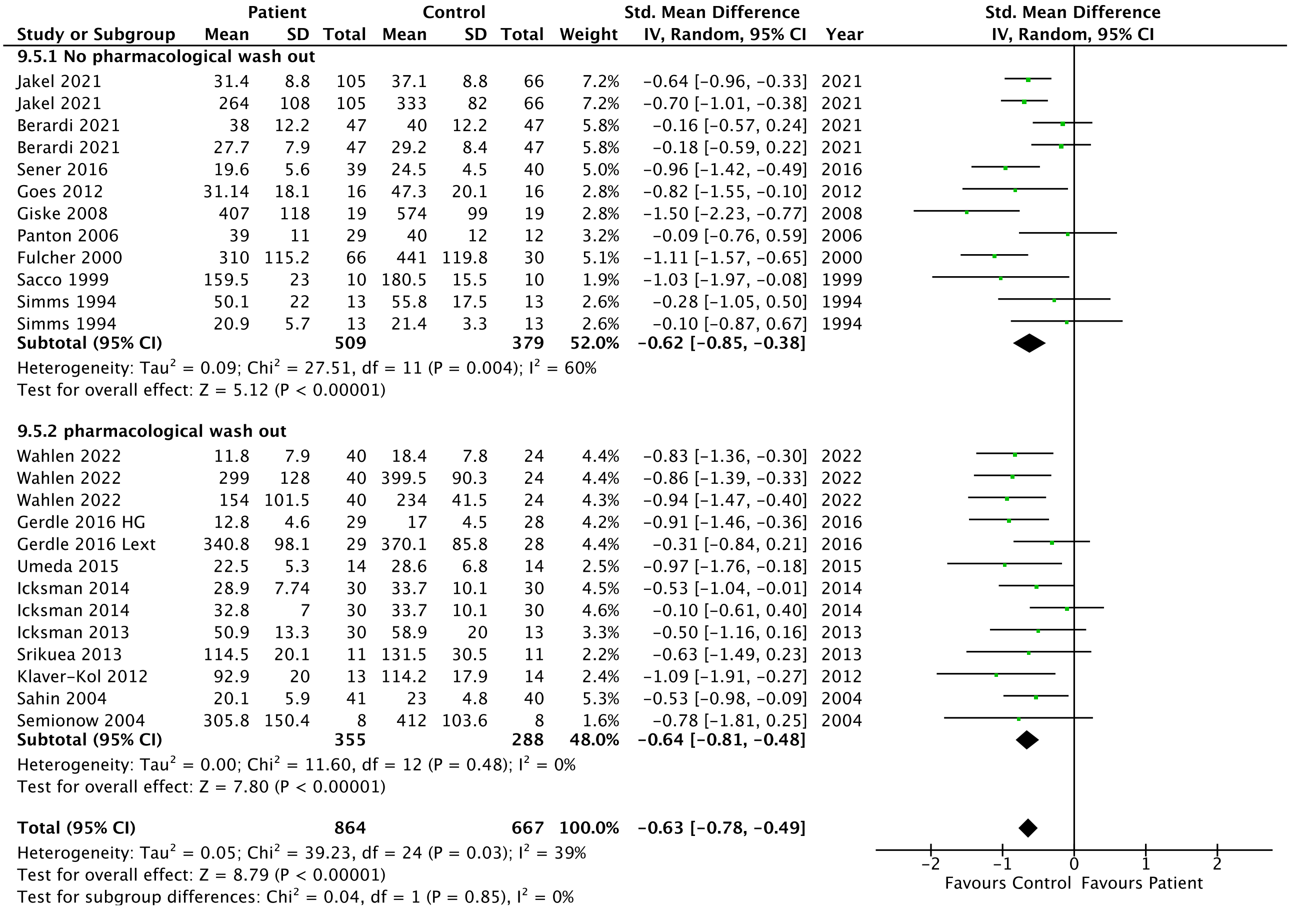

Figure Q. Sub-group analysis for low risk of bias studies based on presence of pharmacological wash-out prior to the test levels (only MVC outcome)
