# Supplementary material for: Fibromyalgia and Chronic Fatigue Syndromes: A systematic review and meta-analysis of cardiorespiratory fitness and neuromuscular function compared with healthy individuals
Source: PLoS One. 2022 Oct 20;17(10):e0276009. doi: 10.1371/journal.pone.0276009 (PMC9584387; doi:10.1371/journal.pone.0276009)
Supplement: S4 File — (PDF) [file pone.0276009.s004.pdf]

Fibromyalgia and Chronic Fatigue Syndrome: a systematic review and meta-analysis of cardiorespiratory fitness and neuromuscular function.

Research term table

(FT= Full Text; MH= Mesh terms)

Search *Term*	Field
Fibromyalgia	MH
Fatigue Syndrome, Chronic	MH
Fibromyalgia	FT
Chronic Fatigue syndrome	FT
Myalgic Encephalomyelitis	FT
All population combined with OR	
Exercise test	MH
Physical fitness	FT
Functional capacity	FT
Exercise performance *	FT
Aerobic power	FT
Peak power *	FT
Aerobic fitness	FT
Heart Rate *	FT
Aerobic assessment	FT
Oxygen uptake N2 maximum OR peak	FT
Threshold N2 Anaerobic OR Ventilatory OR lactate	FT
Cardiorespiratory Fitness	FT
Aerobic Capacity	FT
Lactate	FT
Rate of perceived exertion OR RPE	FT
All Aerobic Assessment outcomes combined with OR	
Muscle strength	MH
Muscle strength dynamometer	MH
Muscle strength	FT
Muscle strength dynamometer	FT
Muscle Strength Assessment	FT
Maximal Voluntary Contraction	FT
Voluntary Activation	FT
Fatigability	FT
Endurance *	FT
Maximal strength	FT
Handgrip assessment	FT
Handgrip test	FT
Rate of Torque	FT
Peak Torque	FT
All Strength outcomes combined with OR	
Body composition	MH
Body Mass Index	FT
Muscle volume	FT
Muscle cross-sectional area	FT
Mass N2 lean body OR free fat OR fat OR muscle	FT
All body composition outcomes combined with OR	

ALL Outcomes combine with AND Fibromyalgia OR Chronic Fatigue	

FT= Full Text; MH= Mesh terms

Research strategy PUBMED.

Search ((((((((((((((((((((Exercise test[MeSH Terms]) OR Exercise test) OR Physical fitness) OR Functional capacity) OR Exercise performance *) OR Aerobic power) OR Peak power *) OR Aerobic fitness) OR Heart Rate *) OR Aerobic assessment) OR ((Oxygen uptake N2 maximum OR peak))) OR ((Threshold N2 Anaerobic OR Ventilatory OR lactate))) OR Cardiorespiratory Fitness) OR Aerobic Capacity) OR Lactate) OR (Rate of perceived exertion OR RPE))))) OR (((((Body composition[MeSH Terms]) OR Body composition) OR Muscle volume) OR Muscle cross-sectional area) OR ((Mass N2 lean body OR free fat OR fat OR muscle)))) OR (((((((((((((Muscle strength[MeSH Terms]) OR Muscle strength) OR Muscle strength dynamometer) OR Muscle strength dynamometer[MeSH Terms]) OR Muscle Strength Assessment) OR Maximal Voluntary Contraction) OR Voluntary Activation) OR Fatigability) OR Endurance *) OR Maximal strength) OR Handgrip assessment) OR Handgrip test) OR Rate of Torque) OR Peak Torque)) AND ((((((fibromyalgia[MeSH Terms]) OR chronic fatigue syndrome) OR chronic fatigue syndrome[MeSH Terms]) OR fibromyalgia) OR Myalgic Encephalomyelitis) OR Myalgic Encephalomyelitis[MeSH Terms]) Sort by: Best Match

Research strategy Cochrane (CENTRAL)

ID Search Hits

#1 MeSH descriptor: [Fibromyalgia] explode all trees

#2 Fibromyalgia

#3 MeSH descriptor: [Fatigue Syndrome, Chronic] 4 tree(s) exploded

#4 Chronic fatigue syndrome

#5 Myalgic Encephalomyelitis

#6 #1 OR #2 OR #3 OR #4 OR #5

#7 MeSH descriptor: [Exercise Test] explode all trees

#8 exercise test

#9 Physical fitness

#10 Functional capacity

#11 Exercise performance

#12 Aerobic power

#13 Peak power

#14 Aerobic fitness

#15 Heart Rate

#16 Aerobic assessment

#17 Oxygen uptake N2 maximum OR peak

#18 Threshold N2 Anaerobic OR Ventilatory OR lactate

#19 Cardiorespiratory Fitness

#20 Aerobic Capacity

#21 Lactate

#22 Rate of perceived exertion OR RPE

#23 #7 OR #8 OR #9 OR #10 OR #11 OR #12 OR #13 OR #14 OR #15 OR #16 OR #17 OR #18 OR #19 OR #20 OR #20 OR #21 OR #22

#24 Muscle strength

#25 MeSH descriptor: [Muscle Strength] explode all trees

#26 MeSH descriptor: [Muscle Strength Dynamometer] explode all trees

#27 Muscle strength dynamometer

#28 Muscle Strength Assessment

#29 Maximal Voluntary Contraction

#30 Voluntary Activation

#31 Fatigability

#32 Endurance

#33 Maximal strength

#34 Handgrip assessment

#35 Handgrip test

#36 Rate of Torque

#37 Peak Torque

#38 #24 OR #25 OR #26 OR #27 OR #28 OR #29 OR #30 OR #31 OR #32 OR #33 OR #34 OR #35 OR #36 OR #37 36590

#39 Body composition

#40 Body Mass Index

#41 MeSH descriptor: [Body Composition] explode all trees

#42 Muscle volume

#43 Muscle cross-sectional area

#44 Mass N2 lean body OR free fat OR fat OR muscle

#45 #39 OR #40 OR #41 OR #42 OR #43 OR #44

#46 #23 OR #38 OR #45

#47 #6 AND #46

Research strategy PEDRO

Research strategy for PEDRO was performed using only the following words: Fibromyalgia and Chronic fatigue Syndrome separately. The results thereafter were combined in Endnote and duplicates if present removed.

Research Strategy for EBSCOhost: Medline, AMED, CINAHL:

The research strategy was performed trough EBSCOhost database for Medline, AMED and CINAHL databases as reported in the following page (below).

MY
EBSCOhost

Monday, June 06, 2022 4:12:40 PM

#	Query	Limiters/Expanders	Last Run Via	Results
S120	S118 AND S119	Search modes - Boolean/Phrase	Interface - EBSCOhost Research Databases<br>Search Screen - Advanced Search<br>Database - AMED - The Allied and Complementary Medicine Database;CINAHL;MEDLINE	Display
S119	S114 OR S115 OR S116	Search modes - Boolean/Phrase	Interface - EBSCOhost Research Databases<br>Search Screen - Advanced Search<br>Database - AMED - The Allied and Complementary Medicine Database;CINAHL;MEDLINE	Display
S118	S113 OR S117	Search modes - Boolean/Phrase	Interface - EBSCOhost Research Databases<br>Search Screen - Advanced Search<br>Database - AMED - The Allied and Complementary Medicine Database;CINAHL;MEDLINE	Display
S117	myalgic encephalomyelitis	Search modes - Boolean/Phrase	Interface - EBSCOhost Research Databases<br>Search Screen - Advanced Search<br>Database - AMED - The Allied and Complementary Medicine Database;CINAHL;MEDLINE	Display
S116	S63 OR S64 OR S65 OR S66 OR S67 OR S68 OR S69 OR S70 OR S71 OR S72 OR S73 OR S74 OR S75 OR S76 OR S77 OR S78 OR S79 OR S80 OR S81 OR S82 OR S83 OR S84 OR S85 OR S86 OR S87 OR S88 OR S89	Search modes - Boolean/Phrase	Interface - EBSCOhost Research Databases<br>Search Screen - Advanced Search<br>Database - AMED - The Allied and Complementary Medicine Database;CINAHL;MEDLINE	Display
S115	S103 OR S104 OR S105	Search modes -	Interface - EBSCOhost	Display

	OR S106 OR S107 OR S108 OR S109 OR S110 OR S111	Boolean/Phrase	Research Databases<br>Search Screen - Advanced Search<br>Database - MEDLINE	
S114	S90 OR S91 OR S92 OR S93 OR S94 OR S95 OR S96 OR S97 OR S98 OR S99 OR S100 OR S101 OR S102	Search modes - Boolean/Phrase	Interface - EBSCOhost Research Databases<br>Search Screen - Advanced Search<br>Database - MEDLINE	Display
S113	S61 OR S62 OR S112	Search modes - Boolean/Phrase	Interface - EBSCOhost Research Databases<br>Search Screen - Advanced Search<br>Database - MEDLINE	Display
S112	TX chronic fatigue syndrome	Search modes - Boolean/Phrase	Interface - EBSCOhost Research Databases<br>Search Screen - Advanced Search<br>Database - MEDLINE	Display
S111	TX sarcopenia	Search modes - Boolean/Phrase	Interface - EBSCOhost Research Databases<br>Search Screen - Advanced Search<br>Database - MEDLINE	Display
S110	TX muscle cross-sectional area	Search modes - Boolean/Phrase	Interface - EBSCOhost Research Databases<br>Search Screen - Advanced Search<br>Database - MEDLINE	Display
S109	TX Muscle volume	Search modes - Boolean/Phrase	Interface - EBSCOhost Research Databases<br>Search Screen - Advanced Search<br>Database - MEDLINE	Display
S108	TX Muscle mass	Search modes - Boolean/Phrase	Interface - EBSCOhost Research Databases<br>Search Screen - Advanced Search<br>Database - MEDLINE	Display
S107	TX fat mass	Search modes - Boolean/Phrase	Interface - EBSCOhost Research Databases<br>Search Screen - Advanced	Display

			Search<br>Database - MEDLINE	
S106	TX Free fat mass	Search modes - Boolean/Phrase	Interface - EBSCOhost Research Databases<br>Search Screen - Advanced Search<br>Database - MEDLINE	Display
S105	TX lean body mass	Search modes - Boolean/Phrase	Interface - EBSCOhost Research Databases<br>Search Screen - Advanced Search<br>Database - MEDLINE	Display
S104	TX body composition	Search modes - Boolean/Phrase	Interface - EBSCOhost Research Databases<br>Search Screen - Advanced Search<br>Database - MEDLINE	Display
S103	MH body composition	Search modes - Boolean/Phrase	Interface - EBSCOhost Research Databases<br>Search Screen - Advanced Search<br>Database - MEDLINE	Display
S102	TX handgrip	Search modes - Boolean/Phrase	Interface - EBSCOhost Research Databases<br>Search Screen - Advanced Search<br>Database - MEDLINE	Display
S101	TX Handgrip test	Search modes - Boolean/Phrase	Interface - EBSCOhost Research Databases<br>Search Screen - Advanced Search<br>Database - MEDLINE	Display
S100	TX Handgrip assessment	Search modes - Boolean/Phrase	Interface - EBSCOhost Research Databases<br>Search Screen - Advanced Search<br>Database - MEDLINE	Display
S99	TX Maximal strength	Search modes - Boolean/Phrase	Interface - EBSCOhost Research Databases<br>Search Screen - Advanced Search<br>Database - MEDLINE	Display

S98	TX Knee contraction	Search modes - Boolean/Phrase	Interface - EBSCOhost Research Databases<br>Search Screen - Advanced Search<br>Database - MEDLINE	Display
S97	TX Knee maximal contraction	Search modes - Boolean/Phrase	Interface - EBSCOhost Research Databases<br>Search Screen - Advanced Search<br>Database - MEDLINE	Display
S96	TX Maximal isometric contraction	Search modes - Boolean/Phrase	Interface - EBSCOhost Research Databases<br>Search Screen - Advanced Search<br>Database - MEDLINE	Display
S95	TX Isometric contraction	Search modes - Boolean/Phrase	Interface - EBSCOhost Research Databases<br>Search Screen - Advanced Search<br>Database - MEDLINE	Display
S94	TX Maximum voluntary contraction	Search modes - Boolean/Phrase	Interface - EBSCOhost Research Databases<br>Search Screen - Advanced Search<br>Database - MEDLINE	Display
S93	MH Muscle strength	Search modes - Boolean/Phrase	Interface - EBSCOhost Research Databases<br>Search Screen - Advanced Search<br>Database - MEDLINE	Display
S92	MH Muscle strength dynamometer	Search modes - Boolean/Phrase	Interface - EBSCOhost Research Databases<br>Search Screen - Advanced Search<br>Database - MEDLINE	Display
S91	Muscle strength dynamometer	Search modes - Boolean/Phrase	Interface - EBSCOhost Research Databases<br>Search Screen - Advanced Search<br>Database - MEDLINE	Display
S90	Muscle strength	Search modes - Boolean/Phrase	Interface - EBSCOhost Research Databases<br>Search Screen - Advanced	Display

			Search<br>Database - MEDLINE	
S89	TX fitness test	Search modes - Boolean/Phrase	Interface - EBSCOhost Research Databases<br>Search Screen - Advanced Search<br>Database - MEDLINE	Display
S88	TX lactate	Search modes - Boolean/Phrase	Interface - EBSCOhost Research Databases<br>Search Screen - Advanced Search<br>Database - MEDLINE	Display
S87	TX lactate threshold	Search modes - Boolean/Phrase	Interface - EBSCOhost Research Databases<br>Search Screen - Advanced Search<br>Database - MEDLINE	Display
S86	TX Maximal test	Search modes - Boolean/Phrase	Interface - EBSCOhost Research Databases<br>Search Screen - Advanced Search<br>Database - MEDLINE	Display
S85	TX Submaximal test	Search modes - Boolean/Phrase	Interface - EBSCOhost Research Databases<br>Search Screen - Advanced Search<br>Database - MEDLINE	Display
S84	TX Aerobic Capacity	Search modes - Boolean/Phrase	Interface - EBSCOhost Research Databases<br>Search Screen - Advanced Search<br>Database - MEDLINE	Display
S83	TX Cardiorespiratory Fitness	Search modes - Boolean/Phrase	Interface - EBSCOhost Research Databases<br>Search Screen - Advanced Search<br>Database - MEDLINE	Display
S82	TX Ventilatory Threshold	Search modes - Boolean/Phrase	Interface - EBSCOhost Research Databases<br>Search Screen - Advanced Search<br>Database - MEDLINE	Display

S81	TX maximum oxygen uptake	Search modes - Boolean/Phrase	Interface - EBSCOhost Research Databases<br>Search Screen - Advanced Search<br>Database - MEDLINE	Display
S80	TX anaerobic threshold	Search modes - Boolean/Phrase	Interface - EBSCOhost Research Databases<br>Search Screen - Advanced Search<br>Database - MEDLINE	Display
S79	TX maximum oxygen uptake	Search modes - Boolean/Phrase	Interface - EBSCOhost Research Databases<br>Search Screen - Advanced Search<br>Database - MEDLINE	Display
S78	TX Peak oxygen uptake	Search modes - Boolean/Phrase	Interface - EBSCOhost Research Databases<br>Search Screen - Advanced Search<br>Database - MEDLINE	Display
S77	TX Aerobic assessment	Search modes - Boolean/Phrase	Interface - EBSCOhost Research Databases<br>Search Screen - Advanced Search<br>Database - MEDLINE	Display
S76	TX Aerobic Test	Search modes - Boolean/Phrase	Interface - EBSCOhost Research Databases<br>Search Screen - Advanced Search<br>Database - MEDLINE	Display
S75	TX Incremental test	Search modes - Boolean/Phrase	Interface - EBSCOhost Research Databases<br>Search Screen - Advanced Search<br>Database - MEDLINE	Display
S74	TX maximum heart rate	Search modes - Boolean/Phrase	Interface - EBSCOhost Research Databases<br>Search Screen - Advanced Search<br>Database - MEDLINE	Display
S73	TX Heart Rate	Search modes - Boolean/Phrase	Interface - EBSCOhost Research Databases<br>Search Screen - Advanced	Display

			Search<br>Database - MEDLINE	
S72	TX Aerobic fitness	Search modes - Boolean/Phrase	Interface - EBSCOhost Research Databases<br>Search Screen - Advanced Search<br>Database - MEDLINE	Display
S71	TX peak power	Search modes - Boolean/Phrase	Interface - EBSCOhost Research Databases<br>Search Screen - Advanced Search<br>Database - MEDLINE	Display
S70	TX Aerobic power	Search modes - Boolean/Phrase	Interface - EBSCOhost Research Databases<br>Search Screen - Advanced Search<br>Database - MEDLINE	Display
S69	TX Exercise performance	Search modes - Boolean/Phrase	Interface - EBSCOhost Research Databases<br>Search Screen - Advanced Search<br>Database - MEDLINE	Display
S68	TX functional capacity	Search modes - Boolean/Phrase	Interface - EBSCOhost Research Databases<br>Search Screen - Advanced Search<br>Database - MEDLINE	Display
S67	TX Cardiorespiratory	Search modes - Boolean/Phrase	Interface - EBSCOhost Research Databases<br>Search Screen - Advanced Search<br>Database - MEDLINE	Display
S66	TX physical fitness	Search modes - Boolean/Phrase	Interface - EBSCOhost Research Databases<br>Search Screen - Advanced Search<br>Database - MEDLINE	Display
S65	TX Cardiorespiratory test	Search modes - Boolean/Phrase	Interface - EBSCOhost Research Databases<br>Search Screen - Advanced Search<br>Database - MEDLINE	Display

S64	TX cardiopulmonary exercise test	Search modes - Boolean/Phrase	Interface - EBSCOhost Research Databases<br>Search Screen - Advanced Search<br>Database - MEDLINE	Display
S63	MH exercise test	Search modes - Boolean/Phrase	Interface - EBSCOhost Research Databases<br>Search Screen - Advanced Search<br>Database - MEDLINE	Display
S62	MH fibromyalgia OR MH chronic fatigue syndrome	Search modes - Boolean/Phrase	Interface - EBSCOhost Research Databases<br>Search Screen - Advanced Search<br>Database - MEDLINE	Display
S61	TX fibromyalgia	Search modes - Boolean/Phrase	Interface - EBSCOhost Research Databases<br>Search Screen - Advanced Search<br>Database - MEDLINE	Display
S60	S58 AND S59	Search modes - Boolean/Phrase	Interface - EBSCOhost Research Databases<br>Search Screen - Advanced Search<br>Database - AMED - The Allied and Complementary Medicine Database;CINAHL;MEDLINE	Display
S59	S54 OR S55 OR S56	Search modes - Boolean/Phrase	Interface - EBSCOhost Research Databases<br>Search Screen - Advanced Search<br>Database - AMED - The Allied and Complementary Medicine Database;CINAHL;MEDLINE	Display
S58	S53 OR S57	Search modes - Boolean/Phrase	Interface - EBSCOhost Research Databases<br>Search Screen - Advanced Search<br>Database - AMED - The Allied and Complementary Medicine Database;CINAHL;MEDLINE	Display
S57	myalgic encephalomyelitis	Search modes - Boolean/Phrase	Interface - EBSCOhost Research Databases	Display

			Search Screen - Advanced Search<br>Database - AMED - The Allied and Complementary Medicine Database;CINAHL;MEDLINE	
S56	S3 OR S4 OR S5 OR S6 OR S7 OR S8 OR S9 OR S10 OR S11 OR S12 OR S13 OR S14 OR S15 OR S16 OR S17 OR S18 OR S19 OR S20 OR S21 OR S22 OR S23 OR S24 OR S25 OR S26 OR S27 OR S28 OR S29	Search modes - Boolean/Phrase	Interface - EBSCOhost Research Databases<br>Search Screen - Advanced Search<br>Database - AMED - The Allied and Complementary Medicine Database;CINAHL;MEDLINE	Display
S55	S43 OR S44 OR S45 OR S46 OR S47 OR S48 OR S49 OR S50 OR S51	Search modes - Boolean/Phrase	Interface - EBSCOhost Research Databases<br>Search Screen - Advanced Search<br>Database - MEDLINE	Display
S54	S30 OR S31 OR S32 OR S33 OR S34 OR S35 OR S36 OR S37 OR S38 OR S39 OR S40 OR S41 OR S42	Search modes - Boolean/Phrase	Interface - EBSCOhost Research Databases<br>Search Screen - Advanced Search<br>Database - MEDLINE	Display
S53	S1 OR S2 OR S52	Search modes - Boolean/Phrase	Interface - EBSCOhost Research Databases<br>Search Screen - Advanced Search<br>Database - MEDLINE	Display
S52	TX chronic fatigue syndrome	Search modes - Boolean/Phrase	Interface - EBSCOhost Research Databases<br>Search Screen - Advanced Search<br>Database - MEDLINE	Display
S51	TX sarcopenia	Search modes - Boolean/Phrase	Interface - EBSCOhost Research Databases<br>Search Screen - Advanced Search<br>Database - MEDLINE	Display
S50	TX muscle cross-sectional area	Search modes - Boolean/Phrase	Interface - EBSCOhost Research Databases<br>Search Screen - Advanced	Display

			Search<br>Database - MEDLINE	
S49	TX Muscle volume	Search modes - Boolean/Phrase	Interface - EBSCOhost Research Databases<br>Search Screen - Advanced Search<br>Database - MEDLINE	Display
S48	TX Muscle mass	Search modes - Boolean/Phrase	Interface - EBSCOhost Research Databases<br>Search Screen - Advanced Search<br>Database - MEDLINE	Display
S47	TX fat mass	Search modes - Boolean/Phrase	Interface - EBSCOhost Research Databases<br>Search Screen - Advanced Search<br>Database - MEDLINE	Display
S46	TX Free fat mass	Search modes - Boolean/Phrase	Interface - EBSCOhost Research Databases<br>Search Screen - Advanced Search<br>Database - MEDLINE	Display
S45	TX lean body mass	Search modes - Boolean/Phrase	Interface - EBSCOhost Research Databases<br>Search Screen - Advanced Search<br>Database - MEDLINE	Display
S44	TX body composition	Search modes - Boolean/Phrase	Interface - EBSCOhost Research Databases<br>Search Screen - Advanced Search<br>Database - MEDLINE	Display
S43	MH body composition	Search modes - Boolean/Phrase	Interface - EBSCOhost Research Databases<br>Search Screen - Advanced Search<br>Database - MEDLINE	Display
S42	TX handgrip	Search modes - Boolean/Phrase	Interface - EBSCOhost Research Databases<br>Search Screen - Advanced Search<br>Database - MEDLINE	Display

S41	TX Handgrip test	Search modes - Boolean/Phrase	Interface - EBSCOhost Research Databases<br>Search Screen - Advanced Search<br>Database - MEDLINE	Display
S40	TX Handgrip assessment	Search modes - Boolean/Phrase	Interface - EBSCOhost Research Databases<br>Search Screen - Advanced Search<br>Database - MEDLINE	Display
S39	TX Maximal strength	Search modes - Boolean/Phrase	Interface - EBSCOhost Research Databases<br>Search Screen - Advanced Search<br>Database - MEDLINE	Display
S38	TX Knee contraction	Search modes - Boolean/Phrase	Interface - EBSCOhost Research Databases<br>Search Screen - Advanced Search<br>Database - MEDLINE	Display
S37	TX Knee maximal contraction	Search modes - Boolean/Phrase	Interface - EBSCOhost Research Databases<br>Search Screen - Advanced Search<br>Database - MEDLINE	Display
S36	TX Maximal isometric contraction	Search modes - Boolean/Phrase	Interface - EBSCOhost Research Databases<br>Search Screen - Advanced Search<br>Database - MEDLINE	Display
S35	TX Isometric contraction	Search modes - Boolean/Phrase	Interface - EBSCOhost Research Databases<br>Search Screen - Advanced Search<br>Database - MEDLINE	Display
S34	TX Maximum voluntary contraction	Search modes - Boolean/Phrase	Interface - EBSCOhost Research Databases<br>Search Screen - Advanced Search<br>Database - MEDLINE	Display
S33	MH Muscle strength	Search modes - Boolean/Phrase	Interface - EBSCOhost Research Databases<br>Search Screen - Advanced	Display

			Search<br>Database - MEDLINE	
S32	MH Muscle strength dynamometer	Search modes - Boolean/Phrase	Interface - EBSCOhost Research Databases<br>Search Screen - Advanced Search<br>Database - MEDLINE	Display
S31	Muscle strength dynamometer	Search modes - Boolean/Phrase	Interface - EBSCOhost Research Databases<br>Search Screen - Advanced Search<br>Database - MEDLINE	Display
S30	Muscle strength	Search modes - Boolean/Phrase	Interface - EBSCOhost Research Databases<br>Search Screen - Advanced Search<br>Database - MEDLINE	Display
S29	TX fitness test	Search modes - Boolean/Phrase	Interface - EBSCOhost Research Databases<br>Search Screen - Advanced Search<br>Database - MEDLINE	Display
S28	TX lactate	Search modes - Boolean/Phrase	Interface - EBSCOhost Research Databases<br>Search Screen - Advanced Search<br>Database - MEDLINE	Display
S27	TX lactate threshold	Search modes - Boolean/Phrase	Interface - EBSCOhost Research Databases<br>Search Screen - Advanced Search<br>Database - MEDLINE	Display
S26	TX Maximal test	Search modes - Boolean/Phrase	Interface - EBSCOhost Research Databases<br>Search Screen - Advanced Search<br>Database - MEDLINE	Display
S25	TX Submaximal test	Search modes - Boolean/Phrase	Interface - EBSCOhost Research Databases<br>Search Screen - Advanced Search<br>Database - MEDLINE	Display

S24	TX Aerobic Capacity	Search modes - Boolean/Phrase	Interface - EBSCOhost Research Databases<br>Search Screen - Advanced Search<br>Database - MEDLINE	Display
S23	TX Cardiorespiratory Fitness	Search modes - Boolean/Phrase	Interface - EBSCOhost Research Databases<br>Search Screen - Advanced Search<br>Database - MEDLINE	Display
S22	TX Ventilatory Threshold	Search modes - Boolean/Phrase	Interface - EBSCOhost Research Databases<br>Search Screen - Advanced Search<br>Database - MEDLINE	Display
S21	TX maximum oxygen uptake	Search modes - Boolean/Phrase	Interface - EBSCOhost Research Databases<br>Search Screen - Advanced Search<br>Database - MEDLINE	Display
S20	TX anaerobic threshold	Search modes - Boolean/Phrase	Interface - EBSCOhost Research Databases<br>Search Screen - Advanced Search<br>Database - MEDLINE	Display
S19	TX maximum oxygen uptake	Search modes - Boolean/Phrase	Interface - EBSCOhost Research Databases<br>Search Screen - Advanced Search<br>Database - MEDLINE	Display
S18	TX Peak oxygen uptake	Search modes - Boolean/Phrase	Interface - EBSCOhost Research Databases<br>Search Screen - Advanced Search<br>Database - MEDLINE	Display
S17	TX Aerobic assessment	Search modes - Boolean/Phrase	Interface - EBSCOhost Research Databases<br>Search Screen - Advanced Search<br>Database - MEDLINE	Display
S16	TX Aerobic Test	Search modes - Boolean/Phrase	Interface - EBSCOhost Research Databases<br>Search Screen - Advanced	Display

			Search<br>Database - MEDLINE	
S15	TX Incremental test	Search modes - Boolean/Phrase	Interface - EBSCOhost Research Databases<br>Search Screen - Advanced Search<br>Database - MEDLINE	Display
S14	TX maximum heart rate	Search modes - Boolean/Phrase	Interface - EBSCOhost Research Databases<br>Search Screen - Advanced Search<br>Database - MEDLINE	Display
S13	TX Heart Rate	Search modes - Boolean/Phrase	Interface - EBSCOhost Research Databases<br>Search Screen - Advanced Search<br>Database - MEDLINE	Display
S12	TX Aerobic fitness	Search modes - Boolean/Phrase	Interface - EBSCOhost Research Databases<br>Search Screen - Advanced Search<br>Database - MEDLINE	Display
S11	TX peak power	Search modes - Boolean/Phrase	Interface - EBSCOhost Research Databases<br>Search Screen - Advanced Search<br>Database - MEDLINE	Display
S10	TX Aerobic power	Search modes - Boolean/Phrase	Interface - EBSCOhost Research Databases<br>Search Screen - Advanced Search<br>Database - MEDLINE	Display
S9	TX Exercise performance	Search modes - Boolean/Phrase	Interface - EBSCOhost Research Databases<br>Search Screen - Advanced Search<br>Database - MEDLINE	Display
S8	TX functional capacity	Search modes - Boolean/Phrase	Interface - EBSCOhost Research Databases<br>Search Screen - Advanced Search<br>Database - MEDLINE	Display

S7	TX Cardiorespiratory	Search modes - Boolean/Phrase	Interface - EBSCOhost Research Databases<br>Search Screen - Advanced Search<br>Database - MEDLINE	Display
S6	TX physical fitness	Search modes - Boolean/Phrase	Interface - EBSCOhost Research Databases<br>Search Screen - Advanced Search<br>Database - MEDLINE	Display
S5	TX Cardiorespiratory test	Search modes - Boolean/Phrase	Interface - EBSCOhost Research Databases<br>Search Screen - Advanced Search<br>Database - MEDLINE	Display
S4	TX cardiopulmonary exercise test	Search modes - Boolean/Phrase	Interface - EBSCOhost Research Databases<br>Search Screen - Advanced Search<br>Database - MEDLINE	Display
S3	MH exercise test	Search modes - Boolean/Phrase	Interface - EBSCOhost Research Databases<br>Search Screen - Advanced Search<br>Database - MEDLINE	Display
S2	MH fibromyalgia OR MH chronic fatigue syndrome	Search modes - Boolean/Phrase	Interface - EBSCOhost Research Databases<br>Search Screen - Advanced Search<br>Database - MEDLINE	Display
S1	TX fibromyalgia	Search modes - Boolean/Phrase	Interface - EBSCOhost Research Databases<br>Search Screen - Advanced Search<br>Database - MEDLINE	Display
