# Supplementary material for: Fibromyalgia and Chronic Fatigue Syndromes: A systematic review and meta-analysis of cardiorespiratory fitness and neuromuscular function compared with healthy individuals
Source: PLoS One. 2022 Oct 20;17(10):e0276009. doi: 10.1371/journal.pone.0276009 (PMC9584387; doi:10.1371/journal.pone.0276009)
Supplement: S6 File — (PDF) [file pone.0276009.s006.pdf]

List of abbreviations

AT = Anaerobic threshold

BIA = Bio-impedance Analysis

CFS = Chronic Fatigue Syndrome

DXA= Dual-energy X-ray Absorptiometry

EPHPP = the Quality Assessment Tool for Quantitative Studies

FFM = Free Fat Mass

FM = Fat Mass

FMS = Fibromyalgia Syndrome

HR = Heart Rate

MM = Muscle Mass

MVC = Maximal Voluntary Contraction

PRISMA = Preferred Items for Systematic Reviews and Meta-Analyses

PROSPERO = International Prospective Register of Systematic Reviews

RPE = Rate of Perceived Exertion

SMD = Standardize Mean Difference

VA = Voluntary Activation

VO_2 Peak = Peak oxygen uptake
